# The structural diversity of CACTA transposons in genomes of *Chenopodium* (*Amaranthaceae*, *Caryophyllales*) species: specific traits and comparison with the similar elements of angiosperms

**DOI:** 10.1186/s13100-022-00265-3

**Published:** 2022-04-04

**Authors:** Alexander Belyayev, Jiřina Josefiová, Michaela Jandová, Ruslan Kalendar, Václav Mahelka, Bohumil Mandák, Karol Krak

**Affiliations:** 1 Czech Academy of Sciences, Institute of Botany, Zámek 1, CZ-252 43 Průhonice, Czech Republic; 2 Institute of Biotechnology HiLIFE, University of Helsinki, P.O. Box 65, FI-00014 Helsinki, Finland; 3 National Laboratory Astana, Nazarbayev University, 53 Kabanbay batyr Ave., 010000, Nur-Sultan, Kazakhstan; 4 Faculty of Environmental Sciences, Czech University of Life Sciences Prague, Kamýcká 129, 165 00 Suchdol, Praha Czech Republic

**Keywords:** CACTA transposons, *Chenopodium*, Flowering plants, Next generation sequencing, Genome evolution

## Abstract

**Background:**

CACTA transposable elements (TEs) comprise one of the most abundant superfamilies of Class 2 (cut-and-paste) transposons. Over recent decades, CACTA elements were widely identified in species from the plant, fungi, and animal kingdoms, but sufficiently studied in the genomes of only a few model species although non-model genomes can bring additional and valuable information. It primarily concerned the genomes of species belonging to clades in the base of large taxonomic groups whose genomes, to a certain extent, can preserve relict and/or possesses specific traits. Thus, we sought to investigate the genomes of *Chenopodium* (*Amaranthaceae*, *Caryophyllales*) species to unravel the structural variability of CACTA elements. *Caryophyllales* is a separate branch of Angiosperms and until recently the diversity of CACTA elements in this clade was unknown.

**Results:**

Application of the short-read genome assembly algorithm followed by analysis of detected complete CACTA elements allowed for the determination of their structural diversity in the genomes of 22 *Chenopodium album* aggregate species. This approach yielded knowledge regarding: (i) the coexistence of two CACTA transposons subtypes in single genome; (ii) gaining of additional protein conserved domains within the coding sequence; (iii) the presence of captured gene fragments, including key genes for flower development; and (iv)) identification of captured satDNA arrays. Wide comparative database analysis revealed that identified events are scattered through Angiosperms in different proportions.

**Conclusions:**

Our study demonstrated that while preserving the basic element structure a wide range of coding and non-coding additions to CACTA transposons occur in the genomes of *C. album* aggregate species. Ability to relocate additions inside genome in combination with the proposed novel functional features of structural-different CACTA elements can impact evolutionary trajectory of the host genome.

**Supplementary Information:**

The online version contains supplementary material available at 10.1186/s13100-022-00265-3.

## Background

CACTA transposable elements comprise one of the most abundant superfamilies of Class 2 (cut-and-paste) transposons. First identified in plants, the CACTA element *Suppressor-mutator* (*Spm*) was analyzed in detail by Barbara McClintock and Peter Peterson in the *Zea mays* (*Poaceae*, *Poales*) genome [1–3]. Over recent decades, CACTA elements were widely identified in species from the plant, fungi, and animal kingdoms. Among Angiosperms, data has accumulated on several complete CACTA elements acquired from a range of model di- and monocotyledon species ([4–11], for review see [12, 13]).

The CACTA transposons were named after a highly conserved motif at the element ends. The ends contain the sequences that are required *in cis* for transposition and the internal region contains the genes for the *trans*-acting proteins. A full-length CACTA element consists of two terminal inverted repeats (TIRs) and subterminal repeats (subTIRs) of several hundred nucleotides containing closely spaced direct and inverted repeats. From the two open reading frames (ORFs), one encodes a transposase (TPase) and the other a protein of unknown function [14]. The TPase binds to the TIR during excision, creating a 3-bp target site duplication [12]. The catalytic center of the TPase is the acidic triad known as the “DDD/E” motif, which is highly conserved [15].

The key feature of CACTA elements (as well as all TEs in general) is their ability to influence the evolution of the host genome. This may happen in several ways, such as: (i) via alterations of gene function through insertion; (ii) through the induction of chromosomal rearrangements; and (iii) as a source of coding and noncoding material that allows for the emergence of genetic novelty (such as new genes and regulatory sequences) [16]. Several typical examples can illustrate each of the above points. Thus, CACTA elements affect flower color in *Glycine max* (*Fabaceae*, *Fabales*) [17]; CACTA elements can be found in the hot spots of large chromosomal rearrangements in genotypes from marginal populations of *Aegilops speltoides* (*Poaceae, Poales*) [18]; and CACTA elements generate novel exon combinations in the genome of *G. max* [19]. The mentioned evolutionary events are highly valuable and therefore determination of the entire fan of structural and genomic variability of CACTA elements is essential for understanding genome evolution at the molecular level.

Exploration of transposable elements (TEs) is more challenging than a single-copy gene. Boundaries of the element sequence must be determined, and all variants of the element must be identified. There are many problems at this stage, since TEs are often nested, and a mixed sequence can be mistakenly recognized as a new element. For CACTA elements in particular, the low sequence conservation of TIRs and subTIRs makes the identification of these elements difficult. Therefore, despite decades of investigation, CACTA elements have been studied in the genomes of only a few model plant species although non-model genomes can bring additional and valuable information. It primarily concerned the genomes of plant species belonging to clades in the base of large taxonomic groups whose genomes, to a certain extent, can preserve relict and/or possesses specific traits. Qualitative and quantitative characteristics of the mobilome in these genomes may be the key to understanding the divergent evolution of flowering plants at the whole-genome level and the structural and functional changes in individual components of TEs fraction. To at least partially fill this knowledge gap, we extensively explore the genomes of *Chenopodium* s. str. (also referred to as the *Chenopodium album* aggregate) species (*Amaranthaceae*, *Caryophyllales*) to unravel the structural and genomic variability of CACTA-like elements. Species of the *C. album* aggregate (agg.) (that are the wild relatives of economically important Quinoa) are distributed worldwide, with the highest species diversity in temperate areas [20]. The majority of the species in this diploid-polyploid complex are phenotypically exceptionally plastic and are able to grow under a wide range of conditions [21]. The early differentiation of the *C. album* agg. coincided with the beginning of the Miocene Climatic Optimum ~ 20 Mya. Clade H (for the phylogeny of species and definition of the clades see S1) separated upon the transition between the Serravallian and Tortonian Ages, ~ 11 Mya. However, the main lineages were formed in the Pliocene. Subsequent speciation within the lineages and the appearance of the majority of polyploids took place in the Quaternary Period [22]. The group of flowering plants that have been investigated is unique in many characteristics. For example, sieve tube plastids of *Caryophyllales* are of a type (P-III) found in no other seed plants [23]. Consequently, in their phylogenetic systems, both Takhtajan [23] and Cronquist [24] identified *Caryophyllales* (=*Caryophyllidae*) as a separate branch that derived directly from the basic *Magnoliidae*. The APG IV classification system also confirms the *Caryophyllales* special status as the member of “superasterids” at the base of the monophyletic “asterids” group [25]. Until recently, no complete CACTA-like elements were described for any *Caryophyllales.* Several examples refer to detection of a *tnp2* conserved domain nested within the putative LTR retrotransposon in the *Beta vulgaris* genome (*Amaranthaceae*, *Caryophyllales*) [26, 27]. Fragments and non-autonomous CAСTA elements were found as inclusions into mutable genes that change the flowers color in the genomes of *Mirabilis jalapa* (*Nyctaginaceae, Caryophyllales*) and *Dianthus caryophyllus* (*Caryophyllaceae, Caryophyllales*) thus engaged in phenotypic variations [28, 29]. The first complete CACTA-like element *Jozin* was discovered by us in the genome of *C. pamiricum* (*Amaranthaceae*, *Caryophyllales*) [30], but is there any difference between CACTA elements of typical representatives of the *Caryophyllales* branch and the rest of the Angiosperms [14], including the most basal lineage of *Amborella trichopoda* [25]?

## Results

### TPase domain-based phylogenetic analysis of CACTA-like elements

**Fig. 1:**
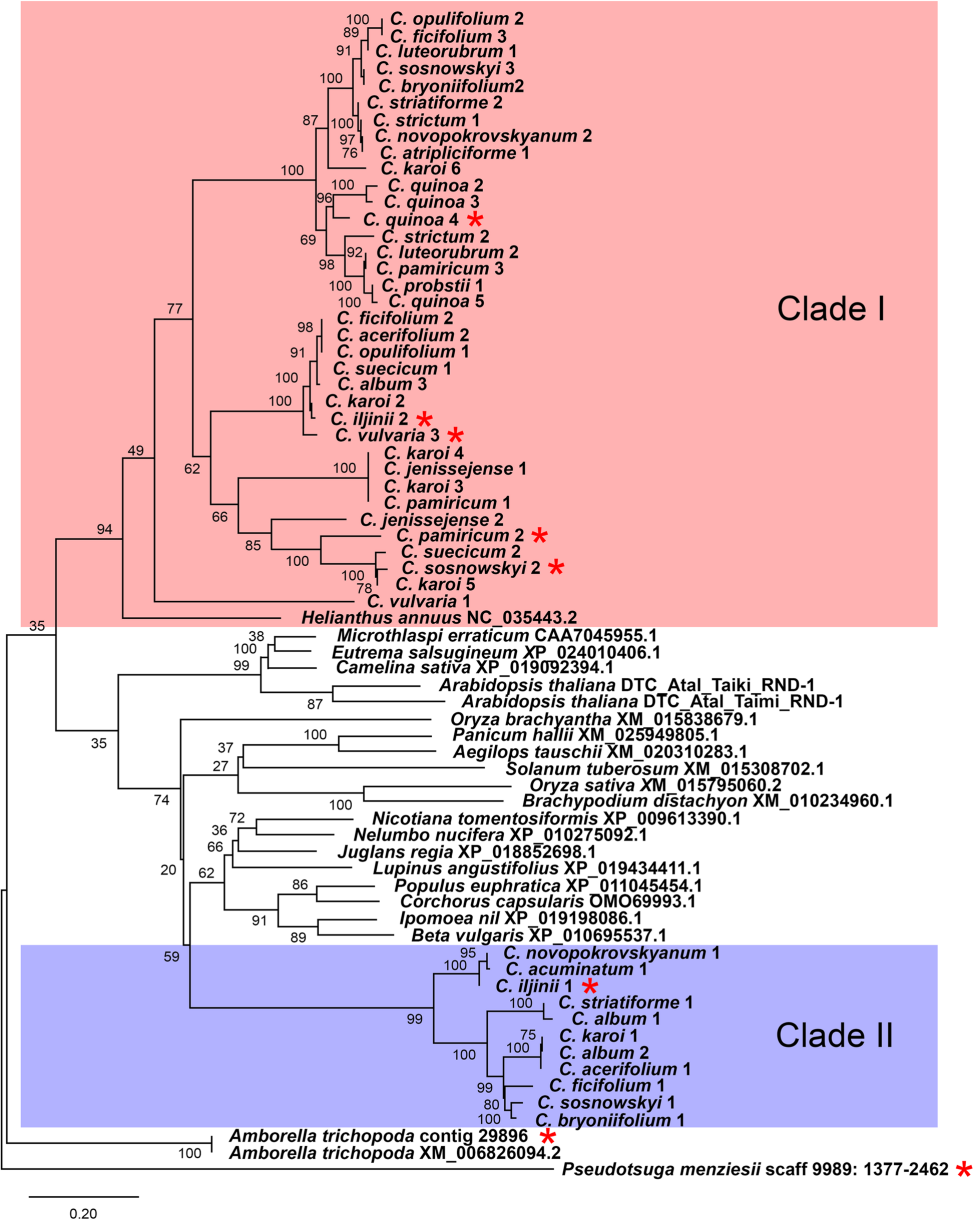
Phylogenetic relationships of conserved protein domains of the *tnp2* TPase family. Clade 1 is highlighted in red; Clade 2 is highlighted in blue. The numbers follow the *Chenopodium* species name corresponding to accession numbers and the numbers of contigs (S2). GenBank accession numbers follow the plant species name. Contigs/scaffolds used for further Conserved Domain Architecture (CDA) analysis (see below) are marked with red asterisks

As a first step towards understanding the natural variability of CACTA superfamily elements, we performed a comparative analysis of TPase, its most conservative region. Transposase is an enzyme that mediates the excision of the element from its donor location and its reintegration into a new chromosomal locus [31]. To fulfil this task, we retrieved highly conserved TPase domains from low coverage short read sequencing based contigs of 22 *C. album* agg. species (59 genotypes in total, S1). Hereinafter, the names and numbers of the contigs are given in S2. The typical full-length *Transposase_21* super-family *tnp2* family domain was approximately 630 bp (310 aa) [30]. We identified 47 full-length *tnp2* domains in *Chenopodium* species. Additionally, 23 *tnp2* domains from genomes of the species from different taxonomic groups were retrieved for comparative analysis from publicly available genomic sequence data (Fig. 1 and S2). All analyzed TPases belonged to *pfam02992*, a member of the *cl29371* superfamily. To understand the specific features of TPase domains of CACTA-like elements in the genomes of *C. album* agg. species, we further constructed a maximum likelihood (ML) phylogenetic tree. TPases of investigated *Chenopodium* species were grouped into two well separated major clades that differed distinctly from most of the TPase domains of the other plant groups (Fig. 1). Clade I was composed of 36 sequences of 19 *Chenopodium* species, with *Helianthus annuus* (*Asteraceae, Asterales*) as a basal taxon of the group. Clade II of *Chenopodium* sequences was composed of 11 sequences from 10 species. Although the relationship of Clade II with other Angiosperm lineages is unclear, due to the low bootstrap support values of deep branches of the tree, the isolated position of the group is obvious. Within both Clades of *Chenopodium* there were multiple subclades that further resolve the relationships of the TPase sequences. It is worth mentioning that one genotype can possess conservative domains from both clades (Fig. 1). Two TPase domains from the basal angiosperm *Amborella* form the base of the phylogenetic tree.

### Description of the CACTA-like element *Jozin* in genomes of *C. pamiricum*, *C. sosnowskyi*, *C. iljinii* and *C. vulvaria,* and comparison with the similar elements from assembled genome of *C. quinoa*

**Fig. 2:**
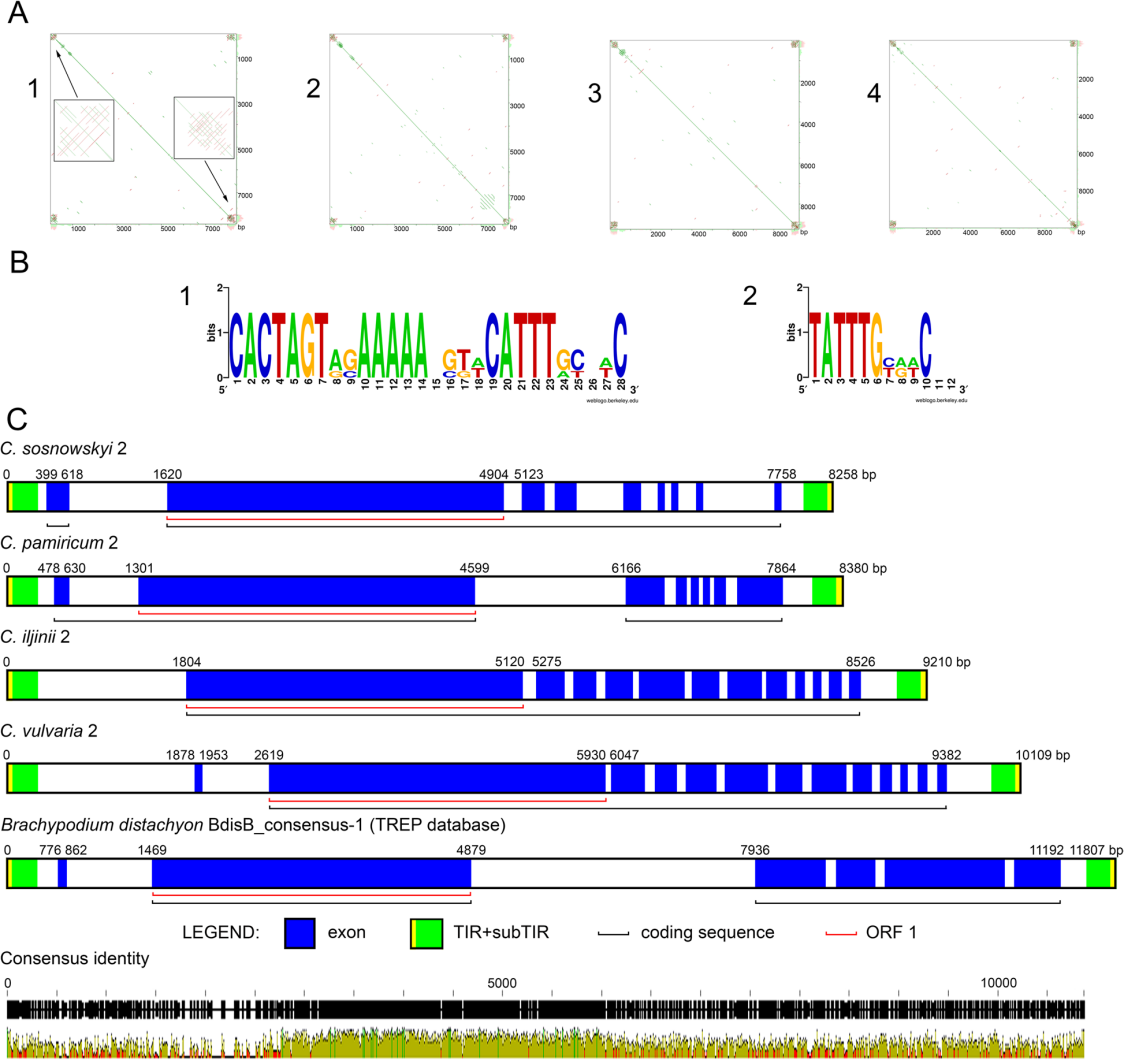
Analysis of the CACTA-like element *Jozin* in the genomes of *C. album* agg. species. **A** Self-to-self comparisons of the four CACTA-like complete elements from genomes of (1) *C. pamiricum*; (2) *C. sosnowskyi*; (3) *C. iljinii*; and (4) *C. vulvaria* (YASS program output). Enlarged TIRs + subTIRs are shown in separate boxes in (1). **B** Graphical representation of the TIRs and subTIR by sequence logo. (1) TIRs; (2) subTIR monomers. **C** AUGUSTUS-based diagrams of the complete CACTA-like TE *Jozin* from genomes four *Chenopodium* species and *B. distachyon* (the latter for comparison). Consensus identity of the four CACTA-like TEs from genomes of *Chenopodium* species is shown at the bottom

We next performed a full-length element analysis. The approach for the identification of new elements was based on their TIR/subTIR sequences [11] and the presence of putatively functional ORF 1. A priori, both TIRs must contain an intact CACTA motif and subTIRs must consist of 10- to 20-bp units repeated in direct and inverted orientation, thus forming a specific structure revealed by dot-plot analysis (Fig. 2)A1.

During the investigation of Illumina reads assembled to contigs, full-length complete-end and putatively functional CACTA-like elements were found in the genomes of *C. pamiricum* (830-3C), *C. sosnowskyi* (788–3), *C. iljinii* (433–9), and *C. vulvaria* (771–1) (Fig. 2A1-4). Sequences of the CACTA-like element *Jozin* for the genomes of the first two species were provided by us earlier [30]. Sequences of CACTA-like element *Jozin* from genomes of *C. iljinii* and *C. vulvaria* are provided in S3. TIRs were the following: (i) *C. pamiricum* CACTAGTAGAAA AAACGTCATTTGTAAC; (ii) *C. sosnowskyi* CACTAGTGGAAAAAAGTTCATTTGCAAC; (iii) *C. iljinii* CACTAGTAGAAAAATGTACATTTGCGTC; and (iv) *C. vulvaria* CACTAGTACAAAAACGTACATTTACTTC (Fig. 2)B1. The subTIR regions of *Jozin* elements were highly structured and contain (i) in *C. pamiricum* 19 copies of a 12-bp motif TATTTGTAACTA (similarity 83.3–100%), of which 9 reside in the 5′-end and 10 reside in the 3′-end; (ii) in *C. sosnowskyi* 19 copies of a 12-bp motif TATTTGTAACGG (similarity 91.7–100%), 11 in the 5′-end and 8 in the 3′-end; (iii) in *C. iljinii* 23 copies of a 12-bp motif TATTTGCGTCAC (similarity 91.7–100%), 10 in the 5′-end and 13 in the 3′-end; and (iv) in *C. vulvaria* 21 copies of a 12-bp motif TATTTGCGTCAC (similarity 83.3–100%), 10 in the 5′-end and 11 in the 3′-end (Fig. 2B2).

ORF 1 lengths were from 3296 bp to 3316 bp (S4). Pair-wise distances between ORF 1 at the protein level ranged from 0.22 between *C. pamiricum* and *C. sosnowskyi* to 0.583 between *C. pamiricum* and *C. iljinii* (S5). A putative functional ORF 1 contains the following four conservative domains (CDs): Transposase Associated Domain (*TAD*), Transposase 21 domain (*Transposase_21*), and two domains of unknown function (*DUF4218* and *DUF4216*). All these CDs were found within the ORFs 1 of the analyzed *Jozin* elements from genomes of four *Chenopodium* species (S4). In addition to similarities in nucleotide composition, the lengths and interpositions of the four CDs were very similar.

For justification of obtained results and comparison of complete CACTA elements we performed search in publicly available assembled genome of related species of *C. quinoa* (*Chenopodium quinoa* v1.0 (Quinoa)). We found 17 putative elements with complete ORF 1, but only three of them possessed subTIRs, and only one TIRs and subTIRs. The latter was CACTA element in scaffold 3500, position of TPase 473,953–475,341, and 9486 bp long. TIRs were as following: CACTAGTAGAAAAATAGAAATAGGCAAC that show 75% identity with TIRs of *C. iljinii* and *C. pamiricum* (100% similarity in the first 14 nucleotides). The same 75% similarity was determined between TIRs of *C. iljinii* and *C. pamiricum* and *C. vulvaria* that is sufficient for the fast-evolving TIRs to assent close relatedness between CACTA elements from genome of *C.quinoa* and from genomes of *C. album* agg. species. ORF 1 length was 3186 bp. Pair-wise distances between ORF 1 at the protein level was around 0.5 with previously investigated complete CACTA elements from *C. album* agg. species (S5). A putative functional ORF 1 contains the same four CDs: *TAD*, *Transposase_21*, *DUF4218* and *DUF4216*.

Despite the similarity in the length and interpositions of the CDs and lengths of ORFs 1 (Fig. 2C, S4), the sizes of the complete elements were significantly different. Thus, lengths of the *Jozin* element from the genome of *C. sosnowskyi* was 8258 bp, 8380 bp in *C. pamiricum*, 9210 bp in *C. iljinii*, 10,109 bp in *C. vulvaria,* and 9486 bp in the scaffold 3500 of the assembled genome of *C. quinoa*. These data point to structural variability that causes the differences in the lengths of elements.

### Conserved domains architecture variability in *C. album* agg. species and determination of the prevalence of CDA types among angiosperms

The annotation of protein sequences with the location of CDs is a common practice in the analysis of sequence data [32]. The eukaryotic gene prediction platform AUGUSTUS allowed detection of coding sequences and provided insights into their structure (see Materials and Methods). The results of the AUGUSTUS analysis of the complete CACTA-like elements of four *Chenopodium* species are shown in Fig. 2C. For comparison, the same type of analysis was performed on a complete CACTA-like element from the *Brachypodium distachyon* genome (TREP database) [33]. A successive CD search within the protein coding sequence determined by AUGUSTUS revealed cases of additional CDs presence in the ORF 2 region. We identified four types of the conserved domain architecture (CDA) of putative CACTA-like elements (S4).

**Fig. 3:**
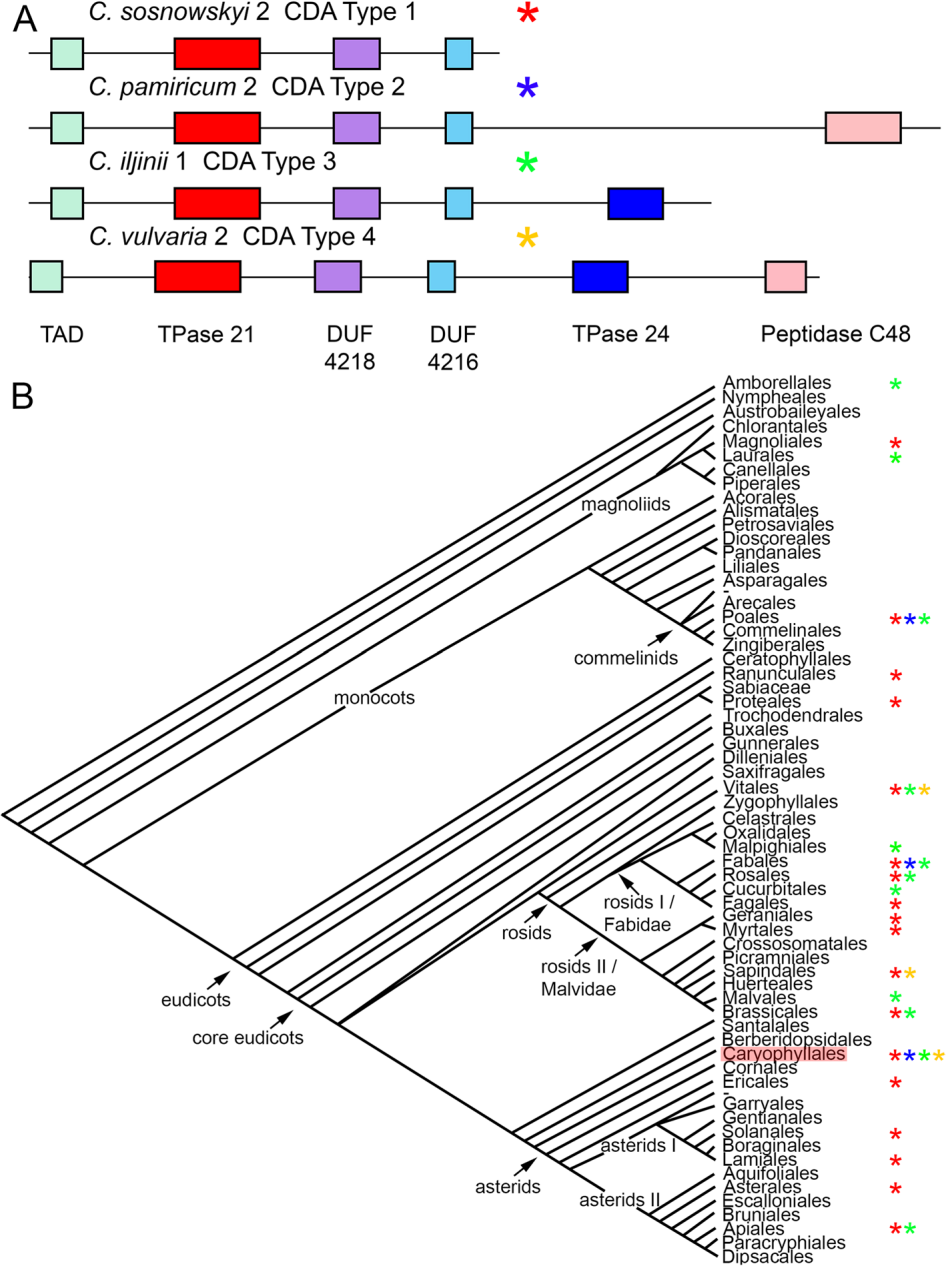
Structural variability of the CACTA-like element *Jozin* in the genomes of *C. album* agg. species. **A** Diagrams of the four CDA Types. **B** Distributions of four CDA Types across the Angiosperm phylogenetic tree (the APG IV taxonomic system of flowering plant was used). Asterisks correspond to CDA types. *Caryophyllales* is highlighted in red

In the CDA Type 1, only four CDs were present within the coding sequence of the element, namely *TAD*, *Transposase_21*, *DUF4181*, and *DUF4216*. Thus, CDs were present in ORF 1 only. Such a CDA type was found in the shortest element from the genome of *C. sosnowskyi* (S4 and Fig. 3A). In addition to the four above, in the CDA Type 2 one more CD was present in ORF 2, namely domain of *Peptidase_C48* superfamily (pfam02902). Such a CDA type was found in the element from the genome of *C. pamiricum* (S4 and Fig. 3A). It should be noted that the sizes of CACTA-like elements of Types 1 (*C. sosnowskyi*) and 2 (*C. pamiricum*) differed exactly by the lengths of the *Peptidase_C48* fragment (122 bp), thus indicating additional CD presence. Besides the four “regular” CDs of ORF 1, in the CDA Type 3 one more CD was present in ORF 2, namely domain of *Transposase_24* superfamily (*pfam03004*). Such a CDA type was found in the element with partly complete ends from the genome of *C. iljinii* (S4 and Fig. 3A). The CDA Type 4 contained all of the CDs mentioned above. Such a CDA type was found in the element from the genomes of *C. iljinii* and *C. vulvaria* (S4 and Fig. 3A).

We next determined the prevalence of CDA types among Angiosperms. This task was accomplished with the Conserved Domain Architecture Retrieval Tool (CDART) [34]. An NCBI conserved domain database (CDD) [35] search revealed the presence of CDA Type 1 in all major clades of dicotyledons (for *Caryophyllales* we provide data for the first time) and in *Poales* of monocotyledons. In total we obtained 671 database hits. Additionally, we analyzed several CACTA-like elements with almost complete ends (subTIRs are detectable) in publicly available assembled genomes. These elements were retrieved by positioning of *Transposase_21* CD by Domain based ANnotation of Transposable Elements (DANTE) [36] followed by TIRs/subTIRs search in adjacent sequences by YASS (see Material and Methods). We analyzed 11 CACTA-like elements from the TREP database (four complete are shown in S4), and six elements from publicly available assembled genomes (four complete are shown in S4) for the presence of CDs within the coding sequence determined by AUGUSTUS (S4). Thus, CDA Type 1 was identified in the *B. vulgaris* (*Amaranthaceae, Caryophyllales*) genome, chromosome 7, position of *Transposas_21* CD 17857865–17,859,202 (incomplete element ends); in *C. quinoa* genome scaffold 2306 position of *Transposase_21* CD 2306:2210197–2,211,585; and in *Magnolia ashei* (*Magnoliaceae, Magnoliales*) position of *Transposase_21* CD 1362–2581. We also identified a similar CDA in the assembled genome of Gymnosperm *P. menziesii* scaffold 9989 position of *Transposase_21* CD 1377–2462, although *TAD* and *Transposase_21* CDs were incomplete (S4). In complete CACTA-like elements provided by TREP database, CDA Type 1 was identified in *B. distachyon* BdisB_consensus-1, *Oryza sativa* (*Poaceae, Poales*) Dorian_consensus-1 and Grover_consensus-1, *Sorghum bicolor* G16–G18_consensus-1, (*Poaceae*, *Poales*) (S4), and *Metrosideros polymorpha* (*Myrtaceae, Myrtales*) A_consensus-1. A BLAST search with the Type 1 and extraction of the best hits obtained for the representatives of Angiosperms additionally revealed this CDA in the genomes of *H. annuus* (NC 035443), *G. max* (NC 038247), *Vitis riparia* (*Vitaceae, Vitales*) (NC 048435), and *Nelumbo nucifera* (*Nelumbonaceae*, *Proteales*) (NW 010729199).

CDA Type 2 is much rarer than CDA Type 1. Among the investigated *Chenopodium* species, CDA Type 2 was identified in the genome of *C. pamiricum* (S4 and Fig. 3A). In the NCBI CDD, only one hit from the genome of *Trifolium subterraneum* (*Fabaceae*, *Fabales*) (GenBank: GAU38063.1) was recorded so far. In complete CACTA-like elements provided by the TREP database, CDA Type 2 was identified in *O. sativa* Calvin_consensus-1 (although *TAD* is absent).

The CDA Type 3 is more common than the previous Type 2. Among the investigated *Chenopodium* species, CDA Type 3 was identified in the genome of *C. iljinii* (S4 and Fig. 3A). In the NCBI conserved domain database, 18 hits were recorded from the genomes of *Brassica rapa* (*Brassicaceae, Brassicales*) (GenBank: RIA04087.1), *Cucumis melo* (*Cucurbitaceae*, *Cucurbitales*) (GenBank: TYK07512.1, TYK08453.1, TYK17902.1, KAA0054101.1, KAA0038958.1, KAA0036985.1, TYK29391.1, TYK29391.1), *Daucus carota* (*Apiaceae*, *Apiales*) (GenBank: XP_017228957.1), *Gossypium barbadense* (*Malvaceae*, *Malvales*) (GenBank: PPD97216.1), *Prunus dulcis* (*Rosaceae*, *Rosales*) (GenBank: BBH05673.1), *T. subterraneum* (GenBank: GAU44176.1), and *V. vinifera* (GenBank: RVW26966.1, RVW66324.1, RVW69569.1, RVW71229.1, RVW88229.1, RVW97993.1). Additionally, after analysis of several CACTA-like elements with almost complete ends (subTIRs were detectable) in publicly available assembled genomes, CDA Type 3 was identified in the genome of *C. quinoa* and most importantly in the genome of *Amborella trichopoda* (*Amborellaceae*, *Amborellales*) (S4). It should also be noted that both *Transposase_21* of CACTA-like elements with CDA of Type 3 (*C. iljinii* and *C. quinoa*) were classified as belonging to the TPase domain-based Clade I (see above and Fig. 1). In complete CACTA-like elements provided by the TREP database, CDA Type 3 was identified in *Hordeum vulgare Caspar* AY661558–1 (S4), *Arabidopsis thaliana* (*Brassicaceae, Brassicales*) Taiki_RND-1, and Taimi_RND-1. A BLAST search with Type 3 and extraction of the best hits obtained for the representatives of Angiosperms additionally revealed this CDA in the genomes of *Theobroma cacao* (*Malvaceae*, *Malvales*) (NC 030857), *Populus trichocarpa* (*Salicaceae*, *Malpighiales*) (NC 037301), and, again, *A. trichopoda* (NW 006497717).

CDA Type 4 (except for “regular” CDs *Transposase_24* and *Peptidase_C48*) was present within the coding sequence of CACTA-like elements in the genomes of *C. iljinii* and *C. vulvaria* (S4 and Fig. 3A). No such combination of domains was found in the NCBI conserved domain database. In the genome of *V. vinifera*, *Transposase_21*, *DUF4218*, *DUF4216*, *Transposase_24*, and *Peptidase_C48* were present but *TAD* was absent (GenBank: RVW26095.1, etc.). In the genome of hybrid *Citrus clementina* (*Rutaceae*, *Sapindales*) (GenBank: XP_024037655.1, XP_006429639.2), *TAD*, *Transposase_21*, *DUF4218*, *Transposase_24*, and *Peptidase_C48* were present but *DUF4216* was absent. The distribution of CDA types in the angiosperm phylogenetic tree is shown in Fig. 3B.

### Experimental validation of computationally identified CDA types

**Fig. 4:**
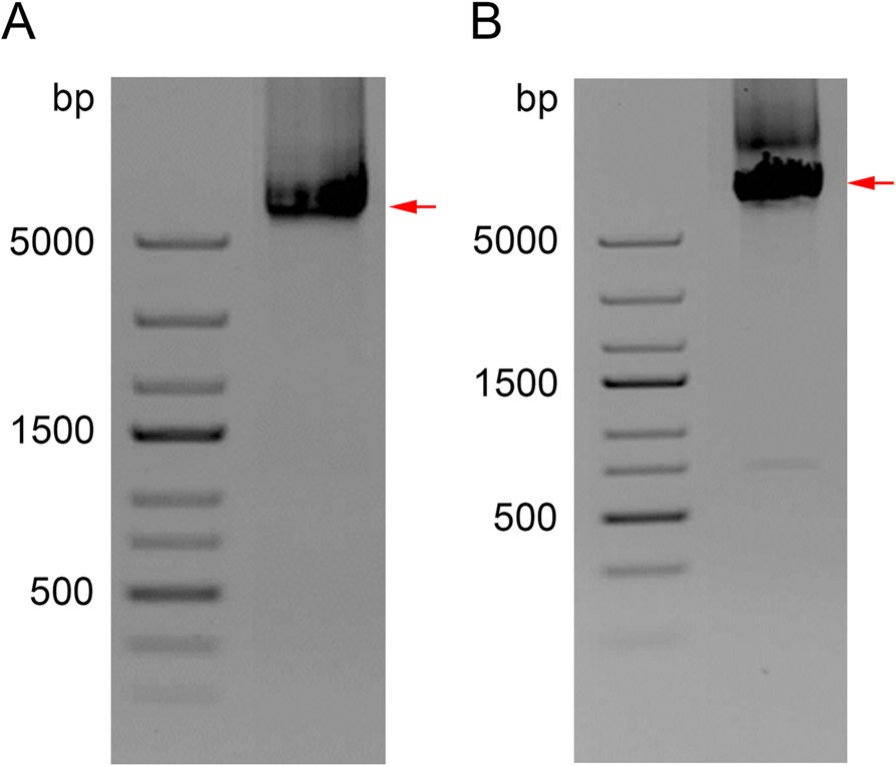
Experimental validation of the computationally identified CDA. **A** PCR screening for presence in the genome of *C. iljinii* CDA Type 3 (*C. iljinii 1* contig). Combination of primers ## 1–3 (S7). Clone Tp24-X1_T7 (S6), GenBank # MZ325224. **B** PCR screening for presence in genome of *C. iljinii* CDA Type 4 (*C. iljinii 2* contig). Combination of primers ## 4–6 (S7). Clone 68TAD-X1 (S6), GenBank # MZ325225

The generation of consensus sequences for TEs by assembling reads and their subsequent annotation may be challenging. For example, the question arises of weather genome sequencing using low coverage (< 1×) is suitable to estimate correctly the sequence diversity of a given TE superfamily? Thus, to answer questions about the accuracy of the method, and to avoid artifacts of CDA annotation procedures, we sought to confirm the existence of the physical counterparts of computer-generated CDAs in the genome of *C. iljinii*, the species with a high level of CACTA-like elements and with different gained CDs. If the annotation is correct, then the assembled sequences should not differ from the cloned by large values. For this analysis, we choose two contigs: *C. iljinii* 1 (CDA Type 3) and *C. iljinii* 2 (CDA Type 4) (S2, S4) that contains a CACTA-like element. According to AUGUSTUS analysis, the *C. iljinii* 1 contig gained *Transposase_24* CD (detected within the coding sequence) and the *C. iljinii* 2 contig contained a complete CACTA-like element with gained *Transposase_24* and *Peptidase_C48* CDs (S4). Comparative analysis of contigs and clones is given in S6. According to TPase-based phylogenetic analysis, these two elements belonged to different clades (see above). To amplify the inner part of the *Jozin* transposons, primers were designed on the basis of consensus contig sequences (see Material and Methods and S7). The primers amplified DNA fragments of the expected sizes (Fig. 4). Among several clones amplified with primers on the basis of *C. iljinii* 1 contig, clone Tp24-X1 (GenBank # MZ325224) exhibited the presence of CDs specific for the CACTA-like element. The length of the clone was 5778 bp. Analysis of coding sequence of clone Tp24-X1 performed by AUGUSTUS revealed the presence of the following CDs within the coding sequence: *TAD*, *Transposase_21*, *DUF4218*, *DUF4216*, and *Transposase_24*. Aligning the assembled *C. iljinii* 1 contig and clone Tp24-X1 revealed 91.1% similarity, with the following similarities for the corresponding intervals of separate CDs: 98.5% for *TAD*; 96.2% for *Transposase_21*; 97.5% for *DUF4218*; 96.1% for *DUF4216*, and 91.7% for *Transposase_24*. Among several clones amplified with primers on the basis of the *C. iljinii* 2 contig, clone 68TAD-X1 exhibited the presence of CDs specific for the CACTA-like element. The length of the clone was 6586 bp. Analysis of the coding sequence of clone 68TAD-X1 (GenBank # MZ325225) determined by AUGUSTUS revealed the presence of the following CDs: *TAD*, *Transposase_21*, *DUF4218*, *DUF4216*; *Transposase_24*, and *Peptidase_C48*. Aligning the assembled C. iljinii 2 contig and clone 68TAD-X1 revealed 97.2% similarity with the following similarities for the corresponding intervals of separate CDs: 99.2% for *TAD*: 99.2% for *Transposase_21*; 96.9% for *DUF4218*; 99.5% for *DUF4216*; 100% for *Transposase_24*, and 99.0% for *Peptidase_C48*. Thus, the physical existence of different CDA Types 3 and 4 in a single genome and the accuracy of the assembly based on low coverage sequencing were confirmed. It also should be noted that pairwise identity of clones Tp24-X1 and 68TAD-X1 that represent the different subtypes of CACTA elements was low (51.6%).

### Captured gene fragments

**Fig. 5:**
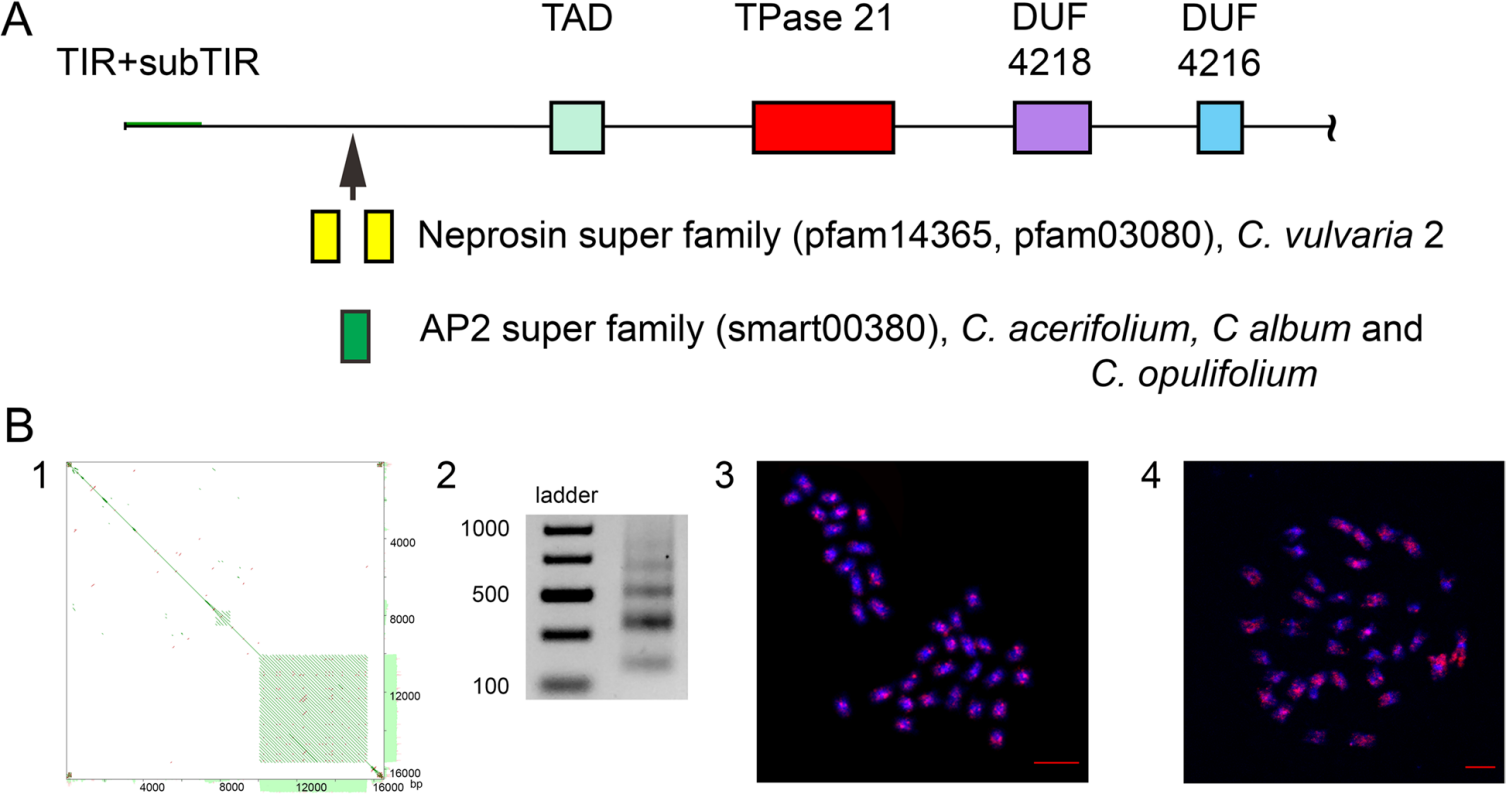
Additions to CACTA transposons. **A** Diagrams of the captured gene fragments. **B** Captured satDNA. (1) Self-to-self comparisons of the CACTA-like element from the genome of *C. quinoa* (scaffold 3389) with the captured satDNA array. Parallel lines indicate tandem repeats (the distance between the diagonals equals the lengths of the motifs). (2) Agarose gel electrophoresis of PCR products obtained with primers designed from consensus monomer sequence of novel satDNA family from the genome of *C. sosnowskyi* showing typical ladder structure of tandem array. (3–4) FISH showing distribution of the novel satDNA family sequences on the chromosomes of *C. quinoa* and *C. sosnowskyi*. Novel satDNA is labeled with Cy3 (red signal); chromosomes are stained with DAPI (blue signal). The bar represents 5 μm

In addition to gained CDs, the fragments of host genes were found within the CACTA-like elements in the genomes of the explored *Chenopodium* species, which can also affect the length of the element. The most apparent was detected by a BLAST CD search for the presence of two fragments of the Neprosin superfamily (*pfam14365* and *pfam03080*) in the CACTA-like element with complete ends from the genome of *C. vulvaria* (Fig. 5A). The fragments were positioned from 2894 to 3007 and from 3254 to 3457 in the 5’ region of the element. AUGUSTUS analysis confirmed the results of a BLAST CD search by showing the presence of a nonfunctional exon in the same position. The CACTA-like element from the genome of *C. vulvaria* was the longest found, and the gene fragments were located 754 bp upstream the *TAD* CD in the 5’ region of the element. Three more examples of gene capture by CACTA-like elements were also identified (Fig. 5A). Thus, in the genome of *C. acerifolium* (accession 316–1, *C. acerifolium 2* contig), putative capture of the *Apetala 2* (AP2) 80 bp gene fragment that was located 857 bp upstream *TAD* CD was identified. The same 80 bp AP2 gene fragments were also found in the genomes of *C. album* (accession 291–1, *C. album 3* contig, 1331 bp upstream of *TAD* CD) and *C. opulifolium* (accession 696–6, *C. opulifolium 1* contig, 1342 bp upstream of *TAD* CD). All three elements were with almost complete 5’ ends (distinguishable subTIRs), and all captured gene fragments were located outside of the element coding sequence in its 5’ region. Similarity between determined 80 bp AP2 fragments in three different species was 100%.

### Presence of tandem repeats within CACTA element

In further CDs and captured gene fragments, we found more additions to the CACTA-like element *Jozin* structure. Using the assembled genome of *C. quinoa* with the help of the DANTE pipeline and YAAS program, we identified a CACTA-like element with almost complete ends in the scaffold 3389 (position of *Transposase_21* CD 1928329–1929717). The length of the element was 16523 bp, with the coding sequence from 1676 to 9409. A satDNA array was found inside the element (Fig. 5)B1. The length of the array was 5550 bp in the interval from 10098 to 15648 and contained 32.5 monomers with a consensus sequence of 171 bp (GCCATTTTGGTTGAAATTGCGTCCCAAAACCTCTTACTTGCTCTTTTCTTGTAATC TCAAGCATTAGTCACCTATAACTAAGTGATAATGACATGTTTTAACTAGTTATGAGCAATAGACATGAATTGTAGAAAATAGGCATCCTAGTAGCCAAAATGGCCTTACTTGA). This satDNA array was also found within the CACTA-like elements of *C. quinoa* in scaffolds 1686 (position of *Transposase_21* CD 1187046–1187672), 3500 (see above), and 4480 (position of *Transposase_21* CD 7559851–7561239), with similarity between consensus monomers of 97.7–98.8%. A search in the contig-level assembly of the other analyzed *Chenopodium* species revealed a satDNA array with a similar monomer of 171 bp only in the genome of *C. sosnowskyi* but with no connection to CACTA-like elements (Fig. 5B2). The similarity between consensus monomers from the genomes of *C. quinoa* and *C. sosnowskyi* was 74.4%. In all analyzed elements with the additions of tandem repeats satDNA array was close to 3’ end. Fluorescent In Situ Hybridization (FISH) was performed to determine the chromosomal distribution of the newly discovered satDNA family. According to the FISH data, the family is evenly dispersed along the chromosomes of both *C. quinoa* and *C. sosnowskyi* (Fig. 5B3-4).

## Discussion

Application of the short-read genome assembly algorithm followed by mining of CACTA-like transposable elements and their analysis in this study allowed for the determination of several complete elements in the genomes of the *C. album* agg. species. We revealed the structural diversity of CACTA-like elements in the genomes of the studied group of species.

### Divergence of TPases

Estimation of TE divergence based on sequence identity of coding sequences common to all TE of a certain group can be seen as a first step towards exploring TE diversity [37]. In CACTA-like elements, TPase, which catalyzes transposon movement, is a protein sequence that evolved slowly enough to enable alignment of sequences. The phylogenetic relationships of TPases from the genomes of species from different taxonomic groups of angiosperms are summarized in Fig. 1. Although the sequence divergence between TPases is thought to be based more on selective constraints than on divergence time between groups of plants [37], the generated phylogenetic tree is generally in agreement with the trends in the evolution of Spermatophytes and thereby excludes horizontal transfer to a certain extent. Thus, the TPase of the gymnosperm *P. menziesii* was in a distant position to all angiosperm TPases. TPases of *A. trichopoda* were in a basal position, which is consistent with the modern view on systematic positioning of *Amborellales* [25], and di- and monocotyledons were well separated. Therefore, the comparatively isolated position of the TPases of CACTA-like elements of the studied *Caryophyllales* species better support the views of Takhtajan [23] and Cronquist [24], which consider *Caryophyllales* as separately evolving branch of dicotyledons, than the view of the Angiosperm Phylogeny Group [25], which places the *Caryophyllales* at the base of asterids. On the other hand, the position of *H. annuus* at the base of the Clade I (Fig. 1) may favor the APG assumption.

The next outcome from the TPase comparative analysis was that the developed phylogenetic tree clearly emphasizes the presence of two major subtypes of CACTA-like transposons in the genomes of *C. album* agg. species. Interestingly, that the difference between the two *Chenopodium* TPase-based clades exceeds the TPase variability in other Angiosperms (including Di- and Monocotyledons). Previous phylogenetic work has been done on TPases of CACTA elements which have suggested several groups [14]. We determined the relationships of these groups with respect to our phylogenetic tree (data not shown). Although the newly generated ML tree (involving our sequences augmented with sequences from Buchmann et al. [14]) was constructed based on amino acid sequences, the distinction of our *Chenopodium* sequences into two clades was preserved. None of the *tnp2* sequences of Buchmann et al. [14] grouped within the two *Chenopodium* clades, except for *G. max* which was nested within the Clade I. However, Clade I showed only moderate bootstrap support (70). Furthermore, unlike in Fig. 1, *B. vulgaris* appeared as a sister taxon to Clade II of *Chenopodium* sequences which coincide with *Amaranthaceae* taxonomy.

Another important point is that, evidently, in diploid and polyploid *Chenopodium* species the two TPase-different subtypes of CACTA-like elements could coexist in a single genome because both clades shown in Fig. 1 contain the same genotypes. Existence of the two subtypes of CACTA-like elements within one species was recorded in other Angiosperms. Thus, similar results were obtained by Zabala and Vodkin [38] for the genome of *G. max*. The described phenomenon raises the question of how two diverged TPases appear within a single genome. For now, we can only hypothesize that this could be the result of an ancient genome duplication event in the early phase of taxon formation, when two diverged parental TPases were united in one nucleus. Such key events happened several times in seed plant evolution [39]. Alternatively, it is possible that the different rate of change in part of the genomic pool of the CACTA-like elements is due to the inclusion of specific DNA fragments (see below).

### Variation in the full-length CACTA-like elements

Analysis of the full-length CACTA-like elements identified in the genomes of four *Chenopodium* species also revealed variability, and this variability was significantly higher when compared with TPases (S4 and Figs. 2, 3, 5). A full-length *Jozin* element discovered by our group [30] was the first complete CACTA-like element identified in *Caryophyllales*. On one hand, its structure is rather conserved and typical for the CACTA family of transposons [13] with TIRs and subTIRs at the ends and TPase encoding conserved ORF 1 in the middle. But conservation at the DNA level in the remaining internal parts of the CACTA-like elements is low between the explored elements (Fig. 2C). This is consistent with data from assembled *C. quinoa* genome and from other plant systems, such as from CACTA-like elements of *Triticeae* species genomes [11]. This data thus raises the following questions: (i) what are the structural differences in the internal parts of the identified CACTA-like elements of the four *Chenopodium* species; (ii) what causes these differences, and (iii) can structural differences affect the properties of the element? Further analysis revealed the following structural variations within the full elements: (i) appearance of additional CDs within the coding sequences; (ii) presence of captured gene fragments; and (iii) presence of captured (or possibly generated) satDNA arrays.

### Variation in CD architecture

The study of CDA in multi-domain protein families often reveals their evolutionary history and is a common tool in sequence classification [35]. We analyzed four complete CACTA-like elements from our material, 11 from the TREP database, and six from publicly available assembled genomes for the presence of CDs. The four types of CDA were recognized. The most common was Type 1, which was found in the CACTA-like element from the genome of *C. sosnowskyi*. A similar type of CDA was also identified by CDART search in NCBI CDD and by analysis of complete CACTA-like elements from the TREP database in the genomes of species throughout all major clades of dicotyledons, *Poales* of monocotyledons, and the gymnosperm *P. menziesii* (Fig. 3B). It should be noted that four CDs of Type 1 were also present in other CDA types as a component of ORF 1, and their sizes and interpositions were fairly similar in investigated species with the exception of the gymnosperm *P. menziesii* and basal angiosperm *A. trichopoda* (S4). Thus, CDA of Type 1 may be recognized as “basic” for the whole coding sequence of CACTA-like elements of Spermatophytes. Type 2 of CDA where in addition to the four “basic” CDs of ORF 1, a fragment of *Peptidase_C48* CD appeared in ORF 2 (i.e. within the aggregated coding sequence of the element identified by AUGUSTUS) was the rarest. This was found in the genome of *C. pamiricum* and by CDART search in the genome of the legume *T. subterraneum* (Fig. 3B). Peptidase is an ancient CD that is common to archaea, bacteria, and eukaryotes [40]. In particular, the *Peptidase_C48* family occurs in most phyla of the five eukaryote kingdoms. For example, *Peptidase_C48* CDs can be found in *A. thaliana* (O65278) and in human (Q9H4L4) genomes. In Type 3 of CDA, in addition to the four “basic” CDs, a fragment of *Transposase_24* CD appeared within the coding sequence. This CD is known to be present in ORF 2 of CACTA-like elements [41] and was found in the genome of *C. iljinii* and in the genomes of species from *Caryophyllales*, *Brassicales*, *Fabales, Poales*, and the deeply rooted basal angiosperm *A. trichopoda*. Thus, this CD is widely scattered across the phylogenetic tree of Angiosperms*.* *Transposase_24* CD belongs to a family of TPases other than *Transposase_21*. In Type 4 of CDA, all six CDs mentioned above were present within the coding sequence. It is important to emphasize that this type was found exclusively in the genomes of *Caryophyllales*, namely in *C. iljinii* and *C. vulvaria*. Therefore, one or more different CDs may or may not be present in the ORF2, and it can be argued that the ORF2 is open to the intrusion of additional coding sequences. Recently, the phenomenon of ORF2 structural variability was also determined in retrotransposons [42]. Thereby, it is possible to formulate the general principle of the structural stability of ORF1 and wide variability of ORF2 coding regions for plant TEs.

But why does this happen and what leads to the gaining of additional CDs in the coding sequence of CACTA-like elements? To answer this question, the trends concerning CDA dynamics that were already discovered should be considered. Within eukaryotes, over 600 CDs with functions related to nuclear, extracellular, and signaling proteins have been identified. Most CDs are evolutionarily ancient and each domain may fulfill its own function independently or in a concerted manner with its neighbors [32]. Combining CDs in different ways has led to a large number of observed CDAs. It was established that CDs in combination will be favored or not by selection rather than by chance and some combinations appear more frequently than others [43, 44]. Analysis of protein domain pairs showed that pairs of domains that are close neighbors on a protein sequence tend to appear in the same order in different proteins and their relative spatial orientation may also be conserved [43]. Gaining of additional CDs within the coding sequences of CACTA-like elements as we observed is consistent with the abovementioned findings. Thus, a limited number (two) of additional CDs were identified and (if present) they were spaced in the same order in CACTA-like elements from genomes of the investigated species (*Transposase_24* CD followed by a *Peptidase_C48* CD). Even if there was only one additional CD (as in CDA Types 2 and 3), spacing was maintained (S4 and Fig. 3A). Therefore, there is most likely a random process of CD gaining common to multidomain proteins. We do not assume that one CDA type evolved from another, but, based on the scattering of types 2, 3, and 4 along the evolutionary tree of Angiosperms, we supposed that gaining occurs rather stochastically. It should also be noted that in CDD there are more variants of CD combinations, for example the “basic” four CDs of ORF 1 may be combined with *Orbi_VP1* or with *SANT* and even with three or more domains, including *ribosomal_S8e_like* (For example, GenBank: XP_026441742.1, TYK17964.1), but investigation of all CDA variants is beyond the scope of the present research.

It is most likely that the mechanism of the additional CDs gaining in the coding sequences of CACTA-like elements is the same as was identified for multidomain proteins where recombination is regarded as one of the main drivers [32]. While CD gain events are a functional innovation that can contribute to the subsequent evolution of CACTA-like elements, the specific mechanisms should be determined experimentally. Additionally, the captured domains like the captured gene fragments (see below), may serve as “camouflage” to defeat silencing by the host epigenetic machinery, which was suggested to prolong the existence of TEs in the genome [45, 46]. The epigenetic conflict model supposed that capturing of important gene fragment may advantage the TE. Indeed, except for being a part of the CACTA element, *Peptidase_C48* CD was found in the ubiquitin-like-specific protease 1B and 1C genes in the *A. thaliana* genome (O65278, Q8RWN0).

If we further consider our data and return to the divergence of CACTA-like elements in the genomes of related *C. iljinii* and *C. pamiricum* mentioned above (S5), and to the presence of different subtypes of CACTA elements in single genome of *C. iljinii*, it can be assumed that changes in CDA probably may affect the overall structure of the coding sequence and allow faster divergence. The latter assumption is supported by differences in the nucleotide composition of two cloned coding sequences, namely *C. iljinii 1* (TPase Clade II and CDA Type 3) and *C. iljinii 2* (TPase Clade I and CDA Type 4) from one genotype of *C. iljinii* 433–9 (S2 and S6). Pairwise identity of clones was only 51.6%, thus showing that at least two types of CACTA-like elements differing both in TPases and in CDA may coexist in one organism.

### Captured gene fragments and satDNA arrays

The next event that can contribute to variability of the inner parts of CACTA-like elements is unrelated sequence capturing. The capturing, merging, and relocating of host genome sequences is a common feature of DNA transposons [19, 46–49]. The appropriate mechanism was described by Rubin and Levy [50]. We found several cases of genes captured by CACTA-like elements in the genomes of the analyzed species. The first case was the presence of the *Neprosin* gene fragments in the complete CACTA-like element from the genome of *C. vulvaria* (Fig. 5A). *Neprosin* is connected with special enzymes of carnivorous plants and, as is known, *Caryophyllales* includes many carnivorous species in the families *Droseraceae, Nepenthaceae, Drosophyllaceae, Dioncophyllaceae* [51]. Thus, *Chenopodium* species could have inherited this gene from a common ancestor.

Another gene fragment captured by CACTA-like elements was *Apetala 2* (AP2). The fragment of 80 bp was found within CACTA-like elements in the genomes of *C. album, C. acerifolium,* and *C. opulifolium* upstream from TAD (Fig. 5A). Here, it is worth mentioning two important points. First, the similar positioning of AP2 fragment in CACTA elements from three different tetra- and hexaploid genomes with a common haplome “B” may support the model when gene fragments provide “camouflage” for TEs, and they will exist within the genome for longer periods of time than free TEs [45, 46]. Second, AP2 is a gene and a member of a large family of transcription factors, the AP2/EREBP family. In *A. thaliana*, AP2 plays a role in the ABC model of flower development [52] and is required for the transition of an inflorescence meristem into a floral meristem [53]. While the capture of *Neprosin* gene fragments most probably is of low evolutionary significance for *Chenopodium* species, the capture and transfer of *AP2* fragments (one of the key genes for flower formation) can have long-term evolutionary consequences. It is quite possible that exon shuffling may occur with the subsequent creation of novel gene products due to the new placement of two previously unrelated exons through transposition [54]. Thus, DNA transposon-mediated transduction can be recognized as a significant mechanism that can potentially contribute to flowering plant evolution [16].

Perhaps the largest increase in the length of CACTA-like elements was due to the capture of satDNA arrays. We found one case of satDNA capturing (Fig. 5B). In the assembled genome of *C. quinoa*, an array of 32.5 copies with a consensus monomer of 171 bp was found inside a CACTA-like element with complete ends. It should be noted that this satDNA family was newly identified and has multiple arrays in the genome of *C. quinoa* that are very often located inside or nearby CACTA-like elements. At the same time, we found no indication that the arrays were generated by CACTA-like elements as we previously observed for the major satDNA family of the *Chenopodium* genome [30, 55]. Scanning of the assembled to contig-level genomes of other explored *Chenopodium* species revealed a similar satDNA family only in the genome of the early diverged allotetraploid *C. sosnowskyi* but with no connection to any CACTA-like elements. The two species were separated some 11 mya [22] and it is possible that the ancient satDNA family was significantly amplified in *C. quinoa* genome*.* This was most likely accompanied by its capturing by CACTA-like elements and subsequent spread across the genome. In this scenario, CACTA-like elements can be viewed as causal agents for satDNA landscape change.

The last point to be mentioned, concerns positions of additions in CACTA elements. When we compared positions of the captured gene fragments and satDNA arrays we noted that the former were found in the 5’ region upstream to ORF 1 and the latter in the 3’ region. Thus, there are regions of the element that seem to be prone to capturing extraneous DNA.

## Conclusions

This study showed that while preserving the basic structure, CACTA-like elements of the *Chenopodium* genome jointly evolved as a member of *Caryophyllales*, which appears to be a separate branch of dicotyledons. Besides branch specificity, the significantly different subtypes of elements could coexist within one organism. CACTA elements sometimes carry coding and non-coding unrelated DNA fragments in the regions that seems to be prone for capturing extraneous DNA. Supplemental DNA fragments may putatively have different effects on both transformation of the element itself and on the whole genome transformation. Database analyses showed that the identified variants in CACTA-like element structure are widely scattered across the phylogenetic tree of Angiosperms.

At the same time many subsequent specific questions were raised for future investigation. For example, how do the properties of an element with gained additional CDs change? Can additional CDs inhibit each other and, therefore, are such types of elements less common (although they appear repeatedly in different branches of Angiosperms) or is CD gaining neutral? If TEs can capture genes (from the same plant) and are able to relocate them to different places in the genome, can these elements act as vectors to shuttle specific DNA (genes) between species? The answers to these questions could contribute significantly to our knowledge regarding the mechanisms of genome evolution.

## Methods

### Plant material, DNA extraction, library preparation, and Illumina sequencing

For the preparation of the DNA libraries, 22 species of *C. album* agg. Were used (S1). DNA was extracted from fresh leaves using a DNeasy Plant Mini Kit (Qiagen) according to the manufacturer’s instructions. For all analyzed accessions, the DNA ploidy level was assessed by flow cytometry as described previously [56]. Fifty-nine accessions were used for library preparation and NGS (S1). The details of library preparation and Illumina sequencing have been described previously [57]. The Illumina data have been deposited in the NCBI Sequence Read Archive as BioProject PRJNA807097.

### Assembly of Illumina reads to contigs, search for CACTA elements, and data processing

Geneious Prime software version 2019.2.1 (https://www.geneious.com) was used [58] for the processing of Illumina NGS data and the identification of the localization of CACTA-like elements in the genomes of all investigated species. The advantage of this assembler is that it produces large contigs. De novo assembly was performed with medium-low sensitivity, which is the best option for large numbers (e.g., 100,000 or more) of Illumina reads.

The position of TPase in the contig-level assembly was revealed using DANTE, a component of the RepeatExplorer pipeline [36]. Protein domain filter parameters were TPase, Minimum identity 0.35, Minimum similarity 0.45, Minimum alignment length 0.8, Interruptions [frameshifts + stop codons] 0, Maximal length proportion 1.2. Full-length sequences of about 630 bp (complete *tnp2* domain) were selected manually and remnant sequences were excluded. The described algorithms were applied to the genomes of each of the analyzed species separately.

As a first step in understanding the natural variability of CACTA-like elements, the phylogenetic analysis of TPases was used. The TPase sequences of *Chenopodium* species were analyzed in a phylogenetic context involving representative Angiosperm lineages. Representative Angiosperm lineages were selected based on the APG IV taxonomic system of flowering plants [25]. TPase sequences were retrieved from BLAST search, CDART and TREP databases, and specific assembled genomes using DANTE pipeline search for TPases of CACTA-like elements. Sixty-eight sequences (S2) were aligned using MAFFT implemented in Geneious and the alignment was refined manually. The phylogenetic relationships of the TPase sequences were analyzed by computing a gene tree using maximum-likelihood (ML) analyses with MEGA X version 10.0.05 [59]. Tamura 3-parameter model [60] with gamma distribution (5 categories) and a proportion of invariant sites were determined using Bayesian information criterion as best fitting the dataset. The tree was rooted with *Gymnospermae* species of *Pseudotsuga menziesii*. The nucleotide alignment consisted of 651 positions and all were used for the analysis. Extensive subtreepruning-regrafting, a very strong branch swap filter, and 1000 bootstrap replicates were used along with the specific model of molecular evolution for the dataset.

For analysis of a whole CACTA-like *Jozin* transposon, complete or almost complete (visible subTIRs) ended elements were selected from contig-level assembled *Chenopodium* genomes. Such elements types were identified with the YASS genomic similarity tool (http://bioinfo.lifl.fr/yass/yass.php) [61], which shows mirrored zones of tandem repeats at the ends of an element (Fig. 2A). Additionally, for comparative analysis we recovered CACTA-like elements with the DANTE and YASS programs (firstly, the full TPases were positioned and then TIRs/subTIRs were searched in the surrounding areas) from publicly available genomic sequence data of the following genomic sequencing initiatives: *A. trichopoda* (At_29896_AVHE01), *B. vulgaris* (RefBeet-1.2.2), *C. quinoa* (*Chenopodium quinoa* v1.0 (Quinoa)), *M. ashei* (NCBI PCNC01), and *P. menziesii* (Psme.1_0). In addition, complete CACTA-like elements were also selected from the TREP database [33].

The Hidden Markov Model-based AUGUSTUS program [62] was used to determine the coding sequence within each analyzed CACTA-like element (http://bioinf.uni-greifswald.de/augustus/submission). This is a tool for finding protein-coding genes in genomic sequences in ab initio mode. The training for *A. thaliana* as the most explored genome of dicots was used. Within the coding protein sequence of a whole element, determined by AUGUSTUS conservative domains were distinguished with the *Batch* Web CD-search tool (https://www.ncbi.nlm.nih.gov/ Structure/bwrpsb/bwrpsb.cgi?). Similar CDAs were found by NCBI CDART in a CDD (https://www.ncbi.nlm.nih.gov/Structure/lexington/lexington.cgi) [35]. Captured gene fragments were determined using an NCBI Conserved Domain search (https://www.ncbi.nlm.nih.gov/Structure/cdd/wrpsb.cgi). Captured satDNA arrays were determined with YASS (Fig. 5B1). All determined complete CACTA elements were submitted to the TREP database.

### Experimental validation of computationally identified CDA structures

Primer pairs were designed for PCR identification of the physical counterparts of computer-generated assembled CDs (S7). The task was to confirm the in silico detected CDA types. As a template, we used total DNA of *C. iljinii* (accession 433–9) as a species in the genome of which two CDA types have been found. For primer design we used contigs *C. iljinii 1* and *C. iljinii 2* which according to assembly results were representing both CDA types (S2 and S4). The fragments of clones were amplified by a genome-walking strategy. PCR was performed in 25-μl reactions containing 1x Q5® High-Fidelity Master Mix (NEB), each primer at 0.2 mM, and 5 ng of genomic DNA. The cycling conditions were as follows: 2 min at 98 °C followed by 35 cycles of 98 °C for 20 s, the sequence-specific annealing temperature (60–65 °C) for 30 s, 72 °C for 3.5 min, and a final extension at 72 °C for 15 min. The PCR results were visualized in a 1% agarose gel. For cloning, the PCR products of clusters were excised from the gels using a PureLink™ Quick Gel Extraction and PCR Purification Combo Kit (Invitrogen), followed by addition of an adenine residue to the 3’ end of both strands of DNA (20-μl reactions containing PCR buffer, 0.5 U *Taq* DNA polymerase [Top-Bio], 0.2 mM of dATP, 1.5 mM MgCl_2_, and purified PCR product; incubation for 20 min at 72 °C). The PCR product was then cloned using a TOPO-TA cloning kit (Invitrogen). The clones were linearized by NotI restriction enzyme (NEB) and sequenced at Eurofins Genomics (Konstanz, Germany) from both sides. From the obtained sequences of the Tp24-X1 and 68TAD-X1 clones, the following new primers were designed: Tp24-X1_F2 and R2 (PCR performed as above with annealing temperature 60 °C and elongation 2.5 min), F3 and R3 (61°C, 1.5 min), 68TAD-X1 F5 and R5 (57°C, 2.5 min), and F6 and R6 (59°C, 1.5 min). The remaining clones were sequenced (S6). The aligned sequences have GenBank Accessions ## MZ325224, MZ325225.

### FISH procedure

FISH was performed to detect the chromosomal distribution of the satDNA family found captured by CACTA-like elements in genome of *C. quinoa* and found separately in the genome of *C. sosnowskyi*. Root tips were pretreated in 0.002 M 8-hydroxyquinoline for 3 h in the dark and fixed in 3:1 (v/v) 100% ethanol:acetic acid. The fixed root meristems were thoroughly washed in water and enzyme buffer (10 mM citrate buffer at pH 4.6) and partially digested in 0.3% (w/v) cytohelicase, pectolyase, and cellulase (Sigma, St. Louis, MS, USA) at 37 °C for 3 h followed by several washes in water. The material in a water drop was carefully transferred to a grease-free microscope slide and cells were spread as previously described [55].

Primers were designed based on the consensus sequences (S7) to prepare FISH probes for chromosomal localization of the newly detected satDNA family. PCR was performed in 25-μl reactions containing 1x TopBio Plain PP Master Mix (TopBio), each primer at 0.2 mM, and 5 ng of genomic DNA. The cycling conditions were as follows: 5 min at 95 °C, 35 cycles at 95 °C for 30 s, the sequence-specific annealing temperature (55 °C) for 30 s, 72 °C for 1.5 min, and a final extension at 72 °C for 15 min. The PCR results were visualized in a 1% agarose gel, where a typical ladder structure of a tandem array was obtained (Fig. 5B2). FISH experiments were performed with the PCR product as a probe that was labeled with Cy3 (Amersham, Amersham, UK) according to a standard oligo labeling protocol. FISH was performed in a ThermoBrite programmable temperature-controlled slide processing system at 63 °C for 3 h. The slides were stained with DAPI, mounted in antifade mountant (Vector Laboratories, Peterborough, UK), and examined and photographed on a Zeiss Axio Imager.Z2 microscope system.

## Supplementary Information

**Additional file 1 **(1) Phylogenetic tree of the *Chenopodium album* agg. (2) Accessions and geographic origins of investigated *Chenopodium album* agg. species.

**Additional file 2.** Table of analyzed contigs/scaffolds containing fragments or CACTA-like elements.

**Additional file 3 **Sequences of CACTA-like element *Jozin* from genomes of *C. iljinii* and *C. vulvaria.*

**Additional file 4.** Table of Conserved Domains and Conserved Domain Architectures.

**Additional file 5.** Pair wise distances between ORF 1 of the four Chenopodium on protein level.

**Additional file 6 **Comparative analysis of contigs and clones form *Chenopodium iljinii* genome.

**Additional file 7.** Primer pairs used in this study.

## Data Availability

The Illumina data have been deposited in the NCBI Sequence Read Archive as BioProject PRJNA807097. The physical counterparts of computer-generated sequences have GenBank Accessions numbers MZ325224, MZ325225. Four complete CACTA elements were submitted to TREP database. All annotations files and generated output data sets corresponding to number of contigs and/or reads mapping putative CACTA-like elements and satDNA families are included in this published article (and its Supplemental information files).

## Abbreviations

FISH: Fluorescent in situ hybridization
ITAP: Inter-transposon amplified polymorphism
NGS: Next-generation sequencing
satDNA: Satellite DNA
TEs: Transposable elements
TIRs: Terminal inverted repeats
subTIRs: Subterminal repeats
TPase: Transposase
