# Supplementary material for: The structural diversity of CACTA transposons in genomes of Chenopodium (Amaranthaceae, Caryophyllales) species: specific traits and comparison with the similar elements of angiosperms
Source: Mob DNA. 2022 Apr 4;13:8. doi: 10.1186/s13100-022-00265-3 (PMC8978399; doi:10.1186/s13100-022-00265-3)
Supplement: Supplementary file 1 — Additional file 1 (1) Phylogenetic tree of the Chenopodium album agg. (2) Accessions and geographic origins of investigated Chenopodium album agg. species. [file 13100_2022_265_MOESM1_ESM.docx]

**S1.** **(1) Phylogenetic tree of the *Chenopodium album* aggregate**


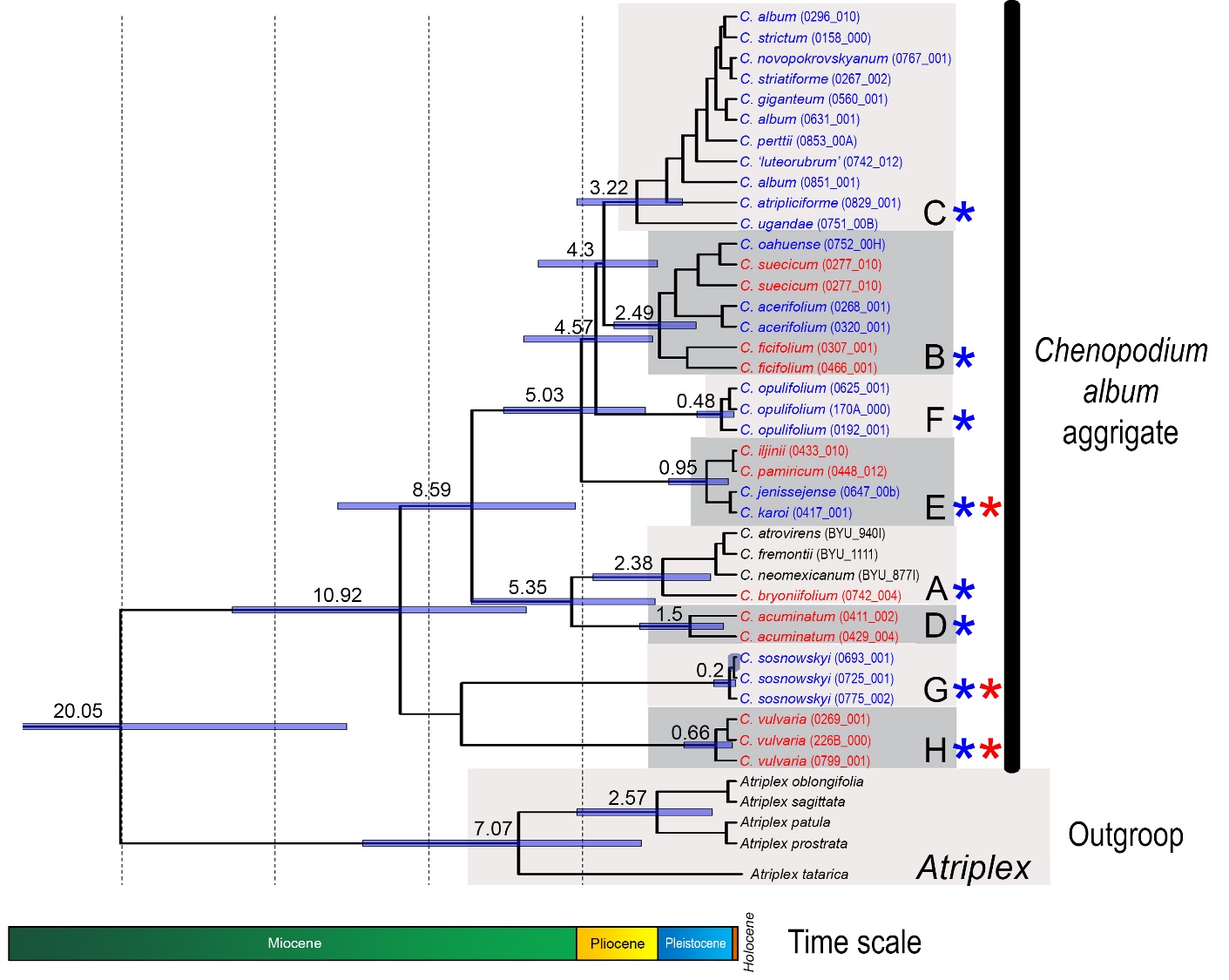


Phylogenetic tree calculated using Bayesian inference within the *C. album* aggregate estimated based on the concatenated dataset of three chloroplast DNA spacers (adapted from [22]). Major evolutionary lineages are marked by grey rectangles. The numbers above branches correspond to the ages of the particular clades (in millions of years) as inferred by the analysis in BEAST2. Diploid species are shown in red. Polyploid species are shown in blue. American species are shown in black (for comparison, not analyzed). The schematic stratigraphic time scale (Miocene - Holocene) is shown at the bottom of the figure. Clades where comparative analysis of TPase was made are marked with blue asterisks. Clades where complete CACTA elements were determined are marked with red asterisks.

**(2) Accessions and geographic origins of *Chenopodium album* aggregate species**

| No | Species | Accession number | Genome compos. | Locality | Coordinates |
| --- | --- | --- | --- | --- | --- |
| 1 | *C. acerifolium* | 268-3 | B+D | Latvia, Strenci | N 57.619489, E 25.701168 |
| 2 |  | 316-1 |  | Russia, Velsk | N 61.066704, E 42.095002 |
| 4 | *C. acuminatum* | 429-3 | D | China, Xinjiang, Altaj, Burqin | N 47.815500, E 87.080028 |
| 5 |  | 429-4 |  | China, Xinjiang, Altaj, Burqin | N 47.815500, E 87.080028 |
| 6 |  | 987-10 |  | Russia, Altai, Travnoe | N 52.52817, E 81.77047 |
| 7 | *C. album* | 291-1 | B+C+D | Czech Republic, Hrádek | N 48.781583, E 16.261528 |
| 10 |  | 423-3 |  | Cjina, Xinjiang, Altai, Kanas | N 48.675917, E 87.026722 |
| 11 |  | 522-1 |  | Russia, Tyumenskaya Region, Purpe | N 64.486083, E 76.714333 |
| 12 |  | 620-3 |  | Portugal, Torreira | N 40.764400, E 8.700631 |
| 13 | *C. atripliciforme* | 829-2 | C+D | Tajikistan, Gorno-Badakhshan | N 37.2975, E 71.505833 |
| 14 | *C. bryoniifolium* | 742-6 | A | Russia, Primorski Krai, Nakhodka | N 42.88775, E 132.722361 |
| 15 | *C. ficifolium* | 276-5 | B | Czech Republic, Nový Bydžov | N 50.234361, E 15.428778 |
| 16 |  | 330-2 |  | Czech Republic, Slatina | N 50.226389, E 14.210528 |
| 17 |  | 458-2 |  | China, Xinjiang, Tumuxiukezhen | N 41.534139, E 79.753778 |
| 18 | *C. frutescens* | 988-1 | unknown | Russia, Altai, Kosh-Agach, Ortolyk | N 50.00468, E 88.34724 |
| 19 | *C. iljinii* | 433-9 | E | China, Xinjiang, Altaj, Hoboksar | N 46.541472, E 85.358083 |
| 20 |  | 441-3 |  | China, Xinjiang, Hoxud, Bo Si'amu | N 42.474417, E 86.877806 |
| 21 |  | 453-8 |  | China, Xinjiang, Kuqa | N 42.278889, E 83.266889 |
| 22 |  | 461-1 |  | China, Xinjiang, Tumuxiukezhen | N 41.667306, E 79.693528 |
| 23 | *C. jenissejense* | 640 | B+E | Russia, Verkhnekolymsky ra. | N 64.646833, E 151.640306 |
| 24 | *C. karoi* | 417-4 | B+E | China, Xinjiang, Altai, Kelancun | N 47.926556, E 88.136917 |
| 25 |  | 418-2 |  | China, Xinjiang, Altai, Kelancun | N 47.945306, E 88.160222 |
| 26 |  | 460-1 |  | China, Xinjiang, Tumuxiukezhen | N 41.667306, E 79.693528 |
| 27 |  | 460-6 |  | China, Xinjiang, Tumuxiukezhen | N 41.667306, E 79.693528 |
| 28 | *C. luteorubrum* | 742-13 | A+C+D | Russia, Primorski Krai, Nakhodka | N 42.88775, E 132.722361 |
| 29 |  | 745-2 |  | Russia, Irkutskaya Region, vicinity of the city of Irkutsks | N 52.20, E 104.1 |
| 30 | *C. novopokrovskyanum* | 463-3 | C+D | China, Xinjiang, Tumuxiukezhen | N 41.667306, E 79.693528 |
| 31 |  | 487-7 |  | Russia, Tuva | N 51.399722, E 94.760000 |
| 32 |  | 731-1 |  | Iran, West Azerbaijan, Poldasht | N 39.056944, E 45.430556 |
| 33 | *C. opulifolium* | 696-4 | B+C+F | Iran, Kurdistan, Marivan | N 35.498461 , E 46.166946 |
| 34 |  | 696-6 |  | Iran, Kurdistan, Marivan | N 35.498461, E 46.166946 |
| 35 |  | 1174-1 |  | Ukraine |  |
| 36 | *C. pamiricum* | 177 | E | Russia, Altai, Kosh-Agach | N 50.055278, E 88.708611 |
| 37 |  | 830-3C |  | Tajikistan, Gorno-Badakhshan, Murghob | N 37.821667, E 73.566667 |
| 38 |  | 989-1 |  | Russia, Altai, Kosh-Agach, Chegan-Uzun | N 50.069652, E 88.411782 |
| 39 |  | 990-1 |  | Russia, Altai, Kosh-Agach, Chegan-Uzun | N 50.00403, E 88.23078 |
| 40 | *C. probstii* | 399-2 | B+C+D | China, Xinjiang, Tian Shan, Zhongliangcun | N 43.513278, E 87.346861 |
| 41 |  | 450-5 |  | China, Xinjiang, Fanxiu Bridge | N 43.084694, E 84.832361 |
| 42 |  | 467-1 |  | China, Xinjiang, Bogda Shan, Fukang | N 43.900333, E 88.113222 |
| 43 | *C. sosnowskyi* | 775-2 | A+G | Iran, East Azerbaijan, Kaleybar | N 38.917790, E 47.030593 |
| 44 |  | 788-3 |  | Iran, west Azerbaijan, Siah Cheshmeh | N 39.065972, E 44.386170 |
| 45 | *C. striatiforme* | 267-2 | C+D | Latvia, Strenci | N 57.619489, E 25.701168 |
| 57 |  | 331-1 |  | Czech Republic, Mělník | N 50.349528, E 14.497444 |
| 46 | *C. strictum* | 380-1 | C+D | Czech Republic, Prague | N 50.115964, E 14.433326 |
| 47 |  | 380-5 |  | Czech Republic, Prague | N 50.115964, E 14.433326 |
| 48 |  | 438-2 |  | China, Xinjiang, Turpan, Toksun | N 42.486722, E 88.528306 |
| 49 |  | 456-1 |  | China, Xinjiang, Tumuxiukezhen | N 41.534139, E 79.753778 |
| 50 |  | 478-1 |  | Russia, Volgograd area | N 49.100000, E 46.119722 |
| 51 |  | 480-4 |  | Russia, Samara area, Zhigulevsk | N 53.416667, E 49.533333 |
| 52 |  | 481-1 |  | Russia, Saratov area, Balakovo | N 51.900000, E 47.633333 |
| 53 | *C. suecicum* | 328-10 | B | Czech Republic, Švermov | N 50.176806, E 14.105472 |
| 54 |  | 992-10 |  | Sweden, Örnsköldsvik | N 63.292, E 18.706 |
| 55 | *C. ucrainicum* | 1030-1 | B | Ukraine, Kyiv region | NA |
| 56 |  | 972-1 |  | Ukraine, Kyiv region | NA |
| 57 | *C. ugandae* | 751-1 | B+C | Rwanda, seed obtained from IPK Gatersleben | NA |
| 58 | *C. vulvaria* | 719-1 | H | Iran, Isfahan | N 32.615131, E 51.659462 |
| 59 |  | 771-1 |  | Iran, Ardabil, Meshgin Shahr | N 38.405556, E 47.694722 |
