# Supplementary material for: The structural diversity of CACTA transposons in genomes of Chenopodium (Amaranthaceae, Caryophyllales) species: specific traits and comparison with the similar elements of angiosperms
Source: Mob DNA. 2022 Apr 4;13:8. doi: 10.1186/s13100-022-00265-3 (PMC8978399; doi:10.1186/s13100-022-00265-3)
Supplement: Supplementary file 3 — Additional file 3 Sequences of CACTA-like element Jozin from genomes of C. iljinii and C. vulvaria. [file 13100_2022_265_MOESM3_ESM.docx]

S3. Sequences of CACTA-like element *Jozin* from genomes of *C. iljinii* and *C. vulvaria*

>Cilj_Jozin_consensus1_Chenopodium iljinii; DNA-transposon, TIR, CACTA; complete element

CACTAGTAGAAAAATGTACATTTGCGTCGCCATATTTGCGTCACTCATTCCAAAGAACGACGCAAATGTTTGTTTTTACCATCCTTAAAAGTTATTTGCGTCACACTTTATTCAAGAAGCGATGCAAAAGTCAGATGTATTTCAGCTTTCTATTATTTGCGTCGCTCTTATTTATTAAAGCGACGCATATAATAATCTTATTTGCGTCACTTATTTATTGACAAGTGACGCAAATGAGTGAGTGAATAAATTGCGTCACGCTTTTAATAAATGAGCGACGCATATGAAAATGTACATCATTATACTTTTTGTTATTTGCGTCGCATTTTAATAATTGAGTGACGCAAATAACTCAATTGCATTTCAGAATTTTTGACCTTAGCTTTTCACGCCTACCTTTCACTAAACCAAAAGAAAAAAAACAAGAGCATCGCTACTCCATTGAATTTCTACAGCAAAACACCCACAAATTCGACCCTAAATCCAACAAATTCGACCAAAAAACTCCGATTTCCCAGCAAATTCAACATCTAAAATCGCAATCAAGTTCAAGACCCATAATCGGCAGTAAATTCGAGCTATAATCCCCCAATTTCGAGCAAAAATTCAGACCAAAAACCCTAAGAAATTCGAACCCATAAACCCCAGAAAAATTGAGCAATTCCGACCATTTCGAGCAAAAAATCTACTCAAAACCCTAGGAATTCGAACCCTAGAAACCCCAAAAAATTCAATTAAAAATCCTTGCCTCATATTCGAGCAATGACTGAAATTTTCGACTTAATTTGTGAAGTCATTTTTGGATTAATTTTGCCCCCAAAAAACCCGAGGAATTTATAGCAAATTACAGAAGTGTGGCAGTTTCGACTGAATTTGTGAAGTCGATTTCACCTAAGGTAAGATTGCTTCTATTTATGTTCACTCTTTTCATGGTAGTTAGAATGTAGCATCTGACTTGAGCAATGACTTAGTTTAATTTCAGGTTTAGTGTTAGTTCATTAATTAGAAAATATATGGCAGATTTAGTTGAATATATATAATTTCAGTCATTGAATATATGGCAGATTTAGTTGAATCAACTATGTTGGTTTAATATATGTAATTTTATATGGCAGATTTCTTAATTATGCGGAAGTGAATTCCTCAAGTTGACTGAGTTTCTGGTTGTGGACAAACGATTAAGGCGGTAGTTCACATACGAGATTGTCTTCACTTGTGGTATATATACCTGAACCATTCTTTGCAATATTAACTTATTTTTTGTGGTTGGTATAATGATTGTGAATTGTATGAGATTTTTAATTGTGATATGATTGATTGGAATTGCAATAATACTTGCATCATTGGCTTAGATACCCGTTCAGGGCTTACTCATTAGGAGTAAGTCTTGAATAGTCCCCGGTTTGGGACTGTTCTTGAATGTTCTATCTCATTTAGGTGATAACTACTTGAACTGTTTTTGGGTCTAAGAAGATCATGGCTCACTATTTCCCGATCGTTCTCTGGGTACATATGTAGATAGACGATCATCTATATTGTGTGCTTGGAGAACTATAGGGAAATGCTGCCGGATTTTCTTTAGAACCAAACTTTACAAGTAGTTATTAGTGAGATAGGACATTCAAGAACGGGTTAGGTTGGAGTGATAAGGACCTTGTTGGATCATGTCATTGCATTTACATGAGATAAACTTTGAATGAAAATGTTTGATGCCTTGTATTCTTGTTTATGGTTAAGGAACTATATATGTTAGAAAATATACACATTATCTTTAAATTAATTTTTGTTATTCAGTGATTGAAAATGGATCGAAGTTGGATGTATGGTAAACGAAACTGTACAAGATTTTTACGAGGAATTGAAGAGTTTAGCAATGTCGCCTTAAACTACCAAACAGAGAATAAATCAAAGATTATTCTTTGTCCTTGTTGTGACTGTAATAATTCGAGGGGGTATCGTGATATAGATGATATTAAAGATCATCTAATTCGTCGTGGGTTTAAAGAAAACTACACGAGGTGGACGTGGCATGGTGAGAGCATATATAAAGAGGCTAGTTCTAGTTATTGCCCAAGGGAAGATGAGAATCATTGTGATGATGATAATGAACACCAAGGTGAGGATATCGATACTGCTGGTATTGGTGTTGAAAGTGAGAGTGAAGTGGAGAAGGACAGGATAGATGAAATGATGCATGATGTTGAAGACCATTTCACAGAGTGTCCTCAGACATATGATAGTATTTTGAGAGCTGCAGAAACACCGTTATATCCTGGTTGTACAAAATTCACTAAACTTGGTGCTATTATGAAGTTATTCAACTTAAAGGCGAGCAATTGTTGGACCGATAAGGGTTTTACGCAGTTGTTGGAAGCCTTAGTAGAAATGTTTCCCGAAGGGAATGAACTTCCTAACTCCACCTATGAGGCCAAGAAACTTATGTGCCCTTTAGGTATGGAGTATGTGAAGATACATGCTTGTCCCAATGATTGTGTGTTGTATCGAAATGAGTATGCTGATTTGCATGAGTGTCCAAGGTGTGGAGCTTCTCGTTACAAGATGAATGATACTGGTGAGTTGTGTAAGAAAGGGTCTCCAACTAAGGTATTGTGGTATCTTCCAATTATACCGAGATTTAGGCGACTTTTCACAGATGAAAAAAATGCAAAATTATTGAGGTGGCATGCTGATGGGAGGAAGAAAGATGGGTTAATGAGGCATCCGGCTGATTCCCCGCAATGGAGGAACATTGATCGAAAGTTCAAGGACTTTGGTCAAGAAGATCGAAATCTTAGGCTTGGTCTTAGTACAGATGGAATGAACCCATTTGGGACACTTAGTACCCAATATAGCACTTGGCCGGTTCTCCTAACTATCTACAATTTGCCTCCTTGGTTATGCATGAAGTCTAGATACATTATGTTGTCCCTTCTAATATCTGGGCCTAAACAACCTGGAAATGACATTGATGTGTATCTAGCGCCTCTCATTGAAGATTTGAAATTGTTGTGGAATGAAGGTGTCCAAATGTTTGATGCATATAGTAAAACCAATTTCACTTTACGTGCCATGATTTTTTGTACGATAAATGACTTCCCTGCTTATGGGAATTTGTCGGGGTACACTGTGAAGGGAACAACTCCTTGCCCCATCTGTGAAGATGATTTGGAGGCATTACGCCTAGACAATTGTGGCAAGCATGTATACATGGATAATCGCAGACATCTTCCTGAAGACCACCCTTTTCGAAAGAATAAGGATGCATTTAATGGAAAAGTGGAGTTGAGAGAAGCCCGTGGCCCTTTACGTGCAAGTGAGGTTTATCAACGGGTCAAAGACATTGAGAATGAGTTTGGTAAGCCTTACAAAAGCAAATCAAATGGGGGTTACAAGAAGAAGTCTGAGCTATGGTCTCTTCCATATTGGAGACATTTGGAAGTTAGACATTGTCTAGATGTAATGCATATTGAGAAAAATGTTTGTGATGCCATTGTGGGAACTTTATTAAATATGCCAGGAAAGACAAAGGATGGAGTTAAAGTAAGAAAAGACATGGCTGCTATGGGTCGTTCAGAGTTGGCACCCGAATCTCGAGGAAAACGCTGGTATCTTCCCCCAGCCTGCTTCACCTTGTCTAAAAAGGAAAAAACTAGCTTCTGTGAGTCATTGCATGGTTTAAAGGTCCCGGCTGGATATTCTTCTAATTTTCGTAGACTTGTGTCGATGTCTGACTTGAAATTGGGTGGCATGAAATCTCATGATTGTCATGTCTTGATGCAACAATTATTACCAGTTGCAATTCGAGGAATTTTGCCACCTCAAGTGAGGTATACGATTACAAGATTATGTGTCTTTTTTAACACTATCTGTAGCAAGGTCATAAATCCAAGTATTTTAGATGACTTGCAAGCAGATATACTTGAGACAATGTGTCGATTTGAAATGTATTTTCCCCCATCTTTTTTTGACGTGATGCCTCATTTGGTTATTCATCTTGTACGTGAAATTAAACTTTGTGGACCAGTGTGTATGAGATACATGTATCCTTTTGAACGAGAAATGGGTGACTTAAAGGGAAAAGTCATGAATCCGGCCAAACCTGAAGCTAGTATTGTACAGCGAACAGTTGCTGAGGAAGTGGCAGCATGGGTTGCTCAATATCTTGCACGTTCACATAAAATTGGGTTGCCAAAGTCTCGACACGATGGGAGGCTCGGAGGTCAAGGTACTATTGGTAGGAAAAGGATATCCATGGGCTTTGAAATGAAAAATAAGGCCGAGCTTTTTGTGTTGCAAAATCTTAGTGAAGTTCATCCTTACTTGGACGAGCACATGATTTTTCTTAAAAATAAATATCCTTCCAAAAGTGATCTTCAGCTGATAAAGGAGCATAATTGTTCATTCGTTACATGGTTCAAGGAACGAGTGATGTCCCAGCTGTCCACCACACCTAACGATATATCTGACACATTGAGATGGTTGGCATATGGTGCTAAATGTCAAGTCATTTCATATGAGGGATACGACATCAATGGGTATTCTTTTTACACTAGCCAACAAGATGACAAATCAACAATGCAAAATAGTGGTGTTAAAGTAATAGGTTTGTCATCTGAGTATGTTAGTGCACATGATAAAACACTTGTGGATAAGAAGAAATTTTATTATGGAATCATTGAAGAAATAATAGAGCTGGACTATGTTGATTTCAAGATTCCTCTATTCCAATGTAAGTGGGCTGATAGTAGTCGTGGTGTAAAAAAAGATGAACAAGGGAACTTGACCCTTGTGAATCTTGGTCGACGAGGGCATCTAGCTGATCCATTTATATTAGCATCACAAGCAAAGCAAGTGTTTTACATGGCTGACCCAGCTGATTGTAAATGGTCAGTTGTATTAGAAGGTAAAAGAAGGATACTTGGCATTGAAGATGTGGTGGACGAGGAAGAATATGATGAGCAATTTAATGAGTCACCACCTTCCGTTTGGAGCATCCCTCCAATAGTCGATGATTTTGACACAACGTTGAAACGTAAAGATCATAATGAAGGATTTTATGTCGCAAAAGAGAAGAATGAAGTAGGTATGAATTCTGTGATTTTTTCCAATATGTGTTACTAATTGCATAAATTGCTTATTATTGTTATGTGTACAAGTTTATAATTTATAAATGATACAAATCGATGTCTTAATTGATTTATGTCTTTTTTTTTCAGGTAAGTAATAATGGAGGATGAGGATATGCGTAATCGTTCCATGTCTTCGCAAGGTGAGGAAGATGAGCCACACAATCAATCACAATCACAGCAGCAAACAACCGACTCTCAAAAGAAGAAAAAGAAGCCAAGAGGTCCTTCAAAGGGCCTGAAATCCATGCCTGGGGTTCCTAGAGTGCTTGAATGGGATGAATTGTGTCGACCCATTGGAAAGTGGGCAAAAGCATACAAAATTCATCTTGGTGAAATAAGCCGTGCAAAAGTGTCTATATTGTATAAAGATTGGAATCAAGTTCCACAAGGAATAAAAGACACTTTGTGGGAAGATGTTAAGGTAATATTCACTATTAAATGTTAAATGTTATACTATTATATGATCATTTTGAAATTATTTGAATATGAGACTTCCTTTTTTTTATGTAGAGAGAGTTTCAAATCGAAGAAGATGAAAACAAGAAAAAAAAGGTCCTACGCACTTGTGATAAGTGCTGGAGAGATTTTAAAACAAAATTGGTCAGTGGTTGGATCACATGTACAAGGAATATGCCGAAGGAGAAAAGAATGTCGTATGTACTCTATGATTTCATATCTGAAGATATGTGGAAAACATTTGTGGAGGAGCATAGTACAGATGATTTTAAGGTTTGTGTGTGTATTAAAATCAATGAGCATGCATGTTATTAATACTTTGTTAACGGATTCTTATGTACTATTCTCCATTTATGCTTGTGAAAATAGGAAATCAGTGAGAAGGCAAGACAAAGCCAGTCTTTCAACGAATACCCTCACCATTTAGGAGCCAAATCATATGGTGAAATGAATACTGTTTGGCGTAGAAAAGGGTATATTCCCACATCATCTTCAGCATCCAGTACTTCATCTTGTTCTTCGGTTGTGTCTAGTTTGCCGGATAGGACATATGCTTGGCTTCTAGCAAGATCAGTGGAAGATGATAAGGGAAATCCGTATTTGCCGGATGAGAAAACAAGAGAGGTGAAAGAATCCATTGTAAGTATATAAAAATAAGTCTATTTCAATTTTGAATATATTTTGTTAAGTTTATGTATTGCAACGAAAGTTTACTAACTTGAAGTCTTTGTGTAGGATAATTGGAGAAAGCAACAAGCTGATGGGAAATTTGTTCCTAATAGGCATGATGATATCTTATCTCGTGCCCTTGGGAAAAAAGATCGTAACGGTCGGGCAATAGCATTTGGCAGTGGAATTGGCATTAAAGCTGTATGGGGATCCGGAGAGAGGCGTAGTGGCCGACGGGGTAGGGAAATCGGAGATGCTGAGCTGGAAGAATTAGAGGCAAGAGTAACCCGAAGGGTACGAGAGGAGACTATGCAGGAGATGGACTCTAAAATGGATTCCATGGTTCAAGAGAAGTTTATGTTATTTGCTAAACAGATTGGTATTCAAATTCCAACTGAATTGTTGGAAATGAATAACATAAGCCGGACGACTCCACAAAATCCTAGTAGTTGCCAATCAGTGGGTGATGATCCATTTGCAAACATACAGGTATCTAAATTTTTTGTAATGATTTATGTCTGCTATATTATTTAACTTTGTAACTCATTTTTTTTCCAATGATTTTTGTCTATCTTTGTAAGGAACCGGTTCCATGTCGGCTATCATTGTTGAAAAATGACTCTGAGAAAGTCATTGTCGCTGAAGGTACATATCATCCGGAGTTAATCCTTGATCATCATAGTAACCTCCTTCCAGATCACGTGAGGGTAAGTGTTGATGATTTTTTTGACGAGTTCAAAGAATTCTCGGTTCCAGTTCCTTCTTCAGTCATCAAGAAACTTAAGCATGCTCACGGTACCTTTACGCAATGGCCGAAACACTTGGTTTCACTCATGCATGACAAGGTAAATTATTTTTATAACAATTTACATAGTTTGCCATACAAAAATATAGCAAATTGTTGAATAGATTTGCTTTACTATTGATTAGGAATTCATATCAAATAAGAACGATGATAACAGTAAAGCGGCCAAAGAAAACATGCAAGACAAAGTGAAAGAGGTTGAAAGTGGTCACGAAACAAAAGAGTCCAACACCAATCCAAAAAAAGTTTTTCTTATGGATTATGCTTTGGAAAACTTGTCTCAGAAGTGTGGGTCTTTGAATAGTTTGTTATCTTCATTACCCGAAGGTGAAACTATTAAGGTAAAGGTTGATGCATGGACCTTTAGTTATGAAGACAGTAAGGACATCATTATCAGACTTGAAGATGTCAATCAACTTCTCACGGGAGCTTGGCTGAATATTTCAATTTTGCAAGTTTTTATGATGTAAGATATTCAAATTTCACTCTTCCCATATATACTTTATTACCATTTATAATATTATTACATGATATATATGTAGGGCCTTGAGTGACTTACTCGATACGGTGGATGTGGCTTCCATTGGATTCATGTGTCCGGAAATGATTTCAGAAACCTATTTGCATAGTGATGCAGATCGTATCCTACTATACATGACACATGTGATGGAAAAACAAAAATCTAAGCACTTCATCTTATGCCCATACCATGAAAAGTATAAACTTTGAACTTGTATTTATTTGAACTTTGCAAATTGTTGATATTATTTTATTAATTTACAAATGTGTGTCTAATTTATTATGTAGGAATCATTGGGTTCTTTTGGTTTTATGCATGGCTAAGCGTGAAGTCTACATCTTTGATTCTTTGAGGCAAAAGCGAAATTTAGCAATTAAGTTTGCAATGACAAAGTAAGTCTAATTTGTTATGAATGTCATGATTTTATGTGTGTGTAAGGATAATAGATGGTTTTAAATATTATGTATAAATATGTTATTTGTTTCAAATGTGTAGTGCTTTTCGAAGTTACAAGGCATTAAGTGGACAATCTAGGGGAAGTAAATTGACATGGCATTTGGGACAGGTTAACTTAATTCCTTTGCCCAAACTTCTAGTTTCTTAATCATTTAAGTATAGCAATTTAATTCAACTTATAATTGTAATGCATATAGTGTCCTCAGCAATTGGGTGGACGTGAGTGTGGCTACTACGTCATGCGTTATATGTACGAAATACTTGAACATCATCGTAGCAGTGAGGATCTTATAAAGGTATGAGTTGTCAAATATTTCATTTTATTTGATAAATACAATTTAATGTTAGCTACAATCTTGAATTAAAACTTTCCTCTTGTATATTTGTTGTGTTGATAGGATTTTTCAAGAACTACCCCGTATACCGAGGAGGAGATAAATGAGGTTCGAGATATTTGGGCAGAGTATTTTATATGTAATGTTGAACTTTAGATTTACACTTAGCAAATGTTGGACTCTTGTTTTGGATTATTCATTATAGGCTTGACATGAATGAATTTGGTTGATTGGTGTTGGGATGATGAGTATATGTCAATGATATTGAGATGTAAACTTATTGGACAAGTTGTTAATATAATGCCGTCATTCCCAATTTTATGGGTAGGCCAAAATACAAAGGAAACTCTGCCGAAATTTACATTTGAATTCATTAATATTGCATTTTAATTTCATTTATTGGTGTTAGCATATTACGTTATACCACTACCAAGGGAAACAGGTACTACAACAATATACAAGCAATAAATATAATTAAAAAAAAAATTCACATCATTTGCGTCACTCATTAAACAAACAAGTGACGCAAATAACTCGCTTTTGAATTGTCTAGTAAGCCATTTGCGTCACTCAATAGTTAAGCAAGTGACGCAAATTCAAAACAAGAAGTTGCTCAGAACAGTTATTTGCGTCACTCCATTATAAAACAGTGACGCAAATAACTTTCATTTGCGTCACTTGTTTATTAAGGAGTGACGCAAATAACTGTTATTTGCGTCGCGCCTAGGAGCGACGCAAATGATTTAAAAGTCATTTGCGTCGCGAGCAGTTGCGTCGCTCATAGAAGCGACGCAAATACACTATAAATGAGCGACGCAAATGAACGTTTTTCTACTAGTG

>Cvul_Jozin_consensus_Chenopodium vulvaria; DNA-transposon, TIR, CACTA; complete element

CACTAGTACAAAAACGTACATTTACTTCGCCTTATTTGCATCACTCGTAGAAATAAGTGAAGCAAATGATAAAATTTGGCTTGAAAATAAGTAATTTGCGTCGCTCACATATTATAAAGTGACGCAAATATATGGTATATGTCATCCTATTGTGATTTGCGTCGCTTTGAATTAATAAATCGACGCAAAAGACAATGTTATTTGCGTCACTTGTTTAGTAATAACCGACGCAAATAATTAAGAGAACTAATTGCGTCGCTTTTGTTTTAAATACTCGACGCAAATTTTCAATGTAATTTTAGTTAATTGCGTCGCATGTTTATTAACAAATGACGCAATTAACTCTTAAACCTAATTTTATCATACGAATTAGAAGTTGAATGAAACAGTTTAAAAAAAATAAATTAAGCATTGCCATCTTCTTCCTGCGCTGCTACTTGCTGCTCCAACCCAGGCCACTTGCTACTCCAACCCAGGCCACCTGCTACTGGTTGTTGCGCTTCTGCTGCTTGTTGTTCGAACTCAAAAATCCCAGAAATTTACTGCTGGTTGCTGTGCGCTTCTGCTTGCTGTTCGAACCCAAAAACACCATAAATTTTGAGCCCCAAAACCCCCTGAAAATTACAGCATTTTCCCATAAAATTCGTGCAAAACCACCCAGAAAATCGTCCACAAATCGCAAGAAATTGCACCCGAAAAGCCCAGGAAAATCGAGCAAAACAACCGAGAAAAAATCCCCACAATTTCGACCAAAATCCCTAGAACAATTTCCTGCATTGTTGTTCAGTTGTTCGAACCCATAAAGCGACGCAAAGTATTCGAAAATTTGCTTAATTTGTCTGAAAATTCGGCCCCCAAAACCATTGTGATTTTTAGGGTTAAGGTTCAACTTAGAGTTTCAACTTGATTCTATTGCATTTGGGCTCTACATTTGGTGAATTTTTGAAGTCGATTTCACCCAAGGTATGATTTATTCTTCTATTTTCTTCACTATTTTCAGAAAAAACATGGAATTTAGGGTTGAACTTGTGCATGAACTTGGTTGAAAAATGTGTTATACCAAATAATAATAATAATATGTTTGGCTGATTTGGGGCCGAAATTAAAAAAAAATGATTTGTACCTAAAACACCATTTGAGTTCGTAGTGTGTGTAGTTCGGAACTAATTGTGTCGTTTTACAACTAAAAGTCGAGTTGTTAAGTATGTAATGGGATTTCATGGTTCGATTAATGTTGATTTAGGAAATAGAATGATGAGTGGTTCGATTAATGTTTATATAGTAGAAAGTTTATTTGATATACTTGCATGCCAGCCTGCTTCTATGGTATTAAGGTCATTGCCAAATGAACCAGAGATGATCCACAGTTGAGACAAGCTAAATTCGTATGCATTAGTAACTTGAGGTGTCCAAACATTCATACTGGCTTTAGCACCATAAAATTGTTGTCCATTAACGAATGCCACCACGTGCTGTGAAACCGAAAATCATGTGTTAGACATCCATACTTGCTGTTTTTAATCATAGCTTTCGAATGTGATCGATACCAATGTGCCATTGTTAAGAAGCAATGCTTCGTTTTTTTTAGCATGTTTGCTTCTGCATTATATTTACTCTGTTAATCTGTTTGTGTTTTGATATTCAGAAAGTGTTGAACATTCTCTAACCTCATGACCACTACCATTCGAATCTCGCCTCACAATTCTTCTAGGCTTTCTTCCAAATCTTCCAAATTTACTATCTCTCAAAAAATCCTCTTCTGTAGTTCTCCTGATTGGTATAGTTCCATTTGGACATGATTCTCCAGATTCTGTCCATTGCTGAATGCTGTGTGTTACTGTGATTCTTTTTATTTTGTGTGGTGTTTCTTTTTTTAGTGTGGAATTTCTTGTTCTTAACTTGCTACTCAGAAATTTTTTATGGAATAGGGGTTTTATTGTTAAAGGGATTAAGGTAAATGATTATTTATCTTGATGGAGGTAATATCTTGCAATAATAATATTCTTAAACAAAGAATTAAGTTCTATAAAAAATCTCGTTCACTAATGTAATGGAAAACCTTATGCTTAAATTAAAGTTAAACTCAAATAAAAATACGTAATGACAAATAAATATACAAAATAGAATTATAAGACCCCACTTTTCTTCTGAAGCTTGATCTTTTCTTGCATTTTTATCTTGATAATTGCCTATGCTTTGCTAATGCACGAGTAAATTGTTAAAATGTTTAAGATTTTAAATTAATAGAACTAACAATCCCATTATGTTTTACTATTATCAAGTCTCACTTGGTTTTGTTTACATCATTCTATTTATAGATTAGTATAAACACAACATAATATAATTCCCTTTGGAGAATGGAGTTAATCAATTTTCTTATTTTCATATTAATTTGAATGAATGTACAAGAATTTGGCTTGTCTTCCTTTTCTATCCACAGTTTCGGTTTTACTATAAGGTACCTTATAATAATAATTGATGGTTTTTAATAATAACATGAGTTAATCTTTGAATGAAAATGTTTTGTACCTTATAATCTTGTTTATGCTTAAGGAACTACATATGTTAAAAAATATACACATTATCTTAAAATAACTTTTTCTATTCAGTGGTTGAAAATGGATCGAAGTTGGATGTATGCTAAACGAAATTCTACAAGATTTTTACAAGGTATTGAGGAGTTTAGTAGTGTTGCCCTAAAATACCAAACAGAGAATAAATCAAAGATTATTCTTTGTCCTTGCTTTGACTGTAATAATTCGAGGGGGTATCGTGATATAGATGATATTAAAGATCATTTAATTCGTCGCGGGTTTAAAGATAACTATACAAGGTGGACATGGCATGGTGAGAGCATAGATCATGGGGCAAGTTCAAGTTATTGCCTGAGGGAAGATGAGAATCATTGTGATGATGATAATGAACATGAAGGTGAGCATATCAATACTGCTGATATTGTTGTTGAAAGTGAAAATGAAGTAGAGAAGGACAGGATAGATGAGATGATGCATGATGTAGAAGACCATTTCACAGAGTGTCCCTCTACGTATGACAGTATTTTGAGAGCTGCAGAGACACCGTTATATCCTGGTTCTACAACATTCACTAAACTTGGTGCCATTTTCAAATTATTCAACATAAAGGCGAGCAACTGTTGGACTGACAAGAGTTTTACGCAGTTGTTGGAAGTGTTAGTAGAAATGCTTCCTGAAGGGAATGAACTTCCAAACTCCACCTATGAGGCCAAGAAACTTATGTGCCCTATGGGTATGGAGTATGTGAAGATACATGCATGTCCCAATGATTGTGTGTTGTATCGAAATGAGCATACTGATTTGCATGAGTGTCCAAGGTGTGGAGTTTCTCGTTACAAGATGAATGAGAATGGTGAATTGTGTAAGAAAGGGTCTCCGGCTAAGGTATTGTGGTATCTTCCAATTATACCGAGATTTAAGCGACTTTTCTCAGATGAAAAAAATGCAAAATTATTGAGGTGGCATGCTGATGGGAGGAAGAAAGACGGGTTAATGAGGCATCCAGCTGATTCCCCGCAATGGAGGAACATTGATCGAAAGTTCAAGGTCTTTGGTGAAGAAGATCGAAATCTTAGGCTTGGTCTTTGTACAGATGGAATGAACCCATTTGGGACACTTAGTACCCAATACAGCACTTGGCCGGTTCTTCTAACTATCTACAATTTGCCTCCTTGGTTATGCATGAAGTCTAGATACATTTTGTTGTCGCTTCTAATATCGGGGCCTAAACAACCCGGAAATGACATTGATGTGTATCTAGCGCCTCTTATTGATGATTTGAAATTGTTGTGGAATGAAGGTGTCCCGATGTTTGATGCATATAGTAAAACCAATTTCACTTTACGTGCCATGATTTTTTGTACGATAAATGACTTCCCAGCTTATGGGAATTTGTCAGGGTACACTGTGAAGGGAACAAATCCATGTCCTATTTGTGAAGATGATTTAGAGGCATTACGCCTAGACAATTGTGGCAAGCATGTATACATGGATAATCGTAGACATCTTCCTGAAGACCACCCTTTTCGAAAGAATAAGAACGCTTTTAATGGAAAAGTGGAGATGAGAGAAGCTCGTGTCCCTTTACGTGCGAGTGAGGTTTATCAGCGGGTTAAAGACATTGAAAACGAGTTTGGTAAGCCTTACAAAAATAAGTCCAATGGGGGTTATAAGAAGAAGTCTGAACTATGGTCTCTCCCATACTGGAGACATTTGGAAGTTAGACATTGTCTAGATGTAATGCATATTGAGAAAAATGTGTGTGATGCCATTGTGGGAACATTATTGAATATGCCAGGAAAGACAAAGGATGGAGTTAAAGTAAGAAAAGACATGGCTGCTATGGGTCGTTCAGAGTTGGCACCCGAATCTCGAGGAAAACGATGGTATCTCCCCCCAGCTTGCTTTACCTTGTCCAAAAAGGAAAAAACTAGCTTCTGTGAGTCATTGCATGGTTTAAAGGTCCCAGCTGGATATTCTTCTAATTTTCGTAGACTTGTGTCGATGTCTGACTTGAAATTGGTTGGAATGAAATCTCATGATTGTCACGTGTTGATGCAGCAGTTATTACCAGTTGCAATTCGAGGAATTTTGCCAGCTCAAGTTAGGTATACGATTACAAGATTGTCTGTCTTTTTCAACACTATTTGTAGCAAGGTGATAAATCCAAGTATATTAGATGACTTACAAGCAGATATAATTGAGACAATGTGTCGATTTGAAATGTATTTTCCCCCTTCTTTCTTTGACGTGATGCCTCATTTGGTTATTCATCTTGTACGTGAAATTAAACTTTGTGGGCCCGTGTGTATGAGATACATGTATCCTTTTGAAAGAGAAATGGGTGACTTAAAGGGAAAAGTCATGAATCCGGCCAAACCTGAAGCTAGTATTGTCCAACGAACAGTTGCTGAGGAAGTGGCAGCATGGGTTGCTCAGTATCTTGCACGTGCACAGAAAATTGGGTTGCCAAAGTCTCGACACGATGGGAGGCTTGGGGGTCAAGGTACTGTTGGTAGGAAAAGGATATCAATGGGTCTTGAAATGAAAAATAAGGCTGAGCTTTTTGTGTTGCAAAATCTTAGTGAAGTCCATCCTTACTTGGACGAGCACATGATTTTTCTTAGAAATAAATATCCTTCAAAAAATGATCTTCAGCTGATAAAGGAGCATAATAGTTCATTCGTTACATGGTTCAAGGAACGTGTGATGTCTCAGTTGTCCACAACACCTAATGATGTATCCGACACATTGAGATGGTTGGCGTATGGTTCTAAATGTCAAGTCATTTCATATGAGGGATACGACATCAATGGGTATTCTTTTTACACAAGTCGACAAGATGACAAATCGACAATGCAAAATAGTGGTGTTACAGTAATAGGTTTGTCATCTGAGTATGTTAGTGCACGTGATAAAACACTTGTGGATAAGAGGAAAATTTATTATGGAGTCATTGAAGAAATAATAGAGCTGGACTATGTTGATTTCAAGATTCCTCTATTCCGATGTAAGTGGGCTGATAGTAGCCGTGGTGTAAAAAAAGATGAACAAGGGAACTTGACCCTTGTAAATCTTGGTCGACCAGGGCATCTAGCTGATCCATTTATATTAGCATCACAAGCAAAACAGGTTTTTTACATGGTTGACCCAGCTGATCGTAAATGGTCAGTTGTTCTAGAAGGCAAAAGAAGGATACTTGGCGTTGAAGATGTGGTTGACGAGGAAGAATATGATGAGCAGTTCAATGAGTCACCACCCTCCTCATGGAACATCCCTCCAATAGTCGATGATTTTGACACAACTTTAAAACGTATAGATCATAATGAAGGATTTTATGTCGTAAAAGAGAAGAATGATGTAGGTATGAAATTTGTATTTTTTTACAATGTGTAACTAATTGCACAAATTGCATATTTTTTTTTACATTTACAAGTTTATAAATTATAAACTATATAAATCAATGTCTTAAAGGATTTATGTTTTTTTTTTCTTTTTTTGCTATATTGCAGGTATATAATAATGGAGGATGAGGATATGCATAGTCGCTCCTTATCTTCGCAAGGTGAGGAAAATCTTGAAGGTGAGCCACACAATCAATCACAATCAGAGCTACAAATGGAAGCCTCACAGAAGAAGAAAAAGAAGCCAAGAGGTCCTTCAAAGGGCCTGAAATCCATGCCTGGGGTTCCAAAAGTGCTTGAATGGGATGAATTGTATCGACCAATTGGAAAATGGGTAAAAGCATACAAAGTTCATCTTGGTGAAATAAGCCGTGCAAAAGTGTCTATATTGATTAAAGATTGGAATCAAGTTCCACAAGGAATAAAAGACACATTGTGGGAAGATGCCATGGTAATATTCACTGTCCAATGTTAAATTTTACAATATTATATGATCATTTTAAAATTGTTTGAATATGAGACTTCTTTTTATGTTTTATTTAGAGAGAGTTTCAAATCGAAGAAGATGAAACAAAGAAAAATAAGGTCCTACGCACTTGTGATAAGCGATGGAGAGATTTTAAAACGAAATTGGTGAGCGGTTGGATCTCATGCACAAGGAAAATGCCGGATGAGAAAAGAATGCCCTATCAAATTTATGATTTCATAACTGAAGATGTGTGGAATAAATTTGTGGAGGAACATAGCACGGATGATTTTAAGGTATGTATAGATATTATATATATATATGTACCATTATAATTAAATGAGTGACTGTTATTAATACTTTGTTTTTGGATTCTTGTGTTAACTATTCTCCCTTTGTTCTTGTAAAATTAGGAAATTAGTGAGAAGGCAAGAAAGAGCCAGTCTTTCAACAACTACCCTCACCATTTAGGACAAAAATCATATGCTGAAATGAATGGTGTATGGCGTAGAAAAGGATATATTCCCTCATCATCTTCAGCATCCACTACTTCCTCTTGCTCTTCAATTGTGTCTAGTTTGCCAGATAGGACATATGCTTGGCTTCTAGCAAGATCAGTAGAAGATGATAAGGGAAATCCTTATTTGCCTGATGAGAAAACAAGAGAGGTGAAAGAATCTATTGTAAGTATATAAAAAAACTTGTTATTCCAATGTTAAATCTAGTTTCTTAAGTACATGTATTGCAAAATAATTTACTAACATTAACCCTTTGTATAGGATAATTGGAGAAAGCAACAGGCTGATGGGAAATTTGTTCCTACAAGGCATGATGATGTCTTGTCTCGTGCCCTTGGGAAAAAAGATCGTAATGGTCGGGCGTTAGCATTTGGCAGTGGAATAGGCATTAAAGCTGTGTGGGGGTCCGGAGAGCGGCGTTGTGGCCGACGGGGTAGGGAAATTGGAGATGCTGAGATGGAGGAACTAGAGGCAAGAGTAACCCAAAGGGTGCGAGAGGAGACAATGCAGGAGATGAACTCTAAAATGGATTCCATGGTTCAAGAGAAGTTTATGTTATTTGCTAAACATTTAGGTATTCAAATTCCAACTCATCTTATGGAATTAAATAACATAGGCCTGACTACTCCACAAGTTCCTAGTAGTTGTCATTCAGGGGGTGATGATCCATTTGCAAATATACAGGTATGCTAAATTGATTTTTTTAATGGCTTGTGTTTACTATGTTATTTAACTTTCTAACACGTGTTTGATAAAATGTACAAGGAACCTGTTCCATGTCGGCTATCGTTGTTGATAAATGGCTCTGAGAAAGTCATTGTCGCTGAAGGTACATTACATCCACAGTTGTTCCTTGATCATCATAATAACCTCCTTCCAGATCACGTGAAGGTAAGTGTTGATGATTATTATGATGAGTTTAAAGACGCTCCGGTTCCAGTGCCTTCTTCAGTCATTAAGAAACTTAGTCATGCTCATGGTACTTTTACGCAATGGCCGAAACACTTGGTTTCACTTCTGCATGACAAGGTAAATTATTTTTATAACAATTTAAATAATTTGCAGTAGAAAAATATAGCAAAATTGTTTTCATATATATATTTGCTTGACTATTAATTAGGAATTCATATCAAACAAGAATGATGATAACAAAAAAGAGCCCAAAGGAAAAAAGACGAAAGGAAAAGAGGTCGAAAGTGGTCACGAGACAAAAGAGTCCAACATCAATCCGAAGAAAGTCTTTCTTATGGATTTTGCCTTGGAAAATTTGACTCAGAAGTGTGGGTATATGAATAGTTTGTTATCTTCAGTACCCGAAGGTGAAACTATTAAGCTGATCGGCGATACAAGGACCTTCAGTTATGAAAAACAAAAGGACATCATTATCAAACTTGAAGATGTCAATCAACTTCTCACGGGAGCATGGCTTAATATTTCAATTTTGCAAGTTTTTATGATGTAAGATATCTAAATTCCCTCCCCCACCCCCCCCCCCAACATTTTTTATTTCCATTTCTAATATTATTACATGATATGTAGGGCCTTGAGTGATTGGTGTGATACGACCGATGTGAATTCCATTGGATTCATGTGCCCGGAAATGATTTCAGAAACCTATTTGTATAGTGATGCAGATCGTATCCTACTATACATGACACATGCGATGGAAAAACAAAAATCTAAGCGCTTCCTCTTATGCCCATACCATGAAAAGTAATTTTCAACTTGTTTTCTATTTGAATTTTGCAAAATGTTATTAATTAATCATTTACAAATGTGTTTGTAATTTCTGTAGGAACCATTGGGTTCTTCTGGTTTTATGCTTGGCTAAGCGTGAGGTCTACATATTTGATTCTTTGAGGCAAAAGAGAAATTTAGCAATTAAGTTTGCATTGACAAAGTAAGTCTGATTTGTTATGAAATCTGATTTTATGTGTGTATAAGGTTAATAATTGTTTTTATTTAATGTGCACATATATATTACTTGTTTCAAATGTGTAGTGCTTTTCGAAGTTACAAGGCGCTAAGTGGACATTCTAGGGGAAGTAAATTATCATGGCATCTGGGACAGGTTAAACTTAATTCCTTGCCCAAATTTCTAGTTTCTTATTACTAATGTTATGCATATATTCCAACTAACTTCAAATAACCATTTGTAATGCATATAGTGTCCTCAACAATTGGGTGGTCGTGAGTGTGGCTACTACGTCATGCGTTATATGTTTGAAATAGTTCAACATCATCATAGTAGTGAGGATTTGATTACGGTATGAGTTGTCAAATATTTCTTCTTTATCATAAATCCATTTCAAGTTAGCTACACTCTTGAGTTAAACCTTTGCCCTTGTATTTGTGTTGCATTAATAGGATTTTTCAAGATCAACACCTTATACAGAGGACGAGATAAATGAGGTTCGAGATATTTGGGCAGAGTATTTTGTATGCAATGTCGAAGTGTAGCTTGATACTTAGCAAACTCCATTGTACATTGTTGGACTCTTTTGTTTTTATTTCTTAGGCTTGAAATGTTTGTATTTGGTTTACCAACAATTTGGGTTGGTGTTGGTGCTTGTGTTGCGTGATGACTTGATATAGAAATGTAAACTTATTGGACAGGATATGTCGCCGTCAATCCCGATTTTATGGGTAGGCCAAAATACAGAGGAAACTCTGCCGAAATTCATACTTGAATTTATTATTTTATACTTATTATATCTTGTCTTACATTATTATATTATAACGAAGGAAAACAGGTACTATATACATGTAGCTTTACTGTGCAGTATCTAAAAATAAATACATAATAAATAAAATAAATATAATATTATATTATTATATATATATTTGCGTCACTTGTTACATAAACAAGCGATGCAAAACTCTCATTTTCTATTTTGTCAAAAAAGCTATTTGCGTCACTCATTTGTTAAGAAGTGACGCAAATAAAAAAAGATAGGTTAGTTAAAGGAGTCATTTGCGTCACTCCTTTAATAAACAAGTGACGCAAATAACTCTTATTTGCGTCACTCGTTTATGGAGGAGTGACGCAAATGACTGTTATTTGCGTCGCATATACGAGCGACGCAAATGACTTAAAATCATTTGCGTCGCGAGCTGTTGCGTCGCGCATAGAAGCGACGCAAATACACCCTAAACGAGCGACGCAAATGAATCATTTTCTAATAGTG
