# Supplementary material for: The structural diversity of CACTA transposons in genomes of Chenopodium (Amaranthaceae, Caryophyllales) species: specific traits and comparison with the similar elements of angiosperms
Source: Mob DNA. 2022 Apr 4;13:8. doi: 10.1186/s13100-022-00265-3 (PMC8978399; doi:10.1186/s13100-022-00265-3)
Supplement: Supplementary file 5 — Additional file 5. Pair wise distances between ORF 1 of the four Chenopodium on protein level. [file 13100_2022_265_MOESM5_ESM.docx]

S5. Pair wise distances between ORF 1 on protein level

| Species | *C. pamiricum* | *C. sosnowskyi* | *C. iljinii* | *C. vulvaria* |
| --- | --- | --- | --- | --- |
| *C. pamiricum* |  | 0.220 | 0.583 | 0.538 |
| *C. sosnowskyi* | 0.220 |  | 0.578 | 0.532 |
| *C. iljinii* | 0.583 | 0.578 |  | 0.556 |
| *C. vulvaria* | 0.538 | 0.532 | 0.556 |  |
