# Supplementary material for: The structural diversity of CACTA transposons in genomes of Chenopodium (Amaranthaceae, Caryophyllales) species: specific traits and comparison with the similar elements of angiosperms
Source: Mob DNA. 2022 Apr 4;13:8. doi: 10.1186/s13100-022-00265-3 (PMC8978399; doi:10.1186/s13100-022-00265-3)

**S6. Comparative analysis of contigs and clones form *Chenopodium iljinii* genome**

Analysis of the assembled contig 20 (*C. iljinii 1*):

>Cilj_assembled_contig20_iljinii_1

TTCAAGCATCCCAAACTTGAACCCCACTGTGATCATACACTTAAGCATGGTGGCGTCCAAAGGGAAAGAAGCTAGAGCAAAAGAGTCGCCAACAAGTTTTAGGAACTTGTAAACCGTTCACAGGGTGAAGGTTACACCCCTTCACGATCCTAGAGATCGCTAACTCCATAGTGGTCTGGAACCATGACTGGATATTCGGGGATGAAGGTACAAATCCAACCTAAAAGAGATTGAACCTGCCCTAAAAATAGGCCTCTACTTTATTCTTCTCATATTCATGTGCGCTTTCTGATTTGTTGCCTCGTATCTCTTTCTAATAACAAAATACACACGTTAGTAACTTCAAGGAAAATATTAAATCAAACAATTAAATTAAATAATTGAAATAGGGATTTGGGACCAAACCCTACTAAAATTTGTTGATTTTAGTACCGATTTATTGCGACTGTCATTAAAACGGTCGCAATTAATATTAATTACGACCGTTTTATAGCATGTCGTAATTTTGATTAATTGCGACCGTTATTTTAATTATTGCGGCCGTTATACCGGTCGTTGAAATTATGTGGTTTTTTTTAAAACAATTACGACCGGTCTGACTGTTGTTGAACCGGTTGTAATTTTTTTTAACAAATTAAAAAGAAAGTCATTCGTGCTTTCTTAAAGAAGGGGAAAGGGGAAAGAAGAAACAGAGAAGAAGGGGGAAGAGGGAGAACAACGAAGAGGAGGTGACGGTCGCAGACTCCGCCGTTCTTCCCTACGACGAGCTATCTCCGGCCGGCGACGCTACACACAGGTATGTCATTTTAATTTTTTGTTTTTTTGAATTTGTAAATTTTCGTTAGTTTGTGCGAAAACCCTAAATTTGGGTCGGAAATCAACCCCCACGCAGGACCACCGACGCCGGACTCCCCTCCTGCCCGTCCCTCATTTGTGTTCCGACGGCGGTACTCAGGTAAGTCGCTGCAATTTTGTTTATGTTTTGAAATTTAGTTAATTTGGGCGAAAACCCTTAATTTGGGTCAGAATTCAACCACTTCGCAGGCCTACCGTCGCCGGTTTCCTCTTCTATCCTAGCCTCCTCCGTTGATCGTCGACTAAACTCAGGTAAGTCGAAGGAATTTTTTTTTTGAAATTTAGTTAATTTGGGTGAAAACCCTAAATTTGGGTCACAAATCAACCCCTTCGCAGGCCTACCGTCGCCGGTTTCCTCTTCTATCTTAGCCTCCTACGTTTTTCGTCGACTAAACTCAAGTAAGTCGAAGGAATTTTTTTTTTGTTTTTTTGAAATTTAGTTAATTTGGCCGAAAACCCTAAATTTGGGTTACAAATCAACCCGTTTGCAGGCCTACCGTCACCGGTTTCCTCTTTGTCCTAGCCTCCTCCGTTTTCCGTCAACTAAACTCTGGTAAGTCGCTGCAATTTGTTTTTTTTTAAAAAAAATTTAGTTAATGTCGGCCAAAAACTCTAAATTTGGGTCACAAATCAACCCAAATGATTTGTACTACAGTTTTGTTTTAATTTGATGATCATATTATAGTAATGTTGATCATATTTTTTGTGGGGTTGAGATTTTGGTTAATGTTTGGACTTGATATTGTGCTTGCTTTTGATATGGGTTTTTTCACTATGAGTGGACACTAGATGGGATATACTGGATTCGCGGAATTTGCACTTAATTTCTTGAGAAATGGGTGGTCTAGATTTGAATTCGGGTTTCATTGGAAATGTGAGTGATCGGTGGTTACGGGGCATTTTGTTTTCATCTAGATTTCACTTTCGCGATATAGTACTACATGTAAAGACATTGGTTACTTCATGAATATATATGATTCTAGGGGCTGGTGTTTTGGGTTCAAAAGCGAGCTAAAATGGGCGGTTGTTGTAGCTGAATTGGGGGCCGATTGGAGGTGGTATGTGGCTGGGTTGGTGTGGATTTCAGGCGCACTTTGGAGGGGACAAAAGTTGGTGGTTTTGGTGGTTGTAAAGTGTTGTTTAAGGTTTTGGTGAAATGTATGTAGTATCTTGCATTTTCACATGTAAAATGTATGTAGTGGGTAACAAATTTAAGTATATTTAGATAGCTAGGAAGTCTAGGATCAAGTCTAGTTTTCTTTGTTACTATAGGTCATTTGGTGGTTATTTATGATTTTCATGCATGCTTATTACAATTATAGAATACATTCAAGTACCTTGTTATTTATGATTTTCATACATGATTATTACAATTATTTTTGATAAATTATAGAATTGTTTTAGCTCATCGAAATTAATTACATGATGATAATCCTTGATTAAAATGGAGAAATGTGAAGTGGGGACAAATCTATTATTAATTTCTATGGTTCTTTTTTTTTTAATTTTTAATTTTTTTAAAAATATGTATGTTTATATGAGGGGTTGTGATAATTTTGATGAGTAAAAAGTTAGAAGCTTGTGAAATCAACATAATTATGTTTAAATCTGTTGTTAAAGAACACTACTTATGAAAACAACTTTCCCGTTCAAGTCATGATAAGGACCTTTGAATATCAGTTCGATTCTAATTGTATCTTATGTCATTATTTTAATGAATACAAGTTCACTGTGAACGGTTAATGTGATTTGTTTTTCATAGTGTTGTAGAATTTGTCACCATGAAAAGGAAAGAGCGTTGGTGGATGTATGATAGATACGATGGCCGTAATGCCCGTAAACTTAGGCAAGAGTTTGTGGATGGTGTTGAACAATTCATACGGTTTTGTGAAGCTCATCCTGAGAGCCCGGGCATAGATGAAATAAGATGCCCTTGTCTGTTATGTAAGAATAGACGTCTTCATGATGCCAACACAGTAAAACTCCATTTGTATAAAAAGGGGTTTATACCTGATTACTATGAGTGGGTTTCTCATGGTGAAGGGTTCCCTACATCAAGTGTAGAGCAACCAAATACTTACCGTGAAATGGTTCTCGATGCTCTTGGGAGTAATCATGAACCATCGGGTGATGATAGTACTAGTGGTTCAGATGAAGAGCCAAATGCTCAAGCAAAAGCATTTCAAGAGCTTTTAAAGGCCGCCGAGCAACCACTGTACGAAGGTAGCTCCATGTCTGTGCTAGAAATGGCATCTCGAATTGTATCTTTGAAGTCAGAATACAATTTGCCCCATAGGTGTGTTGATGGGTTTGCTTCATTATTGAACGAAGCGATTCCCGACAATAATAAGATGGGGAAAACATTCTATGATACAAAGAAGATTCTTAACGGACTTGAGCTTCCGCACGAGAAGATCCACGCATGCCCAAAGGGTTGTATGTTATTTTGGAAAGCTGATGCTGAGTTGGATAAGTGTAGAGTGTGCGAAAGGGACAGATACAAGAGGACTTCAAAGGGGTCATTGGTTCCAATAAAAGAACTCATCTATTTTCCCATCACTCCAAGGCTACAACGGTTGTTTGCAACAAAGAACGTTGCGGAGGAAATGACCTGGCATTCCAAAAATCCAAGAGTCCAAGGCACAATGGCGCATCCTAGTGATAGTGAAGCTTGGAAGCACTTGGATAGATCCTTCCCTGATTTCGCATCTGAACCTAGAAATGTGAGACTTGGTATGTGTACCGATGGGTTTGCTCCTAATGGCAAGTTTGGGGGTCAGTACTCATGTTGGCCAGTCATTCTGACCCCTTATAACTTGCCTCCATCGATGTGCATGAAAAAACCATTTATGTTCCTTTCTTTGCTCGTTCCTGGTCCTAAAAATCCGAAGGGGAACTTGGATGTGTATATGCAACCGTTAATTGAAGAATTGAAACAACTGTGGGATGTTGGTGCAACAACTTACGACATTTCGCAAAGAAAAAACTTCAATTTGCGTGCAGCAATCTTGTGGACTATAAGTGATTTCCCAGCCTATGGTATGCTTTCTGGATGGGCAACAGCTGGTAAAAAAGCATGTCCATATTGCTTGGACAAATCAAAAGCTTTTTGGCTTGAACATGGTGGTAAAATGTCATGGTTTGATTGTCACCGCCAATTTCTTCCTCCTGAACATCCTTTTCGAAACAACAAAATTGCTTTTTGTAAGAATAAGGTTGAAAAGGGGACACCGCCTCACATAATGTCCGGCGACGAGCTATGGCAACAAGTTCAACACTTGGCTAAGGCAACAGATGGACCTGAAGCACTCGCAAGCTTGAAAAAGAAGAAATTAGGGTGGTTTAAACAAAGCATTCTTTGGGAGCTACCGTACTGGAAGACTCTTCTCCTCCGCCACAACCTCGATGTCATGCATATTGAAAAGAATTTCTTTGACCAACTAATTCACACAGTGATGGATGTGAAAAAACATACTTCAGACACAGTTGCATCTAGAAATGACATTGCCAAATATTGCAAACGACCACAACTACATGTGACGGAGGATGCAAGAGGGAAAGACACTTTGCCGAAAGCCCCATTCTCACTAGACAAGGCTCAAAAGAAAGTGTTATGCGAGTGGGTAAGGAACTTGAAATTTCCCGATGGTTATGCTTCTAATTTGAGTAGGTGTGTAGATTTACAAACATGTAAGCTTCATGGTTTAAAGAGCCACGACTGTCATGTCTTCATGGAGAGATTGTTGCCAGTTGCTTTGAAGGAATTGCTCCCGGTCCATGTTTGGAAAGCTATTACTGAGATTAGTCTTTTCTTCCGTGACCTTTGTTGTTCCACCATCAAATTGAGTGATATGGAAAGATTGGAACACAACATTGCAGAAATTCTTTGTAAGCTCGAGAAGATATTTCCACCAGCGTTCTTCAACTCTATGGAACACTTACCAGTTCACCTGCCTTATGAAGCTAGGTTATGTGGTCCTGTCCAATATCGTTGGATGTATCCGTTTGAAAGGTTTTAGCTCCTACAAACTCATAGCTGCCATTTTCGTTTATATACCTATAGTTGTTTGCATAACATATAACTTAATCAACATTTTTATTAATAGGTTCCTTAATCATTTGAAACGTAAAATTGGCAATAAGGCTCGTGTTGAAGGTTCAATCTGCAATGCTTATCTAACGGAAGAGATAGGGTACTTTTGCTCTAACTACTTCCAACAAGGAGTGGATACTAAAACACGTGACTTAGGTCGTAATGTTCATGATGATGTTGAAAGCACTTTGGATGATAGTGTTCCGGAATTGTTTAGGGTTGATCATGGTCATGCTTCTACTGGTGGAGTGCGCCGCTTTTTGGATGAGAAAGAACTACAACGTGCTCATTTATATGTTTTGGGAAATAGCGGCATTCTGGAACCATATGAGAGGTATTAAATTTGTCTTTATCACACTCACTAAGATTTCATTGAATTTTGCAACGTATCCAATGTCCACCACCATCATTAATACTCTTTCTCTGGTTATACGGAAGGGGGTTTGAGGACCACATTGTTCTAATGCAACCCAATGTTCGCAAAGAAGATGTGTGGATTAAACACGAAGCTGAATTCCTCGAGTGGTTTAGGTTGAAAGTGAGTGTGTTACGCATTTAACATTAACACGTTTTCTTACTTTGTTGTTATAACTTCACGACATATATCAATTTAATATAATTACGCTATATGAGCAGGTGATTGAAGACAATCCAAATGATGAAATATTGTTTGCTTTGGCAATGGGCCCATCTAAGCGAGTGCGTACTTGGAGCCAATTCTATGTGAACGGATACAAATTCCAAACTTATGGCCATGGAAAACAGAAGTCAACGATGAATTATGGAGTAAGCGTATCCAATGAAGATGGTGGAGATTATTTTGGTATTTTAGAGGATATCATCCAGTTGGAGTTCACTGGTGCCCTACGGACTTATAAAACTGTTCTCTTCAAGTGCAGTTGGATGGATTCTAGTAGGGGTATGAACATTGATCAATACAAGCTTGTAGAAGTCAATCACACCAAAAAATACCCCAAGTACGACCCATTTGTGCTTTCTTACCAAGCTAACCAAGTCTACTACGCACCTTACCCAAGTCTGAAAAGAGATAAGGCTCAATGGTGGGCAGTGTTCAAGACAAAGGCGAGGTCTGTTGTTGATGCACCAGTTGACGAAGACTTTCTACAAGAAACCACTGCTGACATTCCAACTCTTTGTGCTCCGGATGACATTCCAGACTATGAAGGTGATGAAGATGGATTTGGAGATTCGGATACTGATGATGAAGTGCTCCCGGATGCCCCTCCAGATGAGTCCAGTTTCGAATCTACTGATAGTGAGGACGAGGATGAGGATGACGGATTTTGGGATGACGATACTGATGATGATCTTAGTGCTGATGGGGAGGGTGATGCAATTGAGATTTAGGAGATTGATTGTAACCTTGTTTTAACTTTTTAATTATGTAATCTTAATGGATTTCAATTTATTTTAAAAGACCATCCTGGCTTTTACTGATGTTTATAATGTTTAAGATGACGGATTTTGGGATAACGATACTGATGATGATCTTAGTGCTGATGGGGAGGGTGATGCAATTGAGATTTAGGAGATTGATTGTAACCTTGTTTTAACTTTTTAATTATGTACTCTTAAAGGATTTTAATTTATTTTAAAAAACCAACCTGGTTTTTACTGATGTTCATAATGTTTCTTACTGTAGGTTGTCATGGTTGGTGGTGGTCGTCGGATGATGGGACATACCACCCCGAGAGAATTGTTTCCGTTAAGCCATGCAAATACGGAATCTTCCCCGGATGTTACCCCATCCCCGGAGTCACAGGTAGATGGTTCTGGGACATCTACTCAGGTTCCCGAGACGCAAGACGATAACTCTCCCATGGGTGAGGTATCTACCCCCGTTGCGAGAAGGACTTTGCACCCTACAGGGTTATGGTAAGTTAATCATGGTTAGCTTAATTTGCATTCCAACTTGAACAATCTTGTTTAGATTTGTATTTTATGTATGTAATGACTTCATTTGTTTGCTTATTGGTACTAAGGTTCGATGATAACACCGTGAGCACCTCCATTACTAGTATTTTTCAAGCACATTGGCGCAAACCATGGCTTAACTATGAAGAGGTTGACAAAGGCACTAAGACCCATTGGTGGAACCAGTTTACGGTATTATATAACCCCCAAGTTTAATTTACTTATCTAACTTTGGTGTTTGTTGGTTACTTATTACAATATATTATTAAACATATAATTTTTGTTGTTTCTTATAAAAATTTGCAGAAACAATACCTTTGGAATGCTGATCTTGATACCCTTGTGAAGAAGGAGTATGAAAAGAAGGCACATAAGAGGTTGAAAGAGATTACATACAATGCTTCTGTCAGAAACAAGTCAATTCCTAAATGGATGGGACAGTGTATGTTTCAGCAAATGATGGCGAAAAGAAAGGAGAAGGAATTTCTGGAGAGGTCAGAAAAAGCTAAGAAGAACAGGAGGGGCGGCTCCCTGTCTAATCCAATTGAGCCGTCACACTTCCAAGGTTCTATCTCTGCATCAAAGCATGCAAAGAAAATGGTAATACTTAATGGTAAAATGTTAAATTTTAGTAGTTACATAGATTATGCATTTAAGGTGTTATTTGATTTGTGCAGGCTCAGAAAGCTGGTGGAGTGCTTCCAACTGCCCCTGAGGTCTTCTTAAAGACCCATTTCAAGGAAGTTCCTGGAAAAGGAAAAGTTGCGGCTAACAGGAGGGCACAACAAATCTCGGTAACACATCTAACTTTGGAAACTAATGAAATAATTTTTTAAGGAAGAAAGCAACACATAGGCACTTATACTTGTGATTTGAAACTTTAGGCACCTAATACTTTTCAATATTTGTATGTTAGGTTCAATTTTAGTTTATAAAGAGACCAAAGAAAATGGTAAATATAGAACCTCATTTATGCCATTTTAGCCTAGTTATGACCCTATAGCCTTGTATAAGCATGAAAATGTCTATTATTAAGCCATTTCAACTTATAAACATGTTTAATATGCTAAATATGAGTCGTTTGATCGATAAAATCTTAGTTTATAGCGAAAACAAAGAAAATGGGTAGGTTAGAAGGTCATTGAAGCCATTTTAGCCTAGTTATGACCCTATAACCTTGTATAAGCATGAAAATGTCTATTATTAAGCCATTTCAACTTATAAACATGTTTAATATGCTAAATATGAGTCGTTTGATCGATAAAATCTTAGTTTATAGCGAAAACAAAGAAAATGGGTAGGTTAGAAGGTCATTGAAGCCATTTTAGCCTAGTTATGACCCTATAGCCTTGTATATGCATGAAATTGAATACTGTTAAGTAACTTCCACATATAATAGTGATAAAAACGCTAAATATGATTTTGTCATGATTACAGGATGCCTACCAAAAGAAGCTTGACGAGTCTTCCACTCAAGGGTCACAACCGGACCCTAACCAGCTATACTGGGAGGTCGTTGGTGGTAGGAAAAAGGGTGTAGTGAAGGGCCTAGGTGCAAGCGCTAGTTTGTTTTACAGCCCCTCTTCTGAGAAAGACTCGCAATCGTACAATCCTTCTATAGCTTCACAGTTGGAGGAACGTGTGCAAGCCGAGGTTGCCGAGCGAATAGAGGAGGCAAAAGTGCAGATGCAGGCACAGATGGAGGCACAGTTTGCTACGCAATTGCAGGAGGCATTGAAGAAGGAGCGGGAGGAGGCGGCAATTGAGAGTAAGCGAATGATGGAGACGATGTTGCAGAACTTGTTCTCTAACTGTTCAGGTGGTCCTTCATTTCAGCTTCGTAGGGAAGATGACCCGGACATGAGTGGTGGTGGGGTTGGTCACCAGACTCCGGTGTCGTAGTTTATTTGTTCATATAGCTCTTAGATTAGTATTGTTCATATAGGCCTTAGTTTTTTTTTGGGGAATATAGGCCTTAGCCTTTTTTTTTTGGGAATATAGGCCTTAGCTTTTTTTTAGGGGATATAGGCCTTAACTTAGTATTATATTGTTCATATTGGCCTTATCTATTTTTGAAAAAACACTGGCCATTACTTAGTATAATTCATATAGGCCTAACTTAGTTTTTAAATTTATCTTATAAAAATATTACTAACTAATTAGTATTTATATTTACTTGGACATTGTTATTGTAAGTATTGCTCAAAGTTGGATTCATTAATATGAATTATGCATTTTATTTTTAATTTTTATTATTTTTTAATTTAAATAATATGATAACAGGTTATAGGTTCAGGTTAGGATGGCATCGTAAATTCAAACGGTAATATTATGAAAATTGCATCCATTAACCTGAAATTTACGACCTTCCAATTTAAAATTTTGCGACCGTCTTGCTAGTCGCAGTCATACCAAAATTGCGACCGCCATACCATGTCGCAATTACAGAAAGATTGCGACGGTCACCTCCTGTCGCAAATATTCTTAATTGCGACCGTTATCTGCAGTCGCAATCCGCTGAAACTGCGACCGTTTTAACCCTGTCGCAATTTTTAGAATCTTGCGACCGTCATAGACCTGTCGCAATTATATCAATTTTGCGACCATCTACGACGGTTGTAATTTCAAAATATTGCGAGCGCAATACCTGTCGTAACTAACATATTACGTCTACACAAAAAACGGTCGTTGAAATTACTAAAAACGGTCGCAACAATATCTGCGACCGCCAAGTGGTCGTAAAATCTTTTTTTTGCGACCATTGGAATAACGACCGGTGCGTTGCGACGGGTGACGGTCGCAATTAGCTTTTGCGACCGTTTTCTATATGTTTTACGACCGTTTGTAGCGGTCGCAATTCACTGGTTTTTTAGTAGTGTTGTACATATCATGGCATACATGATTGAT

Dot-plot analysis:


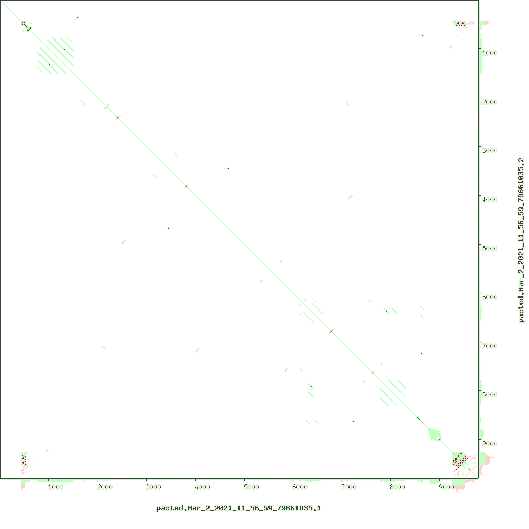


AUGUSTUS analysis:

**# coding sequence =** [gggaaaggggaaagaagaaacagagaagaagggggaagagggagaacaacgaagaggaggtgacggtcgcagactccgccgttcttccctacgacgagctatctccggccggcgacgctacacacaggaccaccgacgccggactcccctcctgcccgtccctcatttgtgttccgacggcggtactcaggcctaccgtcgccggtttcctcttctatcttagcctcctacgtttttcgtcgactaaactcaagcctaccgtcaccggtttcctctttgtcctagcctcctccgttttccgtcaactaaactctgaatttgtcaccatgaaaaggaaagagcgttggtggatgtatgatagatacgatggccgtaatgcccgtaaacttaggcaagagtttgtggatggtgttgaacaattcatacggttttgtgaagctcatcctgagagcccgggcatagatgaaataagatgcccttgtctgttatgtaagaatagacgtcttcatgatgccaacacagtaaaactccatttgtataaaaaggggtttatacctgattactatgagtgggtttctcatggtgaagggttccctacatcaagtgtagagcaaccaaatacttaccgtgaaatggttctcgatgctcttgggagtaatcatgaaccatcgggtgatgatagtactagtggttcagatgaagagccaaatgctcaagcaaaagcatttcaagagcttttaaaggccgccgagcaaccactgtacgaaggtagctccatgtctgtgctagaaatggcatctcgaattgtatctttgaagtcagaatacaatttgccccataggtgtgttgatgggtttgcttcattattgaacgaagcgattcccgacaataataagatggggaaaacattctatgatacaaagaagattcttaacggacttgagcttccgcacgagaagatccacgcatgcccaaagggttgtatgttattttggaaagctgatgctgagttggataagtgtagagtgtgcgaaagggacagatacaagaggacttcaaaggggtcattggttccaataaaagaactcatctattttcccatcactccaaggctacaacggttgtttgcaacaaagaacgttgcggaggaaatgacctggcattccaaaaatccaagagtccaaggcacaatggcgcatcctagtgatagtgaagcttggaagcacttggatagatccttccctgatttcgcatctgaacctagaaatgtgagacttggtatgtgtaccgatgggtttgctcctaatggcaagtttgggggtcagtactcatgttggccagtcattctgaccccttataacttgcctccatcgatgtgcatgaaaaaaccatttatgttcctttctttgctcgttcctggtcctaaaaatccgaaggggaacttggatgtgtatatgcaaccgttaattgaagaattgaaacaactgtgggatgttggtgcaacaacttacgacatttcgcaaagaaaaaacttcaatttgcgtgcagcaatcttgtggactataagtgatttcccagcctatggtatgctttctggatgggcaacagctggtaaaaaagcatgtccatattgcttggacaaatcaaaagctttttggcttgaacatggtggtaaaatgtcatggtttgattgtcaccgccaatttcttcctcctgaacatccttttcgaaacaacaaaattgctttttgtaagaataaggttgaaaaggggacaccgcctcacataatgtccggcgacgagctatggcaacaagttcaacacttggctaaggcaacagatggacctgaagcactcgcaagcttgaaaaagaagaaattagggtggtttaaacaaagcattctttgggagctaccgtactggaagactcttctcctccgccacaacctcgatgtcatgcatattgaaaagaatttctttgaccaactaattcacacagtgatggatgtgaaaaaacatacttcagacacagttgcatctagaaatgacattgccaaatattgcaaacgaccacaactacatgtgacggaggatgcaagagggaaagacactttgccgaaagccccattctcactagacaaggctcaaaagaaagtgttatgcgagtgggtaaggaacttgaaatttcccgatggttatgcttctaatttgagtaggtgtgtagatttacaaacatgtaagcttcatggtttaaagagccacgactgtcatgtcttcatggagagattgttgccagttgctttgaaggaattgctcccggtccatgtttggaaagctattactgagattagtcttttcttccgtgacctttgttgttccaccatcaaattgagtgatatggaaagattggaacacaacattgcagaaattctttgtaagctcgagaagatatttccaccagcgttcttcaactctatggaacacttaccagttcacctgccttatgaagctaggttatgtggtcctgtccaatatcgttggatgtatccgtttgaaaggttccttaatcatttgaaacgtaaaattggcaataaggctcgtgttgaaggttcaatctgcaatgcttatctaacggaagagatagggtacttttgctctaactacttccaacaaggagtggatactaaaacacgtgacttaggtcgtaatgttcatgatgatgttgaaagcactttggatgatagtgttccggaattgtttagggttgatcatggtcatgcttctactggtggagtgcgccgctttttggatgagaaagaactacaacgtgctcatttatatgttttgggaaatagcggcattctggaaccatatgagagggggtttgaggaccacattgttctaatgcaacccaatgttcgcaaagaagatgtgtggattaaacacgaagctgaattcctcgagtggtttaggttgaaagtgattgaagacaatccaaatgatgaaatattgtttgctttggcaatgggcccatctaagcgagtgcgtacttggagccaattctatgtgaacggatacaaattccaaacttatggccatggaaaacagaagtcaacgatgaattatggagtaagcgtatccaatgaagatggtggagattattttggtattttagaggatatcatccagttggagttcactggtgccctacggacttataaaactgttctcttcaagtgcagttggatggattctagtaggggtatgaacattgatcaatacaagcttgtagaagtcaatcacaccaaaaaataccccaagtacgacccatttgtgctttcttaccaagctaaccaagtctactacgcaccttacccaagtctgaaaagagataaggctcaatggtgggcagtgttcaagacaaaggcgaggtctgttgttgatgcaccagttgacgaagactttctacaagaaaccactgctgacattccaactctttgtgctccggatgacattccagactatgaaggtgatgaagatggatttggagattcggatactgatgatgaagtgctcccggatgcccctccagatgagtccagtttcgaatctactgatagtgaggacgaggatgaggatgacggattttgggatgacgatactgatgatgatcttagtgctgatggggaggtgctgatggggagggtgatgcaattgagatttaggagattgattgttgtcatggttggtggtggtcgtcggatgatgggacataccaccccgagagaattgtttccgttaagccatgcaaatacggaatcttccccggatgttaccccatccccggagtcacaggtagatggttctgggacatctactcaggttcccgagacgcaagacgataactctcccatgggtgaggtatctacccccgttgcgagaaggactttgcaccctacagggttatggttcgatgataacaccgtgagcacctccattactagtatttttcaagcacattggcgcaaaccatggcttaactatgaagaggttgacaaaggcactaagacccattggtggaaccagtttacgaaacaatacctttggaatgctgatcttgatacccttgtgaagaaggagtatgaaaagaaggcacataagaggttgaaagagattacatacaatgcttctgtcagaaacaagtcaattcctaaatggatgggacagtgtatgtttcagcaaatgatggcgaaaagaaaggagaaggaatttctggagaggtcagaaaaagctaagaagaacaggaggggcggctccctgtctaatccaattgagccgtcacacttccaaggttctatctctgcatcaaagcatgcaaagaaaatggctcagaaagctggtggagtgcttccaactgcccctgaggtcttcttaaagacccatttcaaggaagttcctggaaaaggaaaagttgcggctaacaggagggcacaacaaatctcggatgcctaccaaaagaagcttgacgagtcttccactcaagggtcacaaccggaccctaaccagctatactgggaggtcgttggtggtaggaaaaagggtgtagtgaagggcctaggtgcaagcgctagtttgttttacagcccctcttctgagaaagactcgcaatcgtacaatccttctatagcttcacagttggaggaacgtgtgcaagccgaggttgccgagcgaatagaggaggcaaaagtgcagatgcaggcacagatggaggcacagtttgctacgcaattgcaggaggcattgaagaaggagcgggaggaggcggcaattgagagtaagcgaatgatggagacgatgttgcagaacttgttctctaactgttcaggtggtccttcatttcagcttcgtagggaagatgacccggacatgagtggtggtggggttggtcaccagactccggtgtcgtag]

**# protein sequence =** [ERGKKKQRRRGKRENNEEEVTVADSAVLPYDELSPAGDATHRTTDAGLPSCPSLICVPTAVLRPTVAGFLFYLSLLRFSSTKLKPTVTGFLFVLASSVFRQLNSEFVTMKRKERWWMYDRYDGRNARKLRQEFVDGVEQFIRFCEAHPESPGIDEIRCPCLLCKNRRLHDANTVKLHLYKKGFIPDYYEWVSHGEGFPTSSVEQPNTYREMVLDALGSNHEPSGDDSTSGSDEEPNAQAKAFQELLKAAEQPLYEGSSMSVLEMASRIVSLKSEYNLPHRCVDGFASLLNEAIPDNNKMGKTFYDTKKILNGLELPHEKIHACPKGCMLFWKADAELDKCRVCERDRYKRTSKGSLVPIKELIYFPITPRLQRLFATKNVAEEMTWHSKNPRVQGTMAHPSDSEAWKHLDRSFPDFASEPRNVRLGMCTDGFAPNGKFGGQYSCWPVILTPYNLPPSMCMKKPFMFLSLLVPGPKNPKGNLDVYMQPLIEELKQLWDVGATTYDISQRKNFNLRAAILWTISDFPAYGMLSGWATAGKKACPYCLDKSKAFWLEHGGKMSWFDCHRQFLPPEHPFRNNKIAFCKNKVEKGTPPHIMSGDELWQQVQHLAKATDGPEALASLKKKKLGWFKQSILWELPYWKTLLLRHNLDVMHIEKNFFDQLIHTVMDVKKHTSDTVASRNDIAKYCKRPQLHVTEDARGKDTLPKAPFSLDKAQKKVLCEWVRNLKFPDGYASNLSRCVDLQTCKLHGLKSHDCHVFMERLLPVALKELLPVHVWKAITEISLFFRDLCCSTIKLSDMERLEHNIAEILCKLEKIFPPAFFNSMEHLPVHLPYEARLCGPVQYRWMYPFERFLNHLKRKIGNKARVEGSICNAYLTEEIGYFCSNYFQQGVDTKTRDLGRNVHDDVESTLDDSVPELFRVDHGHASTGGVRRFLDEKELQRAHLYVLGNSGILEPYERGFEDHIVLMQPNVRKEDVWIKHEAEFLEWFRLKVIEDNPNDEILFALAMGPSKRVRTWSQFYVNGYKFQTYGHGKQKSTMNYGVSVSNEDGGDYFGILEDIIQLEFTGALRTYKTVLFKCSWMDSSRGMNIDQYKLVEVNHTKKYPKYDPFVLSYQANQVYYAPYPSLKRDKAQWWAVFKTKARSVVDAPVDEDFLQETTADIPTLCAPDDIPDYEGDEDGFGDSDTDDEVLPDAPPDESSFESTDSEDEDEDDGFWDDDTDDDLSADGEVLMGRVMQLRFRRLIVVMVGGGRRMMGHTTPRELFPLSHANTESSPDVTPSPESQVDGSGTSTQVPETQDDNSPMGEVSTPVARRTLHPTGLWFDDNTVSTSITSIFQAHWRKPWLNYEEVDKGTKTHWWNQFTKQYLWNADLDTLVKKEYEKKAHKRLKEITYNASVRNKSIPKWMGQCMFQQMMAKRKEKEFLERSEKAKKNRRGGSLSNPIEPSHFQGSISASKHAKKMAQKAGGVLPTAPEVFLKTHFKEVPGKGKVAANRRAQQISDAYQKKLDESSTQGSQPDPNQLYWEVVGGRKKGVVKGLGASASLFYSPSSEKDSQSYNPSIASQLEERVQAEVAERIEEAKVQMQAQMEAQFATQLQEALKKEREEAAIESKRMMETMLQNLFSNCSGGPSFQLRREDDPDMSGGGVGHQTPVS]

# end gene g1

###

*Batch* analysis:


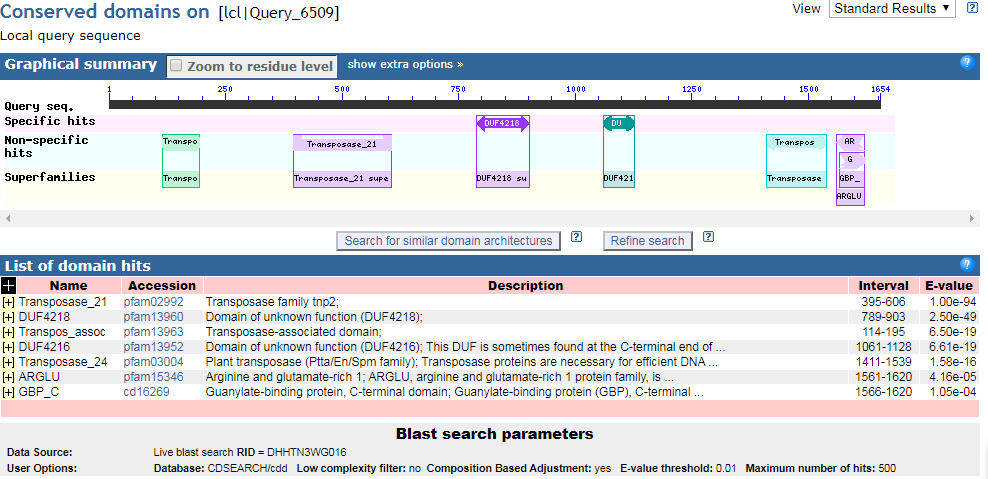


Analysis of Clone Tp24-X1 (GenBank # MZ325224) from *Chenopodium iljinii* genome (the clone fragments overlaps are highlighted in yellow):

>F1

TAGATACGATGGCCGTAATGCCCGTAAACTTAGGCAAGAGTTTGTGGATGGTGTTGAACAATTCATACGATTTTGTGAAGCTCATCCTGAGAGCCCGGGCATAGATGAAATAAGATGCCCTTGTCTGTTATGTAAGAATAGACGTCTTCATGATGCCAACACAGTAAAACTCCATTTGTATATAAAGGGGTATATACCTGATTACTATGAGTGGGTTTCTCATGGTGAAGGGTTCCCTACATCAAGTGTAGAGCAACCAAATACTTACCGTGAAATGGTTCTCGATGCTCTTGGGAGTAATCATGAACCATCGGGTGATGATAGTACTAGTGGTTCAGATGAAGAGCCAAATGCTCAAGCAAAAGCATTTCAAGAGCTTTTAAAGGCCGCCGAGCAACCACTGTACGAAGGTAGCTCCATGTCTGTGCTAGAAATGGCATCTCGAATTGTATCTTTGAAGTCAGAATACAATTTGCCCCATAGGTGTGTTGATGGGTTTGCTTCATTATTGAACGAAGCGATTCCCGACAATAATAAGATGGGGAAAACATTCTATGATACAAAGAAGATTCTTAACGGACTTGAGCTTCCGCACGAGAAGATCCACGCATGCCCAAAGGGTTGTATGTTATTTTGGAAAGCTGATGCTGAGTTGGATAAGTGTAGAGTGTGCGAAAGGGACAGATACAAGAGGACTTCAAAGGGGTCATTGGTTCCAATAAAAGAACTCATCTATTTTCCTATCACTCCAAGGCTACAACGGTTGTTTGCAACAAAGAACGTTGCGGAGGAAATGACCTGGCATTCCAAAAATCCAAGAGTCCAAGGCACAATGGCGCATCCTAGTGATAGTGAAGCTTGGAAGCACTTGGATAGATCCTTCCCTGATTTTGCATCTGAACCTAGAAATGTGAGACTTGGTATGTGTACCGATGGGTTTGCTCCTTATGGCAAGTTTGGGGGTCAGTACTCATGTTGGCCAGTCATTCTGACCCCTTATAACTTGCCTCCATCGATGTGCATGAAAAAACCATTTATGTTCCTT

>F2

CATTTATGTTCCTTTCTTTGCTCGTTCCTGGTCCTAAAATCCGAAAGGGAACTTGAATGTGTATATGCAACCGTTAATTGAAGAATTGAAACAACTGTGGGATGTTGGTGCAACAACTTACGACATTTCGCAAAGAAAAAACTTCAATTTGTGTGCGGCAATCTTGTGGACTATAAGTGATTTCCCAGCCTATGGTATGCTTTCTGGATGGGCAACAGCTGGTTAAAAAAGCATGTCCATATTGCTTGGACAAATCAAAAGCTTTTTTGCTTGAACATGGTGGTAAAATATCATGGTTTGATTGTCACCGCCAATTTCTTCCTCCTGAACATCCTTTTCGAAACAACAAAATTGCTTTTTGTAAGAATAAGGTTGAAAAGGGGACACCGCCTCACATAATGTCCGGCGACGAGCTATGGCAACAAGTTCAACACTTGGCTAAGGCAACAGATGGACCTGAAGCACTCGCAAGCTTGAAAAAGAAGAAATTAGGGTGGTTTAAACAAAGCATTCTTTGGGAGCTACCGTACTGGAAAACTCTTCTCCTCCGCCACAATCTCGATGTCATGCATATTGAAAAGAATTTCTTTGACCAACTAATTCACACAGTGATGGACGTGAAAAAACGTACTTCAGACACAGTTGCATCTAGAAATGACATTGCCAAATATTGCAAACGACCACAACTACATGTGACGGAGGATGCGAGAGGGAAAGACACTTTGCCGAAAGCCCCATTCTCACTAGACAAGGCTCAAAATAAAGTGTTATGCGAGTGGGTAAGGAACTTGAAATTTCCCGATGGTTATGCTTATAATTTGAGTAGGTGTGTAGATTTACAAACATGTAAGCTTCATGGTTTAAAGAGCCACGACTGTCATGTCTTCATAGAGAGATTGTTGCTAGTTGCTTTGAAGGAATTGCTTCCATGTTTGGAAAGCTATTACTGAGATTAGTCTTTTCTTCCGTGACCTTTGTTGTTCCACCATAAAATTGAGTGATATGGAAAGATTGGAACACAACATTGTAGAAATTCTTTGTAAGCT

>F3

TTCTTTGTAAGCTCGAGAAGATATTTCCACCAGCGTTCTTCAACTCTATGGAACACTTACCAGTTCATCTGCCTTATGAAGCTAGGTTATGTGGTCCTGTCCAATATCGTTGGATGTATCCGTTTGAAAGGTTTTAGCTCCTACAAACTCATAGATGCCATTTTCGTTTATACACCTATAGTTATTTGCATAACATATAACTTATTCAATATTTTTATTTATAGGTTCCTTAATCATTTGAAACGTAAAATTGGCAATAAGGCTCGTGTTGAAGGTTCAATCTGCAATGCTTATCTAACAGAAGAGATAGGGTACTTTTGCTCTAACTACTTCCAACAAGGAGTGGATACTAAAACACGTGACTTAGGTCGTAATGTTCATGATGATGTTGAAAGCACTTTGGATGATAGTGTTCCGGAACTGTTTAGGGTTGATCATGGACATGCTTCTACTGGTGGAGTGCGCCGCTTTTTGGATGAGAAAGAACTACAACATGCTCATTTATATGTTTTGGGAAATAGCGGCATTCTGGAACCATATGAGAGGTATTAAATTTGTCTTTATCACACTCACTAAGATTTCATTGAATTTTGCAACGTATCCAACGTCCACCACCATCATTAATACTCTTTCTCTGGTTATACGGAAGGGGGTTTGAGGACCACATTGTTCTAATGCAACCCACTATTCGCAAAGAAGATGTGTGGATTAAACATGAAGCTGAATTCCTCGAGTGGTTTAGGTTGAAAGTGAGTGTGTTACGCATTTGACATCAACACGTTTTCTTACTTTGTTGCTATAACTTCACGA

**From the reverse side:**

>R3

TATAACTTCACGACATATATCAATTTAATATAATTATGCTATATGAGCAGGTGATTCAAGACATGCCAAATGATGAAATATTGTTTGCTTTGGCTATGGGCCCATCTAAGCGAGTGCGTACTTGGAGCCAATTCTATGTGAACGGATACATATTCCAAACTTATGGCCATGGAAAACAGAAGTCAACGATAAATTATGGAGTAAGTGTATCCAATGAAGATGGTGGAGATTATTTTGGTATCTTAGAGGATATCATCGAGTTGGAGTTCACTGGTGCCCTATGGAGTTATAAGACTATTCTCTTCAAGTGCAGTTGGATGGATTCTAGTAGGGGTATGAACATTGATCAATACAAGCTTGTAGAAGTCAATCACACCAAAAAATACCCCAAGTACGATCCATTTATGCTTTCTTACCAAGCTAACCAAGTCTACTATGCACCTTACCCAAGTCTGAAAAGAGATAAGGCTCGATGGTGGGCAGTGTTCGAGACGAAGGCGAGGTCTGTTGTTGATGCACCAGTTGGCGAAGACTTTCTATAAGAAACCACTGCTGACATTCCAATTCTTTGTGCTCCGGATGACATTCCAGACTATGAAGGTGATGAATATGGATTTGGAGATTCGGATACTGATGATGAAGTGCTCCCGGATGCCCCTCCAGATG

>R2

CCCTCCAGATGAGTCCAGTTTCGAATCTACTGATAGTGAGGACGAGGATGAGGATGACGGATTTTAGGATGACGATACTGATGATGATCTTAGTGCTGATGGGGAGGGTGATTCAACTGAGATTTAGGAAATTGATTGTAACCTTGTTTTAACTTTTTAATTATGTAATCTTAATGGATTTCAATTTATTTTAAAACACCATCTTGGTTTTATTGATGTTCATAATGTTTCTTACTGTAGGTTGTCATGGTTGGTGGTGGTCGTCGGATGCTAGGACATGCCACCCCGAGAGAATTTTTTCAGTCAACAAGCCATGCAAATACGGAATCTTCCCCAGAAGTTACCCCATCCCCAGAGTCACAAGTAGATGGTTCTGGGACATCTACTCAGGTTCCTGAGACGCAAGACGATAACTCTCCCATGGGTGAGGTAGCTACCCCCGTTGCGAGAAGGACTTTGCACCCTATAGGGTTATGGTAAGTTAATCATGGTTAGCTTAATTTGCATTCCAACTTGAACAATCTTGGTTAGATTTGTATTTTATGTACGTAATAACTTCATTTGTTTGCTTATTGGTACTAAGGTTCAATGATAACACCGTGAGCACCTTCATTACTAGTATTTTTCAAGCACATTGGCGCAAACCATGGCTTAACTATGAAGAGGTTGACAAAGGCACTAAGACCCATTGGTGGAACCAGTTTACGGTATTATATAACCCCCAAGTTTAATTTACTTATCTAACTTAGGTGTTTGTTGGTTACTTATTGCAATAAATTATTAAACATATAATTTTTGTTGTTTCTTATAAAAATTTGCAGAAAAAATACCTTTGGAATGTTGATCTTGATACCCTTGTGAAGAGGAGTATGAAAAGAAGGCACATAAGAGGTTGAAAGAGATTACATATAATGCTTCTGTCAGAAACAAGTCAATTCCTAAATGGACGGGACAGTGTATGTTTTAGCAAATGATGGCGAAAAGAAAGGAGAAGGAATTTTTGGAGAGGTCAGAAAAAGCTAAGAAGAACAGGAGGGGCGGCTCCTTGTCTAATCCAATTGAGCCGTCACACTTCCAA

>R1

TTCCAAGGTTCTATTCTGCATCAAAGCATGCAAAGAAAATGGTAATACTTAATGGTAAATGATAATTTTAGTAGTTACATCCACTAAGCATTTAAGGAGTTATTTGATTGTGCAGGCTCAGAAAGCCGGTGGAGTGCTTCCAACTGCCCCTGAGGTCTTCCTAAAGACCCATTTCAAGGAAGTTCCTAGAAAAGGAAAAGTTGCGGCTAACAAGAGGGCACAACAAATCTCGGTAACACATCTAACTTTGGAAACTAATGAAATAATTTTGTAAGGAAGAAAGCAAAACATAGGCACTTATACTTGTGATTTAAAACTTTAGGGCACCTAATACTTTTCAATATTGGTATGTTAGGTTCAATTTTAGATTATAAAGAGAACAAAGAAAATGGTAAATATAGAACCTCATTTATGCCATTTTCTCCTATGTATGACCCTATAGCCTTGTATTTGCATGAAAATGTGTATTATTTAGAAATTTTTACCTACAATAATGTAATATGGGGAATATGAGTTGTGTGATTGATAAAATCTTAGAATATAGCAAAAACAAAGGAAATGGATAAGTTAGAAGGTCATTTAAGCCATTTTAGCCTAGTTATGACCCTATATCCTTGTATATGCATGAAAATGTCTATTATTAAGCCATTTCAACCTATAAACATGTTTAATATGCTAAATATGAGTTGTTTGATTGATAAAATATTAGAATATAGCAAAAACAAACAAAATGGGTAAGTTAGTAGGTCATTTAAGCCATTTTAGCCTAGTTATGATCCTATAGCCTTGTACATGCATGAAAATGTCTATTATTAAGCCATTTCAACCTATAAACATGTTTAATATGCTGAATATGAGTTGTTTGATTGATAAAATCTTAGTTTATAGTGAAAACAAAGAAAATGGGTATGTTAGAAGGTCATTTAAGCCATTTAAGCCAAATTATGACCTTGTAGCCTTGTATTTGCATGAAATTGAATACTTTTAAGCAACTTCCACATACAATAGTGATAAATATACTGAATGTGATTTGTCCTGATTACAGGATGCCTACCAAAAGAATCTTGACGAGTCTCCCACTCAAGGGTCACAACCGGACCCTAACCAGCTATATTGGGAGGTCGTTGGTGGTAGGAAGGGCGAATTCGTTTAAACCTGCAGGACTAGTCCCTTTAGTGAGGGTTAATTT

Combined sequence of Tp24-X1clone from clone fragments:

TAGATACGATGGCCGTAATGCCCGTAAACTTAGGCAAGAGTTTGTGGATGGTGTTGAACAATTCATACGATTTTGTGAAGCTCATCCTGAGAGCCCGGGCATAGATGAAATAAGATGCCCTTGTCTGTTATGTAAGAATAGACGTCTTCATGATGCCAACACAGTAAAACTCCATTTGTATATAAAGGGGTATATACCTGATTACTATGAGTGGGTTTCTCATGGTGAAGGGTTCCCTACATCAAGTGTAGAGCAACCAAATACTTACCGTGAAATGGTTCTCGATGCTCTTGGGAGTAATCATGAACCATCGGGTGATGATAGTACTAGTGGTTCAGATGAAGAGCCAAATGCTCAAGCAAAAGCATTTCAAGAGCTTTTAAAGGCCGCCGAGCAACCACTGTACGAAGGTAGCTCCATGTCTGTGCTAGAAATGGCATCTCGAATTGTATCTTTGAAGTCAGAATACAATTTGCCCCATAGGTGTGTTGATGGGTTTGCTTCATTATTGAACGAAGCGATTCCCGACAATAATAAGATGGGGAAAACATTCTATGATACAAAGAAGATTCTTAACGGACTTGAGCTTCCGCACGAGAAGATCCACGCATGCCCAAAGGGTTGTATGTTATTTTGGAAAGCTGATGCTGAGTTGGATAAGTGTAGAGTGTGCGAAAGGGACAGATACAAGAGGACTTCAAAGGGGTCATTGGTTCCAATAAAAGAACTCATCTATTTTCCTATCACTCCAAGGCTACAACGGTTGTTTGCAACAAAGAACGTTGCGGAGGAAATGACCTGGCATTCCAAAAATCCAAGAGTCCAAGGCACAATGGCGCATCCTAGTGATAGTGAAGCTTGGAAGCACTTGGATAGATCCTTCCCTGATTTTGCATCTGAACCTAGAAATGTGAGACTTGGTATGTGTACCGATGGGTTTGCTCCTTATGGCAAGTTTGGGGGTCAGTACTCATGTTGGCCAGTCATTCTGACCCCTTATAACTTGCCTCCATCGATGTGCATGAAAAAACCATTTATGTTCCTTTCTTTGCTCGTTCCTGGTCCTAAAATCCGAAAGGGAACTTGAATGTGTATATGCAACCGTTAATTGAAGAATTGAAACAACTGTGGGATGTTGGTGCAACAACTTACGACATTTCGCAAAGAAAAAACTTCAATTTGTGTGCGGCAATCTTGTGGACTATAAGTGATTTCCCAGCCTATGGTATGCTTTCTGGATGGGCAACAGCTGGTTAAAAAAGCATGTCCATATTGCTTGGACAAATCAAAAGCTTTTTTGCTTGAACATGGTGGTAAAATATCATGGTTTGATTGTCACCGCCAATTTCTTCCTCCTGAACATCCTTTTCGAAACAACAAAATTGCTTTTTGTAAGAATAAGGTTGAAAAGGGGACACCGCCTCACATAATGTCCGGCGACGAGCTATGGCAACAAGTTCAACACTTGGCTAAGGCAACAGATGGACCTGAAGCACTCGCAAGCTTGAAAAAGAAGAAATTAGGGTGGTTTAAACAAAGCATTCTTTGGGAGCTACCGTACTGGAAAACTCTTCTCCTCCGCCACAATCTCGATGTCATGCATATTGAAAAGAATTTCTTTGACCAACTAATTCACACAGTGATGGACGTGAAAAAACGTACTTCAGACACAGTTGCATCTAGAAATGACATTGCCAAATATTGCAAACGACCACAACTACATGTGACGGAGGATGCGAGAGGGAAAGACACTTTGCCGAAAGCCCCATTCTCACTAGACAAGGCTCAAAATAAAGTGTTATGCGAGTGGGTAAGGAACTTGAAATTTCCCGATGGTTATGCTTATAATTTGAGTAGGTGTGTAGATTTACAAACATGTAAGCTTCATGGTTTAAAGAGCCACGACTGTCATGTCTTCATAGAGAGATTGTTGCTAGTTGCTTTGAAGGAATTGCTTCCATGTTTGGAAAGCTATTACTGAGATTAGTCTTTTCTTCCGTGACCTTTGTTGTTCCACCATAAAATTGAGTGATATGGAAAGATTGGAACACAACATTGTAGAAATTCTTTGTAAGCTCGAGAAGATATTTCCACCAGCGTTCTTCAACTCTATGGAACACTTACCAGTTCATCTGCCTTATGAAGCTAGGTTATGTGGTCCTGTCCAATATCGTTGGATGTATCCGTTTGAAAGGTTTTAGCTCCTACAAACTCATAGATGCCATTTTCGTTTATACACCTATAGTTATTTGCATAACATATAACTTATTCAATATTTTTATTTATAGGTTCCTTAATCATTTGAAACGTAAAATTGGCAATAAGGCTCGTGTTGAAGGTTCAATCTGCAATGCTTATCTAACAGAAGAGATAGGGTACTTTTGCTCTAACTACTTCCAACAAGGAGTGGATACTAAAACACGTGACTTAGGTCGTAATGTTCATGATGATGTTGAAAGCACTTTGGATGATAGTGTTCCGGAACTGTTTAGGGTTGATCATGGACATGCTTCTACTGGTGGAGTGCGCCGCTTTTTGGATGAGAAAGAACTACAACATGCTCATTTATATGTTTTGGGAAATAGCGGCATTCTGGAACCATATGAGAGGTATTAAATTTGTCTTTATCACACTCACTAAGATTTCATTGAATTTTGCAACGTATCCAACGTCCACCACCATCATTAATACTCTTTCTCTGGTTATACGGAAGGGGGTTTGAGGACCACATTGTTCTAATGCAACCCACTATTCGCAAAGAAGATGTGTGGATTAAACATGAAGCTGAATTCCTCGAGTGGTTTAGGTTGAAAGTGAGTGTGTTACGCATTTGACATCAACACGTTTTCTTACTTTGTTGCTATAACTTCACGAACATATATCAATTTAATATAATTATGCTATATGAGCAGGTGATTCAAGACATGCCAAATGATGAAATATTGTTTGCTTTGGCTATGGGCCCATCTAAGCGAGTGCGTACTTGGAGCCAATTCTATGTGAACGGATACATATTCCAAACTTATGGCCATGGAAAACAGAAGTCAACGATAAATTATGGAGTAAGTGTATCCAATGAAGATGGTGGAGATTATTTTGGTATCTTAGAGGATATCATCGAGTTGGAGTTCACTGGTGCCCTATGGAGTTATAAGACTATTCTCTTCAAGTGCAGTTGGATGGATTCTAGTAGGGGTATGAACATTGATCAATACAAGCTTGTAGAAGTCAATCACACCAAAAAATACCCCAAGTACGATCCATTTATGCTTTCTTACCAAGCTAACCAAGTCTACTATGCACCTTACCCAAGTCTGAAAAGAGATAAGGCTCGATGGTGGGCAGTGTTCGAGACGAAGGCGAGGTCTGTTGTTGATGCACCAGTTGGCGAAGACTTTCTATAAGAAACCACTGCTGACATTCCAATTCTTTGTGCTCCGGATGACATTCCAGACTATGAAGGTGATGAATATGGATTTGGAGATTCGGATACTGATGATGAAGTGCTCCCGGATGCCCCTCCAGATGAGTCCAGTTTCGAATCTACTGATAGTGAGGACGAGGATGAGGATGACGGATTTTAGGATGACGATACTGATGATGATCTTAGTGCTGATGGGGAGGGTGATTCAACTGAGATTTAGGAAATTGATTGTAACCTTGTTTTAACTTTTTAATTATGTAATCTTAATGGATTTCAATTTATTTTAAAACACCATCTTGGTTTTATTGATGTTCATAATGTTTCTTACTGTAGGTTGTCATGGTTGGTGGTGGTCGTCGGATGCTAGGACATGCCACCCCGAGAGAATTTTTTCAGTCAACAAGCCATGCAAATACGGAATCTTCCCCAGAAGTTACCCCATCCCCAGAGTCACAAGTAGATGGTTCTGGGACATCTACTCAGGTTCCTGAGACGCAAGACGATAACTCTCCCATGGGTGAGGTAGCTACCCCCGTTGCGAGAAGGACTTTGCACCCTATAGGGTTATGGTAAGTTAATCATGGTTAGCTTAATTTGCATTCCAACTTGAACAATCTTGGTTAGATTTGTATTTTATGTACGTAATAACTTCATTTGTTTGCTTATTGGTACTAAGGTTCAATGATAACACCGTGAGCACCTTCATTACTAGTATTTTTCAAGCACATTGGCGCAAACCATGGCTTAACTATGAAGAGGTTGACAAAGGCACTAAGACCCATTGGTGGAACCAGTTTACGGTATTATATAACCCCCAAGTTTAATTTACTTATCTAACTTAGGTGTTTGTTGGTTACTTATTGCAATAAATTATTAAACATATAATTTTTGTTGTTTCTTATAAAAATTTGCAGAAAAAATACCTTTGGAATGTTGATCTTGATACCCTTGTGAAGAGGAGTATGAAAAGAAGGCACATAAGAGGTTGAAAGAGATTACATATAATGCTTCTGTCAGAAACAAGTCAATTCCTAAATGGACGGGACAGTGTATGTTTTAGCAAATGATGGCGAAAAGAAAGGAGAAGGAATTTTTGGAGAGGTCAGAAAAAGCTAAGAAGAACAGGAGGGGCGGCTCCTTGTCTAATCCAATTGAGCCGTCACACTTCCAAGGTTCTATTCTGCATCAAAGCATGCAAAGAAAATGGTAATACTTAATGGTAAATGATAATTTTAGTAGTTACATCCACTAAGCATTTAAGGAGTTATTTGATTGTGCAGGCTCAGAAAGCCGGTGGAGTGCTTCCAACTGCCCCTGAGGTCTTCCTAAAGACCCATTTCAAGGAAGTTCCTAGAAAAGGAAAAGTTGCGGCTAACAAGAGGGCACAACAAATCTCGGTAACACATCTAACTTTGGAAACTAATGAAATAATTTTGTAAGGAAGAAAGCAAAACATAGGCACTTATACTTGTGATTTAAAACTTTAGGGCACCTAATACTTTTCAATATTGGTATGTTAGGTTCAATTTTAGATTATAAAGAGAACAAAGAAAATGGTAAATATAGAACCTCATTTATGCCATTTTCTCCTATGTATGACCCTATAGCCTTGTATTTGCATGAAAATGTGTATTATTTAGAAATTTTTACCTACAATAATGTAATATGGGGAATATGAGTTGTGTGATTGATAAAATCTTAGAATATAGCAAAAACAAAGGAAATGGATAAGTTAGAAGGTCATTTAAGCCATTTTAGCCTAGTTATGACCCTATATCCTTGTATATGCATGAAAATGTCTATTATTAAGCCATTTCAACCTATAAACATGTTTAATATGCTAAATATGAGTTGTTTGATTGATAAAATATTAGAATATAGCAAAAACAAACAAAATGGGTAAGTTAGTAGGTCATTTAAGCCATTTTAGCCTAGTTATGATCCTATAGCCTTGTACATGCATGAAAATGTCTATTATTAAGCCATTTCAACCTATAAACATGTTTAATATGCTGAATATGAGTTGTTTGATTGATAAAATCTTAGTTTATAGTGAAAACAAAGAAAATGGGTATGTTAGAAGGTCATTTAAGCCATTTAAGCCAAATTATGACCTTGTAGCCTTGTATTTGCATGAAATTGAATACTTTTAAGCAACTTCCACATACAATAGTGATAAATATACTGAATGTGATTTGTCCTGATTACAGGATGCCTACCAAAAGAATCTTGACGAGTCTCCCACTCAAGGGTCACAACCGGACCCTAACCAGCTATATTGGGAGGTCGTTGGTGGTAGGAAGGGCGAATTCGTTTAAACCTGCAGGACTAGTCCCTTTAGTGAGGGTTAATTT

AUGUSTUS analysis:

# coding sequence = [tagatacgatggccgtaatgcccgtaaacttaggcaagagtttgtggatggtgttgaacaattcatacgattttgtgaagctcatcctgagagcccgggcatagatgaaataagatgcccttgtctgttatgtaagaatagacgtcttcatgatgccaacacagtaaaactccatttgtatataaaggggtatatacctgattactatgagtgggtttctcatggtgaagggttccctacatcaagtgtagagcaaccaaatacttaccgtgaaatggttctcgatgctcttgggagtaatcatgaaccatcgggtgatgatagtactagtggttcagatgaagagccaaatgctcaagcaaaagcatttcaagagcttttaaaggccgccgagcaaccactgtacgaaggtagctccatgtctgtgctagaaatggcatctcgaattgtatctttgaagtcagaatacaatttgccccataggtgtgttgatgggtttgcttcattattgaacgaagcgattcccgacaataataagatggggaaaacattctatgatacaaagaagattcttaacggacttgagcttccgcacgagaagatccacgcatgcccaaagggttgtatgttattttggaaagctgatgctgagttggataagtgtagagtgtgcgaaagggacagatacaagaggacttcaaaggggtcattggttccaataaaagaactcatctattttcctatcactccaaggctacaacggttgtttgcaacaaagaacgttgcggaggaaatgacctggcattccaaaaatccaagagtccaaggcacaatggcgcatcctagtgatagtgaagcttggaagcacttggatagatccttccctgattttgcatctgaacctagaaatgtgagacttggtatgtgtaccgatgggtttgctccttatggcaagtttggggtgatttcccagcctatggtatgctttctggatgggcaacagctggttaaaaaagcatgtccatattgcttggacaaatcaaaagcttttttgcttgaacatggtggtaaaatatcatggtttgattgtcaccgccaatttcttcctcctgaacatccttttcgaaacaacaaaattgctttttgtaagaataaggttgaaaaggggacaccgcctcacataatgtccggcgacgagctatggcaacaagttcaacacttggctaaggcaacagatggacctgaagcactcgcaagcttgaaaaagaagaaattagggtggtttaaacaaagcattctttgggagctaccgtactggaaaactcttctcctccgccacaatctcgatgtcatgcatattgaaaagaatttctttgaccaactaattcacacagtgatggacgtgaaaaaacgtacttcagacacagttgcatctagaaatgacattgccaaatattgcaaacgaccacaactacatgtgacggaggatgcgagagggaaagacactttgccgaaagccccattctcactagacaaggctcaaaataaagtgttatgcgagtgggtaaggaacttgaaatttcccgatggttatgcttataatttgagtaggaattgcttccatgtttggaaagctattactgagattagtcttttcttccgtgacctttgttgttccaccataaaattgagtgatatggaaagattggaacacaacattgtagaaattctttgtaagctcgagaagatatttccaccagcgttcttcaactctatggaacacttaccagttcatctgccttatgaagctaggttatgtggtcctgtccaatatcgttggatgtatccgtttgaaaggttccttaatcatttgaaacgtaaaattggcaataaggctcgtgttgaaggttcaatctgcaatgcttatctaacagaagagatagggtacttttgctctaactacttccaacaaggagtggatactaaaacacgtgacttaggtcgtaatgttcatgatgatgttgaaagcactttggatgatagtgttccggaactgtttagggttgatcatggacatgcttctactggtggagtgcgccgctttttggatgagaaagaactacaacatgctcatttatatgttttgggaaatagcggcattctggaaccatatgagagggggtttgaggaccacattgttctaatgcaacccactattcgcaaagaagatgtgtggattaaacatgaagctgaattcctcgagtggtttaggttgaaagtgattcaagacatgccaaatgatgaaatattgtttgctttggctatgggcccatctaagcgagtgcgtacttggagccaattctatgtgaacggatacatattccaaacttatggccatggaaaacagaagtcaacgataaattatggagtaagtgtatccaatgaagatggtggagattattttggtatcttagaggatatcatcgagttggagttcactggtgccctatggagttataagactattctcttcaagtgcagttggatggattctagtaggggtatgaacattgatcaatacaagcttgtagaagtcaatcacaccaaaaaataccccaagtacgatccatttatgctttcttaccaagctaaccaagtctactatgcaccttacccaagtctgaaaagagataaggctcgatggtgggcagtgttcgagacgaaggcgaggtctactatgaaggtgatgaatatggatttggagattcggatactgatgatgaagtgctcccggatgcccctccagatgagtccagtttcgaatctactgatagtgaggacgaggatgaggatgacggattttaggatgacgatactgatgatgatcttagtgctgatggggagggttgtcatggttggtggtggtcgtcggatgctaggacatgccaccccgagagaattttttcagtcaacaagccatgcaaatacggaatcttccccagaagttaccccatccccagagtcacaagtagatggttctgggacatctactcaggttcctgagacgcaagacgataactctcccatgggtgaggtagctacccccgttgcgagaaggactttgcaccctatagggttatggttcaatgataacaccgtgagcaccttcattactagtatttttcaagcacattggcgcaaaccatggcttaactatgaagaggttgacaaaggcactaagacccattggtggaaccagtttacggctcagaaagccggtggagtgcttccaactgcccctgaggtcttcctaaagacccatttcaaggaagttcctagaaaaggaaaagttgcggctaacaagagggcacaacaaatctcggatgcctaccaaaagaatcttgacgagtctcccactcaagggtcacaaccggaccctaaccagctatattgggaggtcgttggtggtaggaagggcgaattcgtttaa]

# protein sequence = [RYDGRNARKLRQEFVDGVEQFIRFCEAHPESPGIDEIRCPCLLCKNRRLHDANTVKLHLYIKGYIPDYYEWVSHGEGFPTSSVEQPNTYREMVLDALGSNHEPSGDDSTSGSDEEPNAQAKAFQELLKAAEQPLYEGSSMSVLEMASRIVSLKSEYNLPHRCVDGFASLLNEAIPDNNKMGKTFYDTKKILNGLELPHEKIHACPKGCMLFWKADAELDKCRVCERDRYKRTSKGSLVPIKELIYFPITPRLQRLFATKNVAEEMTWHSKNPRVQGTMAHPSDSEAWKHLDRSFPDFASEPRNVRLGMCTDGFAPYGKFGVISQPMVCFLDGQQLVKKACPYCLDKSKAFLLEHGGKISWFDCHRQFLPPEHPFRNNKIAFCKNKVEKGTPPHIMSGDELWQQVQHLAKATDGPEALASLKKKKLGWFKQSILWELPYWKTLLLRHNLDVMHIEKNFFDQLIHTVMDVKKRTSDTVASRNDIAKYCKRPQLHVTEDARGKDTLPKAPFSLDKAQNKVLCEWVRNLKFPDGYAYNLSRNCFHVWKAITEISLFFRDLCCSTIKLSDMERLEHNIVEILCKLEKIFPPAFFNSMEHLPVHLPYEARLCGPVQYRWMYPFERFLNHLKRKIGNKARVEGSICNAYLTEEIGYFCSNYFQQGVDTKTRDLGRNVHDDVESTLDDSVPELFRVDHGHASTGGVRRFLDEKELQHAHLYVLGNSGILEPYERGFEDHIVLMQPTIRKEDVWIKHEAEFLEWFRLKVIQDMPNDEILFALAMGPSKRVRTWSQFYVNGYIFQTYGHGKQKSTINYGVSVSNEDGGDYFGILEDIIELEFTGALWSYKTILFKCSWMDSSRGMNIDQYKLVEVNHTKKYPKYDPFMLSYQANQVYYAPYPSLKRDKARWWAVFETKARSTMKVMNMDLEIRILMMKCSRMPLQMSPVSNLLIVRTRMRMTDFRMTILMMILVLMGRVVMVGGGRRMLGHATPREFFQSTSHANTESSPEVTPSPESQVDGSGTSTQVPETQDDNSPMGEVATPVARRTLHPIGLWFNDNTVSTFITSIFQAHWRKPWLNYEEVDKGTKTHWWNQFTAQKAGGVLPTAPEVFLKTHFKEVPRKGKVAANKRAQQISDAYQKNLDESPTQGSQPDPNQLYWEVVGGRKGEFV]

# end gene g1

###

*Batch* analysis:

**
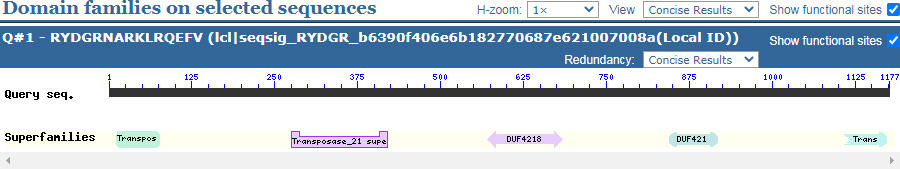
**

Analysis of the assembled contig 14 (*C. iljinii 2*):

>Cilj_assembled_contig14_iljinii_2

CACTAGTAGAAAAATGTACATTTGCGTCGCCATATTTGCGTCACTCATTCCAAAGAACGACGCAAATGTTTGTTTTTACCATCCTTAAAAGTTATTTGCGTCACACTTTATTCAAGAAGCGATGCAAAAGTCAGATGTATTTCAGCTTTCTATTATTTGCGTCGCTCTTATTTATTAAAGCGACGCATATAATAATCTTATTTGCGTCACTTATTTATTGACAAGTGACGCAAATGAGTGAGTGAATAAATTGCGTCACGCTTTTAATAAATGAGCGACGCATATGAAAATGTACATCATTATACTTTTTGTTATTTGCGTCGCATTTTAATAATTGAGTGACGCAAATAACTCAATTGCATTTCAGAATTTTTGACCTTAGCTTTTCACGCCTACCTTTCACTAAACCAAAAGAAAAAAAACAAGAGCATCGCTACTCCATTGAATTTCTACAGCAAAACACCCACAAATTCGACCCTAAATCCAACAAATTCGACCAAAAAACTCCGATTTCCCAGCAAATTCAACATCTAAAATCGCAATCAAGTTCAAGACCCATAATCGGCAGTAAATTCGAGCTATAATCCCCCAATTTCGAGCAAAAATTCAGACCAAAAACCCTAAGAAATTCGAACCCATAAACCCCAGAAAAATTGAGCAATTCCGACCATTTCGAGCAAAAAATCTACTCAAAACCCTAGGAATTCGAACCCTAGAAACCCCAAAAAATTCAATTAAAAATCCTTGCCTCATATTCGAGCAATGACTGAAATTTTCGACTTAATTTGTGAAGTCATTTTTGGATTAATTTTGCCCCCAAAAAACCCGAGGAATTTATAGCAAATTACAGAAGTGTGGCAGTTTCGACTGAATTTGTGAAGTCGATTTCACCTAAGGTAAGATTGCTTCTATTTATGTTCACTCTTTTCATGGTAGTTAGAATGTAGCATCTGACTTGAGCAATGACTTAGTTTAATTTCAGGTTTAGTGTTAGTTCATTAATTAGAAAATATATGGCAGATTTAGTTGAATATATATAATTTCAGTCATTGAATATATGGCAGATTTAGTTGAATCAACTATGTTGGTTTAATATATGTAATTTTATATGGCAGATTTCTTAATTATGCGGAAGTGAATTCCTCAAGTTGACTGAGTTTCTGGTTGTGGACAAACGATTAAGGCGGTAGTTCACATACGAGATTGTCTTCACTTGTGGTATATATACCTGAACCATTCTTTGCAATATTAACTTATTTTTTGTGGTTGGTATAATGATTGTGAATTGTATGAGATTTTTAATTGTGATATGATTGATTGGAATTGCAATAATACTTGCATCATTGGCTTAGATACCCGTTCAGGGCTTACTCATTAGGAGTAAGTCTTGAATAGTCCCCGGTTTGGGACTGTTCTTGAATGTTCTATCTCATTTAGGTGATAACTACTTGAACTGTTTTTGGGTCTAAGAAGATCATGGCTCACTATTTCCCGATCGTTCTCTGGGTACATATGTAGATAGACGATCATCTATATTGTGTGCTTGGAGAACTATAGGGAAATGCTGCCGGATTTTCTTTAGAACCAAACTTTACAAGTAGTTATTAGTGAGATAGGACATTCAAGAACGGGTTAGGTTGGAGTGATAAGGACCTTGTTGGATCATGTCATTGCATTTACATGAGATAAACTTTGAATGAAAATGTTTGATGCCTTGTATTCTTGTTTATGGTTAAGGAACTATATATGTTAGAAAATATACACATTATCTTTAAATTAATTTTTGTTATTCAGTGATTGAAAATGGATCGAAGTTGGATGTATGGTAAACGAAACTGTACAAGATTTTTACGAGGAATTGAAGAGTTTAGCAATGTCGCCTTAAACTACCAAACAGAGAATAAATCAAAGATTATTCTTTGTCCTTGTTGTGACTGTAATAATTCGAGGGGGTATCGTGATATAGATGATATTAAAGATCATCTAATTCGTCGTGGGTTTAAAGAAAACTACACGAGGTGGACGTGGCATGGTGAGAGCATATATAAAGAGGCTAGTTCTAGTTATTGCCCAAGGGAAGATGAGAATCATTGTGATGATGATAATGAACACCAAGGTGAGGATATCGATACTGCTGGTATTGGTGTTGAAAGTGAGAGTGAAGTGGAGAAGGACAGGATAGATGAAATGATGCATGATGTTGAAGACCATTTCACAGAGTGTCCTCAGACATATGATAGTATTTTGAGAGCTGCAGAAACACCGTTATATCCTGGTTGTACAAAATTCACTAAACTTGGTGCTATTATGAAGTTATTCAACTTAAAGGCGAGCAATTGTTGGACCGATAAGGGTTTTACGCAGTTGTTGGAAGCCTTAGTAGAAATGTTTCCCGAAGGGAATGAACTTCCTAACTCCACCTATGAGGCCAAGAAACTTATGTGCCCTTTAGGTATGGAGTATGTGAAGATACATGCTTGTCCCAATGATTGTGTGTTGTATCGAAATGAGTATGCTGATTTGCATGAGTGTCCAAGGTGTGGAGCTTCTCGTTACAAGATGAATGATACTGGTGAGTTGTGTAAGAAAGGGTCTCCAACTAAGGTATTGTGGTATCTTCCAATTATACCGAGATTTAGGCGACTTTTCACAGATGAAAAAAATGCAAAATTATTGAGGTGGCATGCTGATGGGAGGAAGAAAGATGGGTTAATGAGGCATCCGGCTGATTCCCCGCAATGGAGGAACATTGATCGAAAGTTCAAGGACTTTGGTCAAGAAGATCGAAATCTTAGGCTTGGTCTTAGTACAGATGGAATGAACCCATTTGGGACACTTAGTACCCAATATAGCACTTGGCCGGTTCTCCTAACTATCTACAATTTGCCTCCTTGGTTATGCATGAAGTCTAGATACATTATGTTGTCCCTTCTAATATCTGGGCCTAAACAACCTGGAAATGACATTGATGTGTATCTAGCGCCTCTCATTGAAGATTTGAAATTGTTGTGGAATGAAGGTGTCCAAATGTTTGATGCATATAGTAAAACCAATTTCACTTTACGTGCCATGATTTTTTGTACGATAAATGACTTCCCTGCTTATGGGAATTTGTCGGGGTACACTGTGAAGGGAACAACTCCTTGCCCCATCTGTGAAGATGATTTGGAGGCATTACGCCTAGACAATTGTGGCAAGCATGTATACATGGATAATCGCAGACATCTTCCTGAAGACCACCCTTTTCGAAAGAATAAGGATGCATTTAATGGAAAAGTGGAGTTGAGAGAAGCCCGTGGCCCTTTACGTGCAAGTGAGGTTTATCAACGGGTCAAAGACATTGAGAATGAGTTTGGTAAGCCTTACAAAAGCAAATCAAATGGGGGTTACAAGAAGAAGTCTGAGCTATGGTCTCTTCCATATTGGAGACATTTGGAAGTTAGACATTGTCTAGATGTAATGCATATTGAGAAAAATGTTTGTGATGCCATTGTGGGAACTTTATTAAATATGCCAGGAAAGACAAAGGATGGAGTTAAAGTAAGAAAAGACATGGCTGCTATGGGTCGTTCAGAGTTGGCACCCGAATCTCGAGGAAAACGCTGGTATCTTCCCCCAGCCTGCTTCACCTTGTCTAAAAAGGAAAAAACTAGCTTCTGTGAGTCATTGCATGGTTTAAAGGTCCCGGCTGGATATTCTTCTAATTTTCGTAGACTTGTGTCGATGTCTGACTTGAAATTGGGTGGCATGAAATCTCATGATTGTCATGTCTTGATGCAACAATTATTACCAGTTGCAATTCGAGGAATTTTGCCACCTCAAGTGAGGTATACGATTACAAGATTATGTGTCTTTTTTAACACTATCTGTAGCAAGGTCATAAATCCAAGTATTTTAGATGACTTGCAAGCAGATATACTTGAGACAATGTGTCGATTTGAAATGTATTTTCCCCCATCTTTTTTTGACGTGATGCCTCATTTGGTTATTCATCTTGTACGTGAAATTAAACTTTGTGGACCAGTGTGTATGAGATACATGTATCCTTTTGAACGAGAAATGGGTGACTTAAAGGGAAAAGTCATGAATCCGGCCAAACCTGAAGCTAGTATTGTACAGCGAACAGTTGCTGAGGAAGTGGCAGCATGGGTTGCTCAATATCTTGCACGTTCACATAAAATTGGGTTGCCAAAGTCTCGACACGATGGGAGGCTCGGAGGTCAAGGTACTATTGGTAGGAAAAGGATATCCATGGGCTTTGAAATGAAAAATAAGGCCGAGCTTTTTGTGTTGCAAAATCTTAGTGAAGTTCATCCTTACTTGGACGAGCACATGATTTTTCTTAAAAATAAATATCCTTCCAAAAGTGATCTTCAGCTGATAAAGGAGCATAATTGTTCATTCGTTACATGGTTCAAGGAACGAGTGATGTCCCAGCTGTCCACCACACCTAACGATATATCTGACACATTGAGATGGTTGGCATATGGTGCTAAATGTCAAGTCATTTCATATGAGGGATACGACATCAATGGGTATTCTTTTTACACTAGCCAACAAGATGACAAATCAACAATGCAAAATAGTGGTGTTAAAGTAATAGGTTTGTCATCTGAGTATGTTAGTGCACATGATAAAACACTTGTGGATAAGAAGAAATTTTATTATGGAATCATTGAAGAAATAATAGAGCTGGACTATGTTGATTTCAAGATTCCTCTATTCCAATGTAAGTGGGCTGATAGTAGTCGTGGTGTAAAAAAAGATGAACAAGGGAACTTGACCCTTGTGAATCTTGGTCGACGAGGGCATCTAGCTGATCCATTTATATTAGCATCACAAGCAAAGCAAGTGTTTTACATGGCTGACCCAGCTGATTGTAAATGGTCAGTTGTATTAGAAGGTAAAAGAAGGATACTTGGCATTGAAGATGTGGTGGACGAGGAAGAATATGATGAGCAATTTAATGAGTCACCACCTTCCGTTTGGAGCATCCCTCCAATAGTCGATGATTTTGACACAACGTTGAAACGTAAAGATCATAATGAAGGATTTTATGTCGCAAAAGAGAAGAATGAAGTAGGTATGAATTCTGTGATTTTTTCCAATATGTGTTACTAATTGCATAAATTGCTTATTATTGTTATGTGTACAAGTTTATAATTTATAAATGATACAAATCGATGTCTTAATTGATTTATGTCTTTTTTTTTCAGGTAAGTAATAATGGAGGATGAGGATATGCGTAATCGTTCCATGTCTTCGCAAGGTGAGGAAGATGAGCCACACAATCAATCACAATCACAGCAGCAAACAACCGACTCTCAAAAGAAGAAAAAGAAGCCAAGAGGTCCTTCAAAGGGCCTGAAATCCATGCCTGGGGTTCCTAGAGTGCTTGAATGGGATGAATTGTGTCGACCCATTGGAAAGTGGGCAAAAGCATACAAAATTCATCTTGGTGAAATAAGCCGTGCAAAAGTGTCTATATTGTATAAAGATTGGAATCAAGTTCCACAAGGAATAAAAGACACTTTGTGGGAAGATGTTAAGGTAATATTCACTATTAAATGTTAAATGTTATACTATTATATGATCATTTTGAAATTATTTGAATATGAGACTTCCTTTTTTTTATGTAGAGAGAGTTTCAAATCGAAGAAGATGAAAACAAGAAAAAAAAGGTCCTACGCACTTGTGATAAGTGCTGGAGAGATTTTAAAACAAAATTGGTCAGTGGTTGGATCACATGTACAAGGAATATGCCGAAGGAGAAAAGAATGTCGTATGTACTCTATGATTTCATATCTGAAGATATGTGGAAAACATTTGTGGAGGAGCATAGTACAGATGATTTTAAGGTTTGTGTGTGTATTAAAATCAATGAGCATGCATGTTATTAATACTTTGTTAACGGATTCTTATGTACTATTCTCCATTTATGCTTGTGAAAATAGGAAATCAGTGAGAAGGCAAGACAAAGCCAGTCTTTCAACGAATACCCTCACCATTTAGGAGCCAAATCATATGGTGAAATGAATACTGTTTGGCGTAGAAAAGGGTATATTCCCACATCATCTTCAGCATCCAGTACTTCATCTTGTTCTTCGGTTGTGTCTAGTTTGCCGGATAGGACATATGCTTGGCTTCTAGCAAGATCAGTGGAAGATGATAAGGGAAATCCGTATTTGCCGGATGAGAAAACAAGAGAGGTGAAAGAATCCATTGTAAGTATATAAAAATAAGTCTATTTCAATTTTGAATATATTTTGTTAAGTTTATGTATTGCAACGAAAGTTTACTAACTTGAAGTCTTTGTGTAGGATAATTGGAGAAAGCAACAAGCTGATGGGAAATTTGTTCCTAATAGGCATGATGATATCTTATCTCGTGCCCTTGGGAAAAAAGATCGTAACGGTCGGGCAATAGCATTTGGCAGTGGAATTGGCATTAAAGCTGTATGGGGATCCGGAGAGAGGCGTAGTGGCCGACGGGGTAGGGAAATCGGAGATGCTGAGCTGGAAGAATTAGAGGCAAGAGTAACCCGAAGGGTACGAGAGGAGACTATGCAGGAGATGGACTCTAAAATGGATTCCATGGTTCAAGAGAAGTTTATGTTATTTGCTAAACAGATTGGTATTCAAATTCCAACTGAATTGTTGGAAATGAATAACATAAGCCGGACGACTCCACAAAATCCTAGTAGTTGCCAATCAGTGGGTGATGATCCATTTGCAAACATACAGGTATCTAAATTTTTTGTAATGATTTATGTCTGCTATATTATTTAACTTTGTAACTCATTTTTTTTCCAATGATTTTTGTCTATCTTTGTAAGGAACCGGTTCCATGTCGGCTATCATTGTTGAAAAATGACTCTGAGAAAGTCATTGTCGCTGAAGGTACATATCATCCGGAGTTAATCCTTGATCATCATAGTAACCTCCTTCCAGATCACGTGAGGGTAAGTGTTGATGATTTTTTTGACGAGTTCAAAGAATTCTCGGTTCCAGTTCCTTCTTCAGTCATCAAGAAACTTAAGCATGCTCACGGTACCTTTACGCAATGGCCGAAACACTTGGTTTCACTCATGCATGACAAGGTAAATTATTTTTATAACAATTTACATAGTTTGCCATACAAAAATATAGCAAATTGTTGAATAGATTTGCTTTACTATTGATTAGGAATTCATATCAAATAAGAACGATGATAACAGTAAAGCGGCCAAAGAAAACATGCAAGACAAAGTGAAAGAGGTTGAAAGTGGTCACGAAACAAAAGAGTCCAACACCAATCCAAAAAAAGTTTTTCTTATGGATTATGCTTTGGAAAACTTGTCTCAGAAGTGTGGGTCTTTGAATAGTTTGTTATCTTCATTACCCGAAGGTGAAACTATTAAGGTAAAGGTTGATGCATGGACCTTTAGTTATGAAGACAGTAAGGACATCATTATCAGACTTGAAGATGTCAATCAACTTCTCACGGGAGCTTGGCTGAATATTTCAATTTTGCAAGTTTTTATGATGTAAGATATTCAAATTTCACTCTTCCCATATATACTTTATTACCATTTATAATATTATTACATGATATATATGTAGGGCCTTGAGTGACTTACTCGATACGGTGGATGTGGCTTCCATTGGATTCATGTGTCCGGAAATGATTTCAGAAACCTATTTGCATAGTGATGCAGATCGTATCCTACTATACATGACACATGTGATGGAAAAACAAAAATCTAAGCACTTCATCTTATGCCCATACCATGAAAAGTATAAACTTTGAACTTGTATTTATTTGAACTTTGCAAATTGTTGATATTATTTTATTAATTTACAAATGTGTGTCTAATTTATTATGTAGGAATCATTGGGTTCTTTTGGTTTTATGCATGGCTAAGCGTGAAGTCTACATCTTTGATTCTTTGAGGCAAAAGCGAAATTTAGCAATTAAGTTTGCAATGACAAAGTAAGTCTAATTTGTTATGAATGTCATGATTTTATGTGTGTGTAAGGATAATAGATGGTTTTAAATATTATGTATAAATATGTTATTTGTTTCAAATGTGTAGTGCTTTTCGAAGTTACAAGGCATTAAGTGGACAATCTAGGGGAAGTAAATTGACATGGCATTTGGGACAGGTTAACTTAATTCCTTTGCCCAAACTTCTAGTTTCTTAATCATTTAAGTATAGCAATTTAATTCAACTTATAATTGTAATGCATATAGTGTCCTCAGCAATTGGGTGGACGTGAGTGTGGCTACTACGTCATGCGTTATATGTACGAAATACTTGAACATCATCGTAGCAGTGAGGATCTTATAAAGGTATGAGTTGTCAAATATTTCATTTTATTTGATAAATACAATTTAATGTTAGCTACAATCTTGAATTAAAACTTTCCTCTTGTATATTTGTTGTGTTGATAGGATTTTTCAAGAACTACCCCGTATACCGAGGAGGAGATAAATGAGGTTCGAGATATTTGGGCAGAGTATTTTATATGTAATGTTGAACTTTAGATTTACACTTAGCAAATGTTGGACTCTTGTTTTGGATTATTCATTATAGGCTTGACATGAATGAATTTGGTTGATTGGTGTTGGGATGATGAGTATATGTCAATGATATTGAGATGTAAACTTATTGGACAAGTTGTTAATATAATGCCGTCATTCCCAATTTTATGGGTAGGCCAAAATACAAAGGAAACTCTGCCGAAATTTACATTTGAATTCATTAATATTGCATTTTAATTTCATTTATTGGTGTTAGCATATTACGTTATACCACTACCAAGGGAAACAGGTACTACAACAATATACAAGCAATAAATATAATTAAAAAAAAAATTCACATCATTTGCGTCACTCATTAAACAAACAAGTGACGCAAATAACTCGCTTTTGAATTGTCTAGTAAGCCATTTGCGTCACTCAATAGTTAAGCAAGTGACGCAAATTCAAAACAAGAAGTTGCTCAGAACAGTTATTTGCGTCACTCCATTATAAAACAGTGACGCAAATAACTTTCATTTGCGTCACTTGTTTATTAAGGAGTGACGCAAATAACTGTTATTTGCGTCGCGCCTAGGAGCGACGCAAATGATTTAAAAGTCATTTGCGTCGCGAGCAGTTGCGTCGCTCATAGAAGCGACGCAAATACACTATAAATGAGCGACGCAAATGAACGTTTTTCTACTAGTG

Dot-plot analysis:


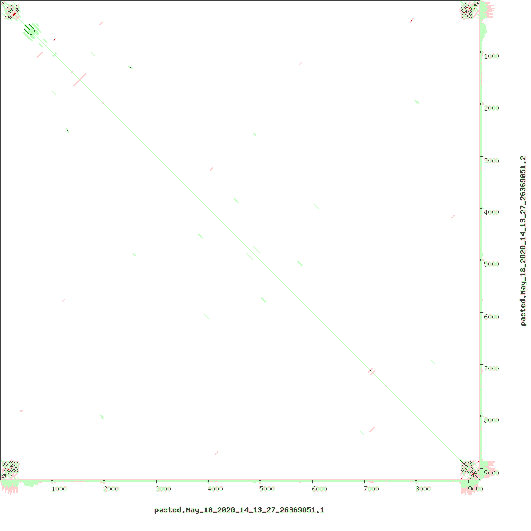


AUGUSTUS analysis:

# coding sequence = [atggatcgaagttggatgtatggtaaacgaaactgtacaagatttttacgaggaattgaagagtttagcaatgtcgccttaaactaccaaacagagaataaatcaaagattattctttgtccttgttgtgactgtaataattcgagggggtatcgtgatatagatgatattaaagatcatctaattcgtcgtgggtttaaagaaaactacacgaggtggacgtggcatggtgagagcatatataaagaggctagttctagttattgcccaagggaagatgagaatcattgtgatgatgataatgaacaccaaggtgaggatatcgatactgctggtattggtgttgaaagtgagagtgaagtggagaaggacaggatagatgaaatgatgcatgatgttgaagaccatttcacagagtgtcctcagacatatgatagtattttgagagctgcagaaacaccgttatatcctggttgtacaaaattcactaaacttggtgctattatgaagttattcaacttaaaggcgagcaattgttggaccgataagggttttacgcagttgttggaagccttagtagaaatgtttcccgaagggaatgaacttcctaactccacctatgaggccaagaaacttatgtgccctttaggtatggagtatgtgaagatacatgcttgtcccaatgattgtgtgttgtatcgaaatgagtatgctgatttgcatgagtgtccaaggtgtggagcttctcgttacaagatgaatgatactggtgagttgtgtaagaaagggtctccaactaaggtattgtggtatcttccaattataccgagatttaggcgacttttcacagatgaaaaaaatgcaaaattattgaggtggcatgctgatgggaggaagaaagatgggttaatgaggcatccggctgattccccgcaatggaggaacattgatcgaaagttcaaggactttggtcaagaagatcgaaatcttaggcttggtcttagtacagatggaatgaacccatttgggacacttagtacccaatatagcacttggccggttctcctaactatctacaatttgcctccttggttatgcatgaagtctagatacattatgttgtcccttctaatatctgggcctaaacaacctggaaatgacattgatgtgtatctagcgcctctcattgaagatttgaaattgttgtggaatgaaggtgtccaaatgtttgatgcatatagtaaaaccaatttcactttacgtgccatgattttttgtacgataaatgacttccctgcttatgggaatttgtcggggtacactgtgaagggaacaactccttgccccatctgtgaagatgatttggaggcattacgcctagacaattgtggcaagcatgtatacatggataatcgcagacatcttcctgaagaccacccttttcgaaagaataaggatgcatttaatggaaaagtggagttgagagaagcccgtggccctttacgtgcaagtgaggtttatcaacgggtcaaagacattgagaatgagtttggtaagccttacaaaagcaaatcaaatgggggttacaagaagaagtctgagctatggtctcttccatattggagacatttggaagttagacattgtctagatgtaatgcatattgagaaaaatgtttgtgatgccattgtgggaactttattaaatatgccaggaaagacaaaggatggagttaaagtaagaaaagacatggctgctatgggtcgttcagagttggcacccgaatctcgaggaaaacgctggtatcttcccccagcctgcttcaccttgtctaaaaaggaaaaaactagcttctgtgagtcattgcatggtttaaaggtcccggctggatattcttctaattttcgtagacttgtgtcgatgtctgacttgaaattgggtggcatgaaatctcatgattgtcatgtcttgatgcaacaattattaccagttgcaattcgaggaattttgccacctcaagtgaggtatacgattacaagattatgtgtcttttttaacactatctgtagcaaggtcataaatccaagtattttagatgacttgcaagcagatatacttgagacaatgtgtcgatttgaaatgtattttcccccatctttttttgacgtgatgcctcatttggttattcatcttgtacgtgaaattaaactttgtggaccagtgtgtatgagatacatgtatccttttgaacgagaaatgggtgacttaaagggaaaagtcatgaatccggccaaacctgaagctagtattgtacagcgaacagttgctgaggaagtggcagcatgggttgctcaatatcttgcacgttcacataaaattgggttgccaaagtctcgacacgatgggaggctcggaggtcaaggtactattggtaggaaaaggatatccatgggctttgaaatgaaaaataaggccgagctttttgtgttgcaaaatcttagtgaagttcatccttacttggacgagcacatgatttttcttaaaaataaatatccttccaaaagtgatcttcagctgataaaggagcataattgttcattcgttacatggttcaaggaacgagtgatgtcccagctgtccaccacacctaacgatatatctgacacattgagatggttggcatatggtgctaaatgtcaagtcatttcatatgagggatacgacatcaatgggtattctttttacactagccaacaagatgacaaatcaacaatgcaaaatagtggtgttaaagtaataggtttgtcatctgagtatgttagtgcacatgataaaacacttgtggataagaagaaattttattatggaatcattgaagaaataatagagctggactatgttgatttcaagattcctctattccaatgtaagtgggctgatagtagtcgtggtgtaaaaaaagatgaacaagggaacttgacccttgtgaatcttggtcgacgagggcatctagctgatccatttatattagcatcacaagcaaagcaagtgttttacatggctgacccagctgattgtaaatggtcagttgtattagaaggtaaaagaaggatacttggcattgaagatgtggtggacgaggaagaatatgatgagcaatttaatgagtcaccaccttccgtttggagcatccctccaatagtcgatgattttgacacaacgttgaaacgtaaagatcataatgaaggattttatgtcgcaaaagagaagaatgaagtaggtgaggaagatgagccacacaatcaatcacaatcacagcagcaaacaaccgactctcaaaagaagaaaaagaagccaagaggtccttcaaagggcctgaaatccatgcctggggttcctagagtgcttgaatgggatgaattgtgtcgacccattggaaagtgggcaaaagcatacaaaattcatcttggtgaaataagccgtgcaaaagtgtctatattgtataaagattggaatcaagttccacaaggaataaaagacactttgtgggaagatgttaagagagagtttcaaatcgaagaagatgaaaacaagaaaaaaaaggtcctacgcacttgtgataagtgctggagagattttaaaacaaaattggtcagtggttggatcacatgtacaaggaatatgccgaaggagaaaagaatgtcgtatgtactctatgatttcatatctgaagatatgtggaaaacatttgtggaggagcatagtacagatgattttaaggaaatcagtgagaaggcaagacaaagccagtctttcaacgaataccctcaccatttaggagccaaatcatatggtgaaatgaatactgtttggcgtagaaaagggtatattcccacatcatcttcagcatccagtacttcatcttgttcttcggttgtgtctagtttgccggataggacatatgcttggcttctagcaagatcagtggaagatgataagggaaatccgtatttgccggatgagaaaacaagagaggtgaaagaatccattgataattggagaaagcaacaagctgatgggaaatttgttcctaataggcatgatgatatcttatctcgtgcccttgggaaaaaagatcgtaacggtcgggcaatagcatttggcagtggaattggcattaaagctgtatggggatccggagagaggcgtagtggccgacggggtagggaaatcggagatgctgagctggaagaattagaggcaagagtaacccgaagggtacgagaggagactatgcaggagatggactctaaaatggattccatggttcaagagaagtttatgttatttgctaaacagattggtattcaaattccaactgaattgttggaaatgaataacataagccggacgactccacaaaatcctagtagttgccaatcagtgggtgatgatccatttgcaaacatacaggaaccggttccatgtcggctatcattgttgaaaaatgactctgagaaagtcattgtcgctgaaggtacatatcatccggagttaatccttgatcatcatagtaacctccttccagatcacgtgagggtaagtgttgatgatttttttgacgagttcaaagaattctcggttccagttccttcttcagtcatcaagaaacttaagcatgctcacggtacctttacgcaatggccgaaacacttggtttcactcatgcatgacaaggaattcatatcaaataagaacgatgataacagtaaagcggccaaagaaaacatgcaagacaaagtgaaagaggttgaaagtggtcacgaaacaaaagagtccaacaccaatccaaaaaaagtttttcttatggattatgctttggaaaacttgtctcagaagtgtgggtctttgaatagtttgttatcttcattacccgaaggtgaaactattaaggtaaaggttgatgcatggacctttagttatgaagacagtaaggacatcattatcagacttgaagatgtcaatcaacttctcacgggagcttggctgaatatttcaattttgcaagtttttatgatggccttgagtgacttactcgatacggtggatgtggcttccattggattcatgtgtccggaaatgatttcagaaacctatttgcatagtgatgcagatcgtatcctactatacatgacacatgtgatggaaaaacaaaaatctaagcacttcatcttatgcccataccatgaaaagaatcattgggttcttttggttttatgcatggctaagcgtgaagtctacatctttgattctttgaggcaaaagcgaaatttagcaattaagtttgcaatgacaaatgcttttcgaagttacaaggcattaagtggacaatctaggggaagtaaattgacatggcatttgggacagtgtcctcagcaattgggtggacgtgagtgtggctactacgtcatgcgttatatgtacgaaatacttgaacatcatcgtagcagtgaggatcttataaaggatttttcaagaactaccccgtataccgaggaggagataaatgaggttcgagatatttgggcagagtattttatatgtaatgttgaactttag]

# protein sequence = [MDRSWMYGKRNCTRFLRGIEEFSNVALNYQTENKSKIILCPCCDCNNSRGYRDIDDIKDHLIRRGFKENYTRWTWHGESIYKEASSSYCPREDENHCDDDNEHQGEDIDTAGIGVESESEVEKDRIDEMMHDVEDHFTECPQTYDSILRAAETPLYPGCTKFTKLGAIMKLFNLKASNCWTDKGFTQLLEALVEMFPEGNELPNSTYEAKKLMCPLGMEYVKIHACPNDCVLYRNEYADLHECPRCGASRYKMNDTGELCKKGSPTKVLWYLPIIPRFRRLFTDEKNAKLLRWHADGRKKDGLMRHPADSPQWRNIDRKFKDFGQEDRNLRLGLSTDGMNPFGTLSTQYSTWPVLLTIYNLPPWLCMKSRYIMLSLLISGPKQPGNDIDVYLAPLIEDLKLLWNEGVQMFDAYSKTNFTLRAMIFCTINDFPAYGNLSGYTVKGTTPCPICEDDLEALRLDNCGKHVYMDNRRHLPEDHPFRKNKDAFNGKVELREARGPLRASEVYQRVKDIENEFGKPYKSKSNGGYKKKSELWSLPYWRHLEVRHCLDVMHIEKNVCDAIVGTLLNMPGKTKDGVKVRKDMAAMGRSELAPESRGKRWYLPPACFTLSKKEKTSFCESLHGLKVPAGYSSNFRRLVSMSDLKLGGMKSHDCHVLMQQLLPVAIRGILPPQVRYTITRLCVFFNTICSKVINPSILDDLQADILETMCRFEMYFPPSFFDVMPHLVIHLVREIKLCGPVCMRYMYPFEREMGDLKGKVMNPAKPEASIVQRTVAEEVAAWVAQYLARSHKIGLPKSRHDGRLGGQGTIGRKRISMGFEMKNKAELFVLQNLSEVHPYLDEHMIFLKNKYPSKSDLQLIKEHNCSFVTWFKERVMSQLSTTPNDISDTLRWLAYGAKCQVISYEGYDINGYSFYTSQQDDKSTMQNSGVKVIGLSSEYVSAHDKTLVDKKKFYYGIIEEIIELDYVDFKIPLFQCKWADSSRGVKKDEQGNLTLVNLGRRGHLADPFILASQAKQVFYMADPADCKWSVVLEGKRRILGIEDVVDEEEYDEQFNESPPSVWSIPPIVDDFDTTLKRKDHNEGFYVAKEKNEVGEEDEPHNQSQSQQQTTDSQKKKKKPRGPSKGLKSMPGVPRVLEWDELCRPIGKWAKAYKIHLGEISRAKVSILYKDWNQVPQGIKDTLWEDVKREFQIEEDENKKKKVLRTCDKCWRDFKTKLVSGWITCTRNMPKEKRMSYVLYDFISEDMWKTFVEEHSTDDFKEISEKARQSQSFNEYPHHLGAKSYGEMNTVWRRKGYIPTSSSASSTSSCSSVVSSLPDRTYAWLLARSVEDDKGNPYLPDEKTREVKESIDNWRKQQADGKFVPNRHDDILSRALGKKDRNGRAIAFGSGIGIKAVWGSGERRSGRRGREIGDAELEELEARVTRRVREETMQEMDSKMDSMVQEKFMLFAKQIGIQIPTELLEMNNISRTTPQNPSSCQSVGDDPFANIQEPVPCRLSLLKNDSEKVIVAEGTYHPELILDHHSNLLPDHVRVSVDDFFDEFKEFSVPVPSSVIKKLKHAHGTFTQWPKHLVSLMHDKEFISNKNDDNSKAAKENMQDKVKEVESGHETKESNTNPKKVFLMDYALENLSQKCGSLNSLLSSLPEGETIKVKVDAWTFSYEDSKDIIIRLEDVNQLLTGAWLNISILQVFMMALSDLLDTVDVASIGFMCPEMISETYLHSDADRILLYMTHVMEKQKSKHFILCPYHEKNHWVLLVLCMAKREVYIFDSLRQKRNLAIKFAMTNAFRSYKALSGQSRGSKLTWHLGQCPQQLGGRECGYYVMRYMYEILEHHRSSEDLIKDFSRTTPYTEEEINEVRDIWAEYFICNVEL]

# end gene g1

###

#

*Batch* analysis:


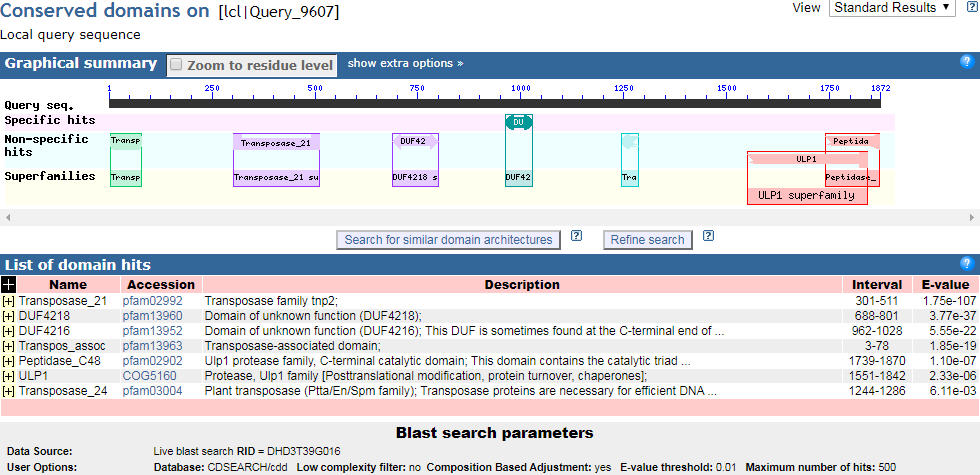


Clone 68TAD-X1(GenBank # MZ325225) from *Chenopodium iljinii* genome analysis (the clone fragments overlaps are highlighted in yellow):

>F4

GCAATGTCGCCTTAAACTACCAAACAGAGAATAAATCAAAGATTATTCTTTGTCCTTGTTGTGACTGTAATAATTCGAGGGGGTATCGTGATATAGATGATATTAAAGATCATCTAATTCGTCGTGGGTTTAAAGAAAACTACACAAGGTGGACGTGGCATGGTGAGAGCATATATAAAGAGGCTAGTTCTAGTTATTGCCCAAGGGAAGATGAGAATCATTGTGATGATGATAATGAACACCAAGGTGAGGATATCGATACTGCTGGTATTGGTGTTGAAAGTGAGAGTGAAGTAGAGAAGGACAGGATAGATGAAATGATGCATTATGTTGAAGACCATTTCACAGAGTGTCCTCAGACATATGATAGTATTTTGAGAGCAGCAGAAACACCGTTATATCCTGGTTGTACAAAATTCACTAAACTTGGTGCTATTATGAAGTTATTCAACTTAAAGGCGAGCAATTGTTGGACCGATAAGGATTTTACGCAGTTGTTGGAAGCCTTAGTAGAAATGTTTCCCGAAGGGAATGAACTTCCTAACTCCACCTATGAGGCCAAGAAACTTATGTGCCCTTTAGGTATGGAGTATGTGAAGATACATGCTTGTCCCAATGATTGTGTGTTGTATCGAAATGAGTATGCTGATTTGCATGAGTGTCCAAGGTGTGGAGCTTCTCGTTACAAGATGAATGATACTGGTGAGTTGTGTAAGAAAGGGTCTCCAACTAAGGTATTGTGGTATCTTCCAATTATACCAAGATTTAGGCGACTTTTCACAGATGAAAGAAATGCAAAATTATTGAGGTGGCATGCTGATGGGAGGAAGAAAGATGGGTTAATGAGGCATCCGGCTGATTCCCCGCAATGGAGGAACATTGATCGAAAGTTCAAGGACTTTGGTCAAGAAGATCGAAATCTTAGGCTTGGTCTTAGTACAGATGGAATGAACCCATTTGGGACACTTAGTACCCAATATAGCACTTGGTCGGTTCTCCTAACTATCTACAATTTGCCTCCTTAGTTATGCATGAAGTCTAGATACATTATGTTGTCCCTTCTAATATCTGGGCCTAAACAACCTGGAAATGACATTGATGTGTATCTAGCGCCTCTCATTGAAGATTTGAAATTGTTATGTAATGAAGGTGTCCAAATGTTTGATGCATATAGTAAAACCAATTTCCCTTTACGTGCCATGATTTTTTGTACGATAAATGACTTCCCTGCTTATGGGAATTTGTCGGGGTA

>F5

TCGGGGTACACTGTGAAGGGAACAACTCCTTGCCCCATCTGTGAAGATGATTTGGAGGCATTACGCCTAGACAATTGTGGCAAGCATGTATACATGGATAATCGCAGACATCTTCCTGAAGACCACCCTTTTCGAAAGAATAAGGATGCATTTAATGGAAAAGTGGAGTTGAGAGAAGCCCGTGGCCCTTTACGTGCAAGTGAGGTTTATCAACGGGTCAAAGACATTGAGAATGAGTTTGGTAAGCCTTACAAAAGCAAATCAAATGGGGGTTACAAGAAGAAGTCTGAGCTATGGTCTCTTCCATATTGGAGACATTTGGAAGTTAGACATTGTCTAGATGTAATGCATATTGAGAAAAATGTTTGTGATGCCATTGTGAGAACTATATTAAATATGCCAGGAAAGACAAAGGATGGAGTTAAAGTAAGAAAAGACATGGCTGCTATGGGTCGTTCAGAGTTGGCACCCGAATCTCGAGGAAAACGCTGGTATCTTCCCCCAGCCTGCTTCACCTTATCGAAAAAGGAAAAAACTAGCTTCTGTGAGTCATTGCATGGTTTAAAGGTCCCGGCTGGATATTCTTCTAATTTTCGTAGACTTGTGTCGATGTCTGACTTGAAATTGGGTGGCATGAAATCTCATGATTGTCATGTCTTGATACAACAATTATTACCAGTTAAAATTCGAGGAATTTTTCACCTCAAGTGAGGTATACGATTACTAGATTATGTGTCTTTTTTAACACTATCTGTAGCAGATATACTTGAGACAATGTGTCGATTTGAAATGTATTTTCCCCCATTTTTTTTCGATGTGATGCCTCATTTGGTTATTCATCTTGTACGTGAAATTAAACTTTGTGTGGACTAGTGTGTATGAGATACATGTATCCTTTTGAACGAGAAATGGGAGACTTAAAGGGAAAAGTCATGAATCCGGCCAAACTTGAAGCTAGTATTGTACAGCGAACAGTTGCTGAGGAAGTGGCAGCATGGGTTGCTCAATATCTTGCACGTTCACATAAAATTGGGTTTCCAAAGTCTCGACACGGTGGGAAGGCTCGGAAGTCAAGGTACTATTGGGTAGGAAAAGGAAATCCTGGGGCTTTGAAATGAAA

>F6

GGGCTTTGAAATGAAATATAAGGCCGAGCTTTTTGTGTTACAAAATCTTAGTGAAGTTCATCCTTACTTGGACGAGCACATGATTTTTCTTAAAAAAAAATATCCTTCCAAAAGTGATCTTCAGCTGATAAAGGAGCATAATTGTTCATTCGTTACATGGTTCAAGGAACGAGTGATGTCCCAGCTGTCCACCACACCTAACGATATATCTGACACATTGAGATGGTTGGCATATGGTACTAAATGTCAAGTCATTTCATATGAGGGATACGACATCAATGGGTATCCTTTCTACACTAGCCAACAAGATGACAAATCAACAATGCAAAATAGTGGTGTTAAAGTAATAGGTTTGTCATCTGAGTATGTTAGTGCACATGATAAAACACTTGTGGATAAGAAGAAATTTTATTATGGAATCATTGAAGAAATAATAGAGCTGGACTATGTTGATTTCAAGATTCCTCTATTCCAATGTAAGTGGGCTGATAGTAGTCGTTGTGTAAAAAAAGATGAACAAGGGAACTTGACCCTTGTGAATCTTGGTCGACGAGGGCATCTAGCTGATCCATTTATATTAGCATCACAAGCAAAGCAAGTGTTTTACATGGCTGACCCAGCTGATTGTAAATGGTCAATTGTATTAGAAGGTAAAAGAAGGATACTTGGCATTGAAGATGTGGTGGACGAGGAAGAATATGATGAGCAATTTAATAAGTCACCACCTTCCGTTTGGAACATCCCTCCAATAGTCGATGATTTTGACACAACGTTGAAACGTAAAGATCATAATGAAGGATTTTATGTCACAAAAGAGAAGAATGAAGTAGGTATGAATTCTGTGATTTTTTTTCCAATATGTGTTACTAATTGCATAAATTGCTTATTATTGTTATGTGTATAATTTTATAATTTATAAATGATACAAATCGATGTCTTAATTGATTTATGTCTTTTTTTTTTCAGGTAAGTAATAATGGAGGATGAGGATATGCGTAATTGTTCCATGTCTTCGCAAGGTGAGGAAGATGAGCCCCACAATCCATCCCAATCACAGCAGCAAA

**From the reverse side:**

>R6

CAATCACAGCAGCAAACCAACCGATTCTCAAAAAGAAGAAAAAAGAAGCCAAGAGGTCCTTTCAAAAGGGGCCTGAAAATCCCATGCCCTAGGGGTTCCCTAGAGTGCTTTGAAATGGGGATGAAATTGTGTCGACCCCATTGGAAAAGTGGGGCAAAAAGCATAGAAAAATTTCATCCTTGGTGAAAATAAAGCCCGTGCAAAAAGTGTCTATATTGTACAAAAGATTGGGAATCAAGTTCCCACAAGGGAATAAAAAGACACTTTTGTGGGGAAGATGTTTAAGGTAATATTCACTATTAAATGTTAAATGTTATACTATTATATGTTCATTTTTGAAATTATTTGAATATGATACTTCCTCTTTTTAATGTAGAGAGAGTTTCAAATCGAAGAAGATGAAAACAAGAAAAAAAAGGTCCTACGCACTTGTGATAAGTGCTGGAGAGATTTTAAAACAAAATTGATCAGTGGTTGGATCACATGTACAAGGAATATGCCTAAGGAGAAAAGAATGCCGTATGTACTCTATGATTTCATATCTGAAGATATGTGGAAAACATTTGTGGAGGAGCATAGTACAGATGATTTTAAGGTTTGTGTGTGTATTAAAAATCAATGAGCATGCATGTTATTTAATACTTTGTTAACGGATTCTTATGTACTATTCTCCATTTATGCTTGTGAAAATAGGAAATCAGTGAGAAGGCAAGACAAAGCCAGTCTTTCAACGAATACCCTCACCATTTAGGAGCCAAATCATATGGTGAAATGAATACTGTTTGGCGTAGAAAAGGGTATATTCCCACATCATCTTCAGCATCCAGTACTTCATCTTGTTCTTCGGTTGTGTCTAGTTTGCCGGATAGGACATATGCTTGGCTTCTAGCAAGATCAGTGGAAGATGATAAGGGAAATCCGTATTTGCCGGATGAGAAAACAAGAGAGGTGAAAGAATCC

>R5

GAAAGAATCCCTTGTAAGTATTAAAAATAAGTCTATTTCAATTTTGAATATATTTTGGTAAAGTTTAGGTATTGCAACGAAAGTTTACTAACTTGAAGTCTTTGTGTAGGATAATTGGAGAAAGCAACAAGCTGATGGGAAATTTGTTCCTAATAGGCATGATGATATCTTATCTCGTGCCCTTGGGAAAAAAGATCGTAACGGTCGGGCAATAGCATTTGGCAGTGGAATTGGCATTAAAGCTGTATGGGGATCCGGAGAGAGGCGTAGTGGCCGACGGGGTAGGGAAATCGGAGATGCTGAGCTGGAAGAATTAGAGGCAAGAGTAACCCGAAGGGTACGAGAGGAGACTATGCAGGAGATGGACTCTAAAATGGATTCCATGGTCAAGAGAAGTTTATGTTATTTGCTAAACAGATTGGTATTTAAATTCCAACTGAATTGTTGGAAATGAATAACATAAGCCGGACGACTCCACAAAATCCTAGTAGTTGCCAATCAGTGGGTGATGATCCATTTGCAAACATACAGGTATCTAAATTTTTTGTAATGATTTATGTCTGCTATATTATTTAACTTTGTAACTCATTTTTTTTCAATGATTTTTGTCTATCTTTGTAAGGAACCGGTTCCATGTCGGCTATCATTGTTGAAAAATGACTCTGAGAAAGTCATTGTCGCTGAAGGTACATATCATCCGGAGTTAATCCTTGATCATCATAATAACCTCCTTCCAGATCACGTGAGGGTAAGTGTTGATGATTTTTTTGACGAGTTCAAAGAATTCTCGGTTCCAGTTCCTTCTTCAGTCATCAAGAAACTTAAGCATGCTCACGGTACCTTTACGCAATGGCCGAAACACTTGGTTTCACTCATGCATGACAAGGTAAATTATTTTTATAACAATTTACATAGTTTGCCATACAAAAATATAGCAAATTGTTGAATAGATTTGCTTTACTATTGATTAGGAATTCATATCAAATAAGAACGATGATAACAGTAAAGCGGCCAAAGAAAACATGCAAGACAAAGTGAAAGAGGTTGAAAGTGGTCACGAAACAAAAGA

>R4

AAACAAAAGAGTCCAACCCCCATTCCAAAAAAAAGTTTTTCTTTATGGATTATGCTTTGGAAAACTTGTCTCAGAAGTGTGGGTCTTTAAATAGTTTGTTATCTTCATTACTTGAAGGTGAAACTATTAAGGTAAAGGTTGATGCATGGACCTTTAGTTATGAAGACAGTAAGGACGTCATTAACAGACATGAAGATGTCAATCAACTTCTCACGGGAGCTTGGCTGAATATTTCAATTTTGCAAGTTTTTATGATGTAAGATATTCAAATTTCACTCTTCCCATATATACTTTATTACCATTTATAATATTATTACATGATATATATGTAGGGCCTTGAGTGACTTACTCGATACGGTGGATGTGGCTTCCATTGGATTCATGTGTCCGGAAATGATTTCAGCAACCTATTTGCATAGAGATGCAGATCGTATCCTAATATACATGACACATGTGATGGAAAAACAAAAATCTAAGCACTTCATCTTATGCCCATACCATGAAAAGTATAAACTTTGAACTTGTATTGATTTGAACTTTGCAAATTGTTGATATTATTTTATTAATTTACAAATGTGTGTCTAATTTATTATGTAGGAATCATTGGGTTCTTTTGGTTTTATGCATGGCTAAGCGTGAAGTCTACATCTTTGATTCTTTGAGGCAAAAGCGAAATTTAGCAATTAAGTTTGCAATGACAAAGTAAGTCTAATTTGTTATGAATGTCATGATTTTATGTGTGTGTAAGGATAATATATGGTTTTAAGTATTATGTATAAATATGTTATTTGTTTCAAATGTGTAGTGCTTTTCGAAGTTACAAGGCATTAAGTGGACAATCTAGAGGAAGTAAATTGACATGGCATTTGGGACAGGTTAACTTAATTCCTTTGCCCAAACTTCTAGTTTCTTAATCATTTAAGTATAGCAATTTAATTCAACTTATAATTGTAATGCATATAGTGTCCTCATCAATTGGGTGGACGTGAGTGTGGCTACTACGTCATGCGTTATATGTACGAAATACTTGAACATCATCGTAGTAGTGAGGATCTTATAAAGGTATGATTTGTAAAATATTTCATTTTATTTGATAAATTACAATTTAATGTTAGCTACAATCTTGAATTAAAACTTTCCTCTTGATATATTTGTTGTGTTGATAGGATTTTCAAGAAGTA

Combined sequence 68TAD-X1 from clones:

GCAATGTCGCCTTAAACTACCAAACAGAGAATAAATCAAAGATTATTCTTTGTCCTTGTTGTGACTGTAATAATTCGAGGGGGTATCGTGATATAGATGATATTAAAGATCATCTAATTCGTCGTGGGTTTAAAGAAAACTACACAAGGTGGACGTGGCATGGTGAGAGCATATATAAAGAGGCTAGTTCTAGTTATTGCCCAAGGGAAGATGAGAATCATTGTGATGATGATAATGAACACCAAGGTGAGGATATCGATACTGCTGGTATTGGTGTTGAAAGTGAGAGTGAAGTAGAGAAGGACAGGATAGATGAAATGATGCATTATGTTGAAGACCATTTCACAGAGTGTCCTCAGACATATGATAGTATTTTGAGAGCAGCAGAAACACCGTTATATCCTGGTTGTACAAAATTCACTAAACTTGGTGCTATTATGAAGTTATTCAACTTAAAGGCGAGCAATTGTTGGACCGATAAGGATTTTACGCAGTTGTTGGAAGCCTTAGTAGAAATGTTTCCCGAAGGGAATGAACTTCCTAACTCCACCTATGAGGCCAAGAAACTTATGTGCCCTTTAGGTATGGAGTATGTGAAGATACATGCTTGTCCCAATGATTGTGTGTTGTATCGAAATGAGTATGCTGATTTGCATGAGTGTCCAAGGTGTGGAGCTTCTCGTTACAAGATGAATGATACTGGTGAGTTGTGTAAGAAAGGGTCTCCAACTAAGGTATTGTGGTATCTTCCAATTATACCAAGATTTAGGCGACTTTTCACAGATGAAAGAAATGCAAAATTATTGAGGTGGCATGCTGATGGGAGGAAGAAAGATGGGTTAATGAGGCATCCGGCTGATTCCCCGCAATGGAGGAACATTGATCGAAAGTTCAAGGACTTTGGTCAAGAAGATCGAAATCTTAGGCTTGGTCTTAGTACAGATGGAATGAACCCATTTGGGACACTTAGTACCCAATATAGCACTTGGTCGGTTCTCCTAACTATCTACAATTTGCCTCCTTAGTTATGCATGAAGTCTAGATACATTATGTTGTCCCTTCTAATATCTGGGCCTAAACAACCTGGAAATGACATTGATGTGTATCTAGCGCCTCTCATTGAAGATTTGAAATTGTTATGTAATGAAGGTGTCCAAATGTTTGATGCATATAGTAAAACCAATTTCCCTTTACGTGCCATGATTTTTTGTACGATAAATGACTTCCCTGCTTATGGGAATTTGTCGGGGTACACTGTGAAGGGAACAACTCCTTGCCCCATCTGTGAAGATGATTTGGAGGCATTACGCCTAGACAATTGTGGCAAGCATGTATACATGGATAATCGCAGACATCTTCCTGAAGACCACCCTTTTCGAAAGAATAAGGATGCATTTAATGGAAAAGTGGAGTTGAGAGAAGCCCGTGGCCCTTTACGTGCAAGTGAGGTTTATCAACGGGTCAAAGACATTGAGAATGAGTTTGGTAAGCCTTACAAAAGCAAATCAAATGGGGGTTACAAGAAGAAGTCTGAGCTATGGTCTCTTCCATATTGGAGACATTTGGAAGTTAGACATTGTCTAGATGTAATGCATATTGAGAAAAATGTTTGTGATGCCATTGTGAGAACTATATTAAATATGCCAGGAAAGACAAAGGATGGAGTTAAAGTAAGAAAAGACATGGCTGCTATGGGTCGTTCAGAGTTGGCACCCGAATCTCGAGGAAAACGCTGGTATCTTCCCCCAGCCTGCTTCACCTTATCGAAAAAGGAAAAAACTAGCTTCTGTGAGTCATTGCATGGTTTAAAGGTCCCGGCTGGATATTCTTCTAATTTTCGTAGACTTGTGTCGATGTCTGACTTGAAATTGGGTGGCATGAAATCTCATGATTGTCATGTCTTGATACAACAATTATTACCAGTTAAAATTCGAGGAATTTTTCACCTCAAGTGAGGTATACGATTACTAGATTATGTGTCTTTTTTAACACTATCTGTAGCAGATATACTTGAGACAATGTGTCGATTTGAAATGTATTTTCCCCCATTTTTTTTCGATGTGATGCCTCATTTGGTTATTCATCTTGTACGTGAAATTAAACTTTGTGTGGACTAGTGTGTATGAGATACATGTATCCTTTTGAACGAGAAATGGGAGACTTAAAGGGAAAAGTCATGAATCCGGCCAAACTTGAAGCTAGTATTGTACAGCGAACAGTTGCTGAGGAAGTGGCAGCATGGGTTGCTCAATATCTTGCACGTTCACATAAAATTGGGTTTCCAAAGTCTCGACACGGTGGGAAGGCTCGGAAGTCAAGGTACTATTGGGTAGGAAAAGGAAATCCTGGGGCTTTGAAATGAAATATAAGGCCGAGCTTTTTGTGTTACAAAATCTTAGTGAAGTTCATCCTTACTTGGACGAGCACATGATTTTTCTTAAAAAAAAATATCCTTCCAAAAGTGATCTTCAGCTGATAAAGGAGCATAATTGTTCATTCGTTACATGGTTCAAGGAACGAGTGATGTCCCAGCTGTCCACCACACCTAACGATATATCTGACACATTGAGATGGTTGGCATATGGTACTAAATGTCAAGTCATTTCATATGAGGGATACGACATCAATGGGTATCCTTTCTACACTAGCCAACAAGATGACAAATCAACAATGCAAAATAGTGGTGTTAAAGTAATAGGTTTGTCATCTGAGTATGTTAGTGCACATGATAAAACACTTGTGGATAAGAAGAAATTTTATTATGGAATCATTGAAGAAATAATAGAGCTGGACTATGTTGATTTCAAGATTCCTCTATTCCAATGTAAGTGGGCTGATAGTAGTCGTTGTGTAAAAAAAGATGAACAAGGGAACTTGACCCTTGTGAATCTTGGTCGACGAGGGCATCTAGCTGATCCATTTATATTAGCATCACAAGCAAAGCAAGTGTTTTACATGGCTGACCCAGCTGATTGTAAATGGTCAATTGTATTAGAAGGTAAAAGAAGGATACTTGGCATTGAAGATGTGGTGGACGAGGAAGAATATGATGAGCAATTTAATAAGTCACCACCTTCCGTTTGGAACATCCCTCCAATAGTCGATGATTTTGACACAACGTTGAAACGTAAAGATCATAATGAAGGATTTTATGTCACAAAAGAGAAGAATGAAGTAGGTATGAATTCTGTGATTTTTTTTCCAATATGTGTTACTAATTGCATAAATTGCTTATTATTGTTATGTGTATAATTTTATAATTTATAAATGATACAAATCGATGTCTTAATTGATTTATGTCTTTTTTTTTTCAGGTAAGTAATAATGGAGGATGAGGATATGCGTAATTGTTCCATGTCTTCGCAAGGTGAGGAAGATGAGCCCCACAATCCATCCCAATCACAGCAGCAAACCAACCGATTCTCAAAAAGAAGAAAAAAGAAGCCAAGAGGTCCTTTCAAAAGGGGCCTGAAAATCCCATGCCCTAGGGGTTCCCTAGAGTGCTTTGAAATGGGGATGAAATTGTGTCGACCCCATTGGAAAAGTGGGGCAAAAAGCATAGAAAAATTTCATCCTTGGTGAAAATAAAGCCCGTGCAAAAAGTGTCTATATTGTACAAAAGATTGGGAATCAAGTTCCCACAAGGGAATAAAAAGACACTTTTGTGGGGAAGATGTTTAAGGTAATATTCACTATTAAATGTTAAATGTTATACTATTATATGTTCATTTTTGAAATTATTTGAATATGATACTTCCTCTTTTTAATGTAGAGAGAGTTTCAAATCGAAGAAGATGAAAACAAGAAAAAAAAGGTCCTACGCACTTGTGATAAGTGCTGGAGAGATTTTAAAACAAAATTGATCAGTGGTTGGATCACATGTACAAGGAATATGCCTAAGGAGAAAAGAATGCCGTATGTACTCTATGATTTCATATCTGAAGATATGTGGAAAACATTTGTGGAGGAGCATAGTACAGATGATTTTAAGGTTTGTGTGTGTATTAAAAATCAATGAGCATGCATGTTATTTAATACTTTGTTAACGGATTCTTATGTACTATTCTCCATTTATGCTTGTGAAAATAGGAAATCAGTGAGAAGGCAAGACAAAGCCAGTCTTTCAACGAATACCCTCACCATTTAGGAGCCAAATCATATGGTGAAATGAATACTGTTTGGCGTAGAAAAGGGTATATTCCCACATCATCTTCAGCATCCAGTACTTCATCTTGTTCTTCGGTTGTGTCTAGTTTGCCGGATAGGACATATGCTTGGCTTCTAGCAAGATCAGTGGAAGATGATAAGGGAAATCCGTATTTGCCGGATGAGAAAACAAGAGAGGTGAAAGAATCCCTTGTAAGTATTAAAAATAAGTCTATTTCAATTTTGAATATATTTTGGTAAAGTTTAGGTATTGCAACGAAAGTTTACTAACTTGAAGTCTTTGTGTAGGATAATTGGAGAAAGCAACAAGCTGATGGGAAATTTGTTCCTAATAGGCATGATGATATCTTATCTCGTGCCCTTGGGAAAAAAGATCGTAACGGTCGGGCAATAGCATTTGGCAGTGGAATTGGCATTAAAGCTGTATGGGGATCCGGAGAGAGGCGTAGTGGCCGACGGGGTAGGGAAATCGGAGATGCTGAGCTGGAAGAATTAGAGGCAAGAGTAACCCGAAGGGTACGAGAGGAGACTATGCAGGAGATGGACTCTAAAATGGATTCCATGGTCAAGAGAAGTTTATGTTATTTGCTAAACAGATTGGTATTTAAATTCCAACTGAATTGTTGGAAATGAATAACATAAGCCGGACGACTCCACAAAATCCTAGTAGTTGCCAATCAGTGGGTGATGATCCATTTGCAAACATACAGGTATCTAAATTTTTTGTAATGATTTATGTCTGCTATATTATTTAACTTTGTAACTCATTTTTTTTCAATGATTTTTGTCTATCTTTGTAAGGAACCGGTTCCATGTCGGCTATCATTGTTGAAAAATGACTCTGAGAAAGTCATTGTCGCTGAAGGTACATATCATCCGGAGTTAATCCTTGATCATCATAATAACCTCCTTCCAGATCACGTGAGGGTAAGTGTTGATGATTTTTTTGACGAGTTCAAAGAATTCTCGGTTCCAGTTCCTTCTTCAGTCATCAAGAAACTTAAGCATGCTCACGGTACCTTTACGCAATGGCCGAAACACTTGGTTTCACTCATGCATGACAAGGTAAATTATTTTTATAACAATTTACATAGTTTGCCATACAAAAATATAGCAAATTGTTGAATAGATTTGCTTTACTATTGATTAGGAATTCATATCAAATAAGAACGATGATAACAGTAAAGCGGCCAAAGAAAACATGCAAGACAAAGTGAAAGAGGTTGAAAGTGGTCACGAAACAAAAGAGTCCAACCCCCATTCCAAAAAAAAGTTTTTCTTTATGGATTATGCTTTGGAAAACTTGTCTCAGAAGTGTGGGTCTTTAAATAGTTTGTTATCTTCATTACTTGAAGGTGAAACTATTAAGGTAAAGGTTGATGCATGGACCTTTAGTTATGAAGACAGTAAGGACGTCATTAACAGACATGAAGATGTCAATCAACTTCTCACGGGAGCTTGGCTGAATATTTCAATTTTGCAAGTTTTTATGATGTAAGATATTCAAATTTCACTCTTCCCATATATACTTTATTACCATTTATAATATTATTACATGATATATATGTAGGGCCTTGAGTGACTTACTCGATACGGTGGATGTGGCTTCCATTGGATTCATGTGTCCGGAAATGATTTCAGCAACCTATTTGCATAGAGATGCAGATCGTATCCTAATATACATGACACATGTGATGGAAAAACAAAAATCTAAGCACTTCATCTTATGCCCATACCATGAAAAGTATAAACTTTGAACTTGTATTGATTTGAACTTTGCAAATTGTTGATATTATTTTATTAATTTACAAATGTGTGTCTAATTTATTATGTAGGAATCATTGGGTTCTTTTGGTTTTATGCATGGCTAAGCGTGAAGTCTACATCTTTGATTCTTTGAGGCAAAAGCGAAATTTAGCAATTAAGTTTGCAATGACAAAGTAAGTCTAATTTGTTATGAATGTCATGATTTTATGTGTGTGTAAGGATAATATATGGTTTTAAGTATTATGTATAAATATGTTATTTGTTTCAAATGTGTAGTGCTTTTCGAAGTTACAAGGCATTAAGTGGACAATCTAGAGGAAGTAAATTGACATGGCATTTGGGACAGGTTAACTTAATTCCTTTGCCCAAACTTCTAGTTTCTTAATCATTTAAGTATAGCAATTTAATTCAACTTATAATTGTAATGCATATAGTGTCCTCATCAATTGGGTGGACGTGAGTGTGGCTACTACGTCATGCGTTATATGTACGAAATACTTGAACATCATCGTAGTAGTGAGGATCTTATAAAGGTATGATTTGTAAAATATTTCATTTTATTTGATAAATTACAATTTAATGTTAGCTACAATCTTGAATTAAAACTTTCCTCTTGATATATTTGTTGTGTTGATAGGATTTTCAAGAAGTA

AUGUSTUS analysis:

# coding sequence = [atgatattaaagatcatctaattcgtcgtgggtttaaagaaaactacacaaggtggacgtggcatggtgagagcatatataaagaggctagttctagttattgcccaagggaagatgagaatcattgtgatgatgataatgaacaccaaggtgaggatatcgatactgctggtattggtgttgaaagtgagagtgaagtagagaaggacaggatagatgaaatgatgcattatgttgaagaccatttcacagagtgtcctcagacatatgatagtattttgagagcagcagaaacaccgttatatcctggttgtacaaaattcactaaacttggtgctattatgaagttattcaacttaaaggcgagcaattgttggaccgataaggattttacgcagttgttggaagccttagtagaaatgtttcccgaagggaatgaacttcctaactccacctatgaggccaagaaacttatgtgccctttaggtatggagtatgtgaagatacatgcttgtcccaatgattgtgtgttgtatcgaaatgagtatgctgatttgcatgagtgtccaaggtgtggagcttctcgttacaagatgaatgatactggtgagttgtgtaagaaagggtctccaactaaggtattgtggtatcttccaattataccaagatttaggcgacttttcacagatgaaagaaatgcaaaattattgaggtggcatgctgatgggaggaagaaagatgggttaatgaggcatccggctgattccccgcaatggaggaacattgatcgaaagttcaaggactttggtcaagaagatcgaaatcttaggcttggtcttagtacagatggaatgaacccatttgggacacttagtacccaatatagcacttgtaaaaccaatttccctttacgtgccatgattttttgtacgataaatgacttccctgcttatgggaatttgtcggggtacactgtgaagggaacaactccttgccccatctgtgaagatgatttggaggcattacgcctagacaattgtggcaagcatgtatacatggataatcgcagacatcttcctgaagaccacccttttcgaaagaataaggatgcatttaatggaaaagtggagttgagagaagcccgtggccctttacgtgcaagtgaggtttatcaacgggtcaaagacattgagaatgagtttggtaagccttacaaaagcaaatcaaatgggggttacaagaagaagtctgagctatggtctcttccatattggagacatttggaagttagacattgtctagatgtaatgcatattgagaaaaatgtttgtgatgccattgtgagaactatattaaatatgccaggaaagacaaaggatggagttaaagtaagaaaagacatggctgctatgggtcgttcagagttggcacccgaatctcgaggaaaacgctggtatcttcccccagcctgcttcaccttatcgaaaaaggaaaaaactagcttcttgtgtatgagatacatgtatccttttgaacgagaaatgggagacttaaagggaaaagtcatgaatccggccaaacttgaagctagtattgtacagcgaacagttgctgaggaagtggcagcatgggttgctcaatatcttgcacgttcacataaaattgggtttccaaagtctcgacacggtgggaaggctcggaagtcaagtgaagttcatccttacttggacgagcacatgatttttcttaaaaaaaaatatccttccaaaagtgatcttcagctgataaaggagcataattgttcattcgttacatggttcaaggaacgagtgatgtcccagctgtccaccacacctaacgatatatctgacacattgagatggttggcatatggtactaaatgtcaagtcatttcatatgagggatacgacatcaatgggtatcctttctacactagccaacaagatgacaaatcaacaatgcaaaatagtggtgttaaagtaataggtttgtcatctgagtatgttagtgcacatgataaaacacttgtggataagaagaaattttattatggaatcattgaagaaataatagagctggactatgttgatttcaagattcctctattccaatgtaagtgggctgatagtagtcgttgtgtaaaaaaagatgaacaagggaacttgacccttgtgaatcttggtcgacgagggcatctagctgatccatttatattagcatcacaagcaaagcaagtgttttacatggctgacccagctgattgtaaatggtcaattgtattagaaggtaaaagaaggatacttggcattgaagatgtggtggacgaggaagaatatgatgagcaatttaataagtcaccaccttccgtttggaacatccctccaatagtcgatgattttgacacaacgttgaaacgtaaagatcataatgaaggattttatgtcacaaaagagaagaatgaaagagagtttcaaatcgaagaagatgaaaacaagaaaaaaaaggtcctacgcacttgtgataagtgctggagagattttaaaacaaaattgatcagtggttggatcacatgtacaaggaatatgcctaaggagaaaagaatgccgtatgtactctatgatttcatatctgaagatatgtggaaaacatttgtggaggagcatagtacagatgattttaaggaaatcagtgagaaggcaagacaaagccagtctttcaacgaataccctcaccatttaggagccaaatcatatggtgaaatgaatactgtttggcgtagaaaagggtatattcccacatcatcttcagcatccagtacttcatcttgttcttcggttgtgtctagtttgccggataggacatatgcttggcttctagcaagatcagtggaagatgataagggaaatccgtatttgccggatgagaaaacaagagaggtgaaagaatcccttgataattggagaaagcaacaagctgatgggaaatttgttcctaataggcatgatgatatcttatctcgtgcccttgggaaaaaagatcgtaacggtcgggcaatagcatttggcagtggaattggcattaaagctgtatggggatccggagagaggcgtagtggccgacggggtagggaaatcggagatgctgagctggaagaattagaggcaagagtaacccgaagggtacgagaggagactatgcaggagatggactctaaaatggattccatggtcaagagaagtttatgttatttgctaaacagattggaaccggttccatgtcggctatcattgttgaaaaatgactctgagaaagtcattgtcgctgaaggtacatatcatccggagttaatccttgatcatcataataacctccttccagatcacgtgagggtaagtgttgatgatttttttgacgagttcaaagaattctcggttccagttccttcttcagtcatcaagaaacttaagcatgctcacggtacctttacgcaatggccgaaacacttggtttcactcatgcatgacaaggaattcatatcaaataagaacgatgataacagtaaagcggccaaagaaaacatgcaagacaaagtgaaagaggttgaaagtggtgaaactattaaggtaaaggttgatgcatggacctttagttatgaagacagtaaggacgtcattaacagacatgaagatgtcaatcaacttctcacgggagcttggctgaatatttcaattttgcaagtttttatgatggccttgagtgacttactcgatacggtggatgtggcttccattggattcatgtgtccggaaatgatttcagcaacctatttgcatagagatgcagatcgtatcctaatatacatgacacatgtgatggaaaaacaaaaatctaagcacttcatcttatgcccataccatgaaaagaatcattgggttcttttggttttatgcatggctaagcgtgaagtctacatctttgattctttgaggcaaaagcgaaatttagcaattaagtttgcaatgacaaatgcttttcgaagttacaaggcattaagtggacaatctagaggaagtaaattgacatggcatttgggacagtgtcctcatcaattgggtggacgtgagtgtggctactacgtcatgcgttatatgtacgaaatacttgaacatcatcgtagtagtgaggatcttataaaggtatga]

# protein sequence = [DIKDHLIRRGFKENYTRWTWHGESIYKEASSSYCPREDENHCDDDNEHQGEDIDTAGIGVESESEVEKDRIDEMMHYVEDHFTECPQTYDSILRAAETPLYPGCTKFTKLGAIMKLFNLKASNCWTDKDFTQLLEALVEMFPEGNELPNSTYEAKKLMCPLGMEYVKIHACPNDCVLYRNEYADLHECPRCGASRYKMNDTGELCKKGSPTKVLWYLPIIPRFRRLFTDERNAKLLRWHADGRKKDGLMRHPADSPQWRNIDRKFKDFGQEDRNLRLGLSTDGMNPFGTLSTQYSTCKTNFPLRAMIFCTINDFPAYGNLSGYTVKGTTPCPICEDDLEALRLDNCGKHVYMDNRRHLPEDHPFRKNKDAFNGKVELREARGPLRASEVYQRVKDIENEFGKPYKSKSNGGYKKKSELWSLPYWRHLEVRHCLDVMHIEKNVCDAIVRTILNMPGKTKDGVKVRKDMAAMGRSELAPESRGKRWYLPPACFTLSKKEKTSFLCMRYMYPFEREMGDLKGKVMNPAKLEASIVQRTVAEEVAAWVAQYLARSHKIGFPKSRHGGKARKSSEVHPYLDEHMIFLKKKYPSKSDLQLIKEHNCSFVTWFKERVMSQLSTTPNDISDTLRWLAYGTKCQVISYEGYDINGYPFYTSQQDDKSTMQNSGVKVIGLSSEYVSAHDKTLVDKKKFYYGIIEEIIELDYVDFKIPLFQCKWADSSRCVKKDEQGNLTLVNLGRRGHLADPFILASQAKQVFYMADPADCKWSIVLEGKRRILGIEDVVDEEEYDEQFNKSPPSVWNIPPIVDDFDTTLKRKDHNEGFYVTKEKNEREFQIEEDENKKKKVLRTCDKCWRDFKTKLISGWITCTRNMPKEKRMPYVLYDFISEDMWKTFVEEHSTDDFKEISEKARQSQSFNEYPHHLGAKSYGEMNTVWRRKGYIPTSSSASSTSSCSSVVSSLPDRTYAWLLARSVEDDKGNPYLPDEKTREVKESLDNWRKQQADGKFVPNRHDDILSRALGKKDRNGRAIAFGSGIGIKAVWGSGERRSGRRGREIGDAELEELEARVTRRVREETMQEMDSKMDSMVKRSLCYLLNRLEPVPCRLSLLKNDSEKVIVAEGTYHPELILDHHNNLLPDHVRVSVDDFFDEFKEFSVPVPSSVIKKLKHAHGTFTQWPKHLVSLMHDKEFISNKNDDNSKAAKENMQDKVKEVESGETIKVKVDAWTFSYEDSKDVINRHEDVNQLLTGAWLNISILQVFMMALSDLLDTVDVASIGFMCPEMISATYLHRDADRILIYMTHVMEKQKSKHFILCPYHEKNHWVLLVLCMAKREVYIFDSLRQKRNLAIKFAMTNAFRSYKALSGQSRGSKLTWHLGQCPHQLGGRECGYYVMRYMYEILEHHRSSEDLIKV]

# end gene g1

###

*Batch* analysis:


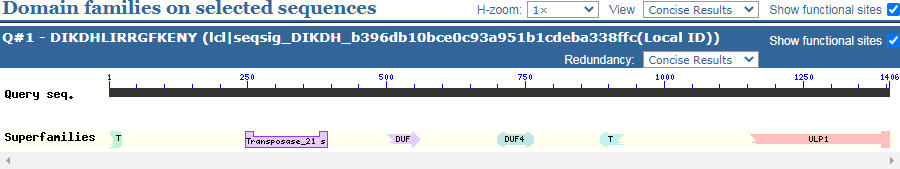

Supplement: Supplementary file 6 — Additional file 6 Comparative analysis of contigs and clones form Chenopodium iljinii genome. [file 13100_2022_265_MOESM6_ESM.docx]
