# Supplementary material for: The structural diversity of CACTA transposons in genomes of Chenopodium (Amaranthaceae, Caryophyllales) species: specific traits and comparison with the similar elements of angiosperms
Source: Mob DNA. 2022 Apr 4;13:8. doi: 10.1186/s13100-022-00265-3 (PMC8978399; doi:10.1186/s13100-022-00265-3)
Supplement: Supplementary file 7 — Additional file 7. Primer pairs used in this study. [file 13100_2022_265_MOESM7_ESM.docx]

S7. Primer pairs used in this study

| No | Content | Position | Left primer (F) | Right primer (R) | DNA of species | Contig |
| --- | --- | --- | --- | --- | --- | --- |
| Conserved Domains Architecture primers | | | | | | |
| 1 | 4 CDs + TPase 24 | start 18 TDA; reverse start 342 TPase 24 | agatacgatggccgtaatgc | ttcctaccaccaacgacctc | *C. iljinii* | *C. iljinii 1* |
| 2 |  |  | accccttataacttgcctcca | accattttctttgcatgcttt |  |  |
| 3 |  |  | tccgtgacctttgttgttcc | tcatcctcatcctcgtcctc |  |  |
| 4 | 4 CDs + TPase 24 + peptidase C48 | start 68 TDA; reverse start 342 peptidase C48 | gcaatgtcgccttaaactacc | tgcccaaatatctcgaacct | *C. iljinii* | *C. iljinii 2* |
| 5 |  |  | ccctttacgtgccatgattt | tccatgcatcaacctttacct |  |  |
| 6 |  |  | gaaggctcggaagtcaaggt | tcagcttgttgctttctcca |  |  |
| satDNA family captured by CACTA-like element | | | | | | |
| 7 | satDNA quinoa | 11 - 165 | ttgaaattgcgtcccaaaac | aaggccccattttggctactagg | *C. quinoa* |  |
| 8 | satDNA sosnowskyi | 6 - 164 | tttggttgaaaatgcgtcct | ggcctatatgacttctaccttgc | *C. sosnowskyi* |  |
